# The CCR4-NOT Complex Is Implicated in the Viability of Aneuploid Yeasts

**DOI:** 10.1371/journal.pgen.1002776

**Published:** 2012-06-21

**Authors:** Yoshie Tange, Atsushi Kurabayashi, Bunshiro Goto, Kwang-Lae Hoe, Dong-Uk Kim, Han-Oh Park, Jacqueline Hayles, Yuji Chikashige, Chihiro Tsutumi, Yasushi Hiraoka, Fumiaki Yamao, Paul Nurse, Osami Niwa

**Affiliations:** 1 Kazusa DNA Research Institute, Kisarazu, Chiba, Japan; 2 Graduate School of Frontier Biosciences, Osaka University, Suita, Osaka, Japan; 3 Chungnam National University, Graduate School of New Drug Discovery and Development, Yusong-gu, Daejeon, Korea; 4 Aging Research Center, Korea Research Institute of Bioscience and Biotechnology (KRIBB), Yusong-gu, Daejeon, Korea; 5 Bioneer Corporation, Daejeon, Korea; 6 Cancer Research UK, The London Research Institute, London, United Kingdom; 7 National Institute of Information and Communications Technology, Kobe, Japan; 8 National Institute of Genetics, Mishima, Shizuoka, Japan; 9 The Rockefeller University, New York, New York, United States of America; Massachusetts Institute of Technology, United States of America

## Abstract

To identify the genes required to sustain aneuploid viability, we screened a deletion library of non-essential genes in the fission yeast *Schizosaccharomyces pombe*, in which most types of aneuploidy are eventually lethal to the cell. Aneuploids remain viable for a period of time and can form colonies by reducing the extent of the aneuploidy. We hypothesized that a reduction in colony formation efficiency could be used to screen for gene deletions that compromise aneuploid viability. Deletion mutants were used to measure the effects on the viability of spores derived from triploid meiosis and from a chromosome instability mutant. We found that the CCR4-NOT complex, an evolutionarily conserved general regulator of mRNA turnover, and other related factors, including poly(A)-specific nuclease for mRNA decay, are involved in aneuploid viability. Defective mutations in CCR4-NOT complex components in the distantly related yeast *Saccharomyces cerevisiae* also affected the viability of spores produced from triploid cells, suggesting that this complex has a conserved role in aneuploids. In addition, our findings suggest that the genes required for homologous recombination repair are important for aneuploid viability.

## Author Summary

Aneuploidy is a major cause of abortive development and is implicated in tumorigenesis in humans. Recent studies revealed that the increased need for protein degradation might account for the detrimental effects of aneuploidy on a cell. Here, we investigated the genetic systems responsible for aneuploid viability. Using a collection of gene deletions in fission yeast, we isolated mutants that affect aneuploid viability. We found that an evolutionarily conserved transcription regulator, the CCR4-NOT complex, and its related factors are required for aneuploid viability, suggesting that regulation of mRNA turnover is required to tolerate aneuploidy. In addition, homologous recombination repair is important for aneuploid viability.

## Introduction

Aneuploidy is defined as a deviation from a multiple of the basic chromosome number and is a major cause of developmental defects in animals and humans [1]. Aneuploidy is implicated in tumorigenesis [2]. Aneuploidy is caused by errors in chromosome transmission and generally occurs at a low rate, but rates increase when chromosome transmission fidelity is perturbed, e.g., by mutations at the spindle assembly checkpoint [2]. Polyploidy is related to aneuploid production; e.g., tetraploid cells generated by cell fusion are an efficient source of aneuploid cells [3]. Crosses between polyploids lead to aneuploid gametes in plants [4], [5].

Aneuploidy causes a range of phenotypic consequences and is usually detrimental to both cells and organisms (reviewed in [6]). For example, mouse embryonic fibroblast lines with an extra chromosome have cell proliferation defects [7], and in the yeasts *Saccharomyces cerevisiae* and *Schizosaccharomyces pombe*, aneuploid cells generally show defects in cell cycle progression and genome stability [8]–[10]. As the grade of aneuploidy increases, i.e., the number of chromosomes involved increases, aneuploidy becomes lethal to the cell [8], [9], [11]–[13]. In addition, certain types of aneuploids grow better in suboptimal conditions, e.g., under elevated genotoxic stress [13]. Aneuploidy affects development of the organism in various species across kingdoms [6]. In one model, aneuploid cells are proposed to contain excess proteins that do not participate in protein complexes because of a dosage imbalance in gene products [6]. This idea is consistent with the fact that many aneuploids are sensitive to proteasome inhibitors and to conditions that interfere with protein chaperone function [9], and that among mutations that improve the fitness of aneuploid cells, one is defective in a deubiquitinating enzyme [14].

In *S. cerevisiae* and *S. pombe*, the higher the grade of aneuploidy, the poorer the cell viability. *S. cerevisiae* (n = 16) generally does not tolerate aneuploidy if the number of extra chromosomes exceeds five [11], [12], while in *S. pombe* (n = 3) all six types of aneuploids between n and 2n are lethal or extremely unstable, except for cells disomic for chromosome 3, the smallest of its chromosomes [15]. Aneuploids with higher grades of aneuploidy do not necessarily die immediately; some sustain their viability for a period of time and may survive to form a colony. This can occur when the grade of aneuploidy is reduced, probably by incorrect mitotic chromosome segregation, the rate of which is increased in aneuploid cells [8], [10]–[12]. Given this, we reasoned that compromise of any gene that functions to sustain aneuploid viability will reduce the efficiency of colony formation from the aneuploid cells.

To identify such genes, we screened mutants in *S. pombe* that affect the viability of aneuploid cells using a collection of deletion mutants of non-essential genes. Our results suggest that an evolutionarily conserved protein complex, CCR4-NOT, which is central to the regulation of mRNA turnover, is necessary for aneuploid viability in both fission yeast and budding yeast. Further, other genes involved in mRNA decay and export were identified. We also show that homologous recombination repair is important for the survival of aneuploid cells.

## Results

### Screening of mutants that affect aneuploid cell viability

To identify genes involved in the viability of aneuploid cells, we screened a collection of fission yeast deletion mutants (Materials and Methods) [16] by investigating either spores from triploid meiosis or mutants in which the γ-tubulin gene (*gtb1*) and a spindle checkpoint gene (*mad2*) are impaired, which are referred to as the “triploid meiosis method” and “gtub-mad2 method”, respectively, hereafter.

#### Triploid meiosis method

**Figure 1:**
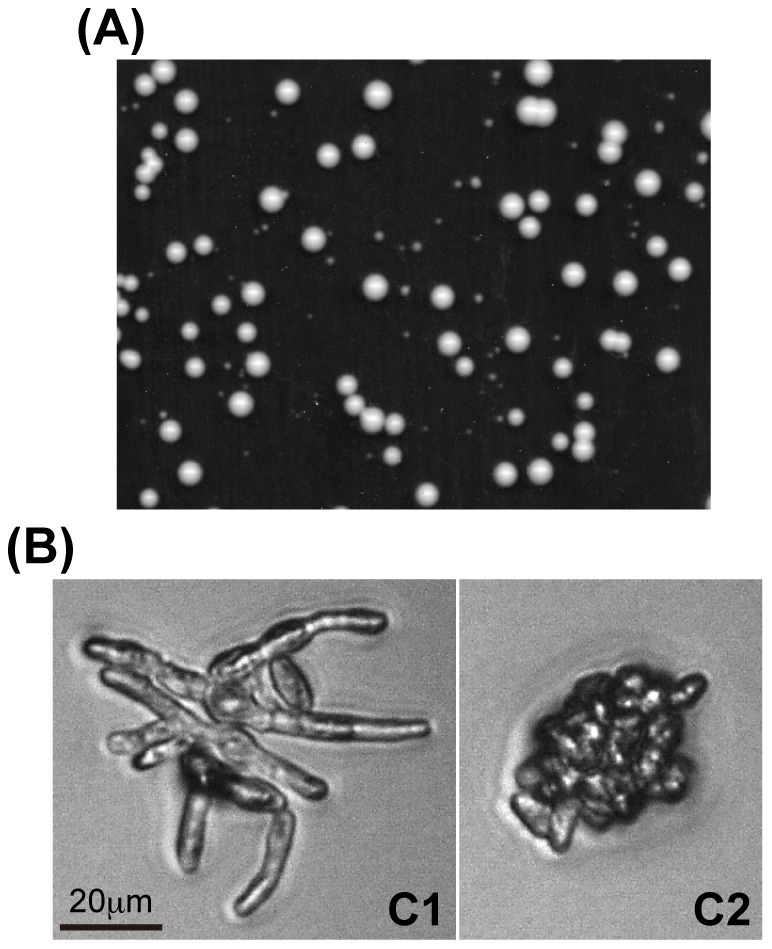
Colony formation from spores produced in triploid fission yeast. (A) Heterogeneously sized colonies. Colonies were incubated on YE medium at 30°C for 5 d. (B) Representative morphologies of C1- (left) and C2- (right) type microcolonies. Colonies were incubated on YE medium at 30°C for 48 h.

When spores produced in triploid cells were incubated for several days, both large and small colonies formed (Figure 1A) [8]. The euploid (haploid or diploid) spores formed large colonies, and aneuploid spores formed small colonies, although the small colonies actually contained mostly euploid cells. Except for the chromosome 3 disome, aneuploids formed visible colonies only after losing aneuploidy at an early stage of colony formation. We speculated that mutations that impair the viability of aneuploid cells would produce a lower number of small colonies. For screening, we prepared spores produced from triploid meioses in each of the deletion mutants (see Materials and Methods) and determined the small to large colony ratio (S/L). In the wild-type background, the ratio was 0.91±0.11 (n = 10), similar to previous results [8].

Of 1659 deletion mutants examined (Table S1), 124 mutants had an S/L<0.5, our cut-off for the study. We repeated the test for 70 mutants arbitrarily selected from among the 124 candidates, and found 41 mutants with a mean S/L<0.5 for both cultures.

#### gtub-mad2 method

The *gtb1-93 mad2Δ* double mutant has a high level of chromosome instability and its colonies contain many aneuploid and dead cells [17]. We therefore speculated that mutations with a reduced ability to sustain aneuploid viability might have synergistic deleterious effects when combined with the double mutant. For screening, we marked the *gtb1* mutation with the hygromycin B-resistance gene (*hph*) and the *mad2* deletion mutation with the nourseothricin-resistance *nat* gene (see Materials and Methods). We crossed this double mutant (strain YT708) with individual deletion mutants constructed with the G-418-resistance gene (*kan*). We first selected mutants that produced a reduced number of triple drug-resistant recombinant colonies compared with the wild-type, and obtained 336 candidates from among 1885 deletion mutants. We then isolated the triple drug-resistant recombinants on a plate containing a low concentration of thiabendazole (TBZ, an inhibitor of microtubule polymerization), and tested whether the triple mutants showed reduced colony formation compared to the parent YT708 in the absence of TBZ. TBZ was used because it partially suppresses the *gtb1-93* mutation [17]. We then isolated the corresponding *hph kan* and *nat kan* double mutants, and examined whether the double mutants could grow similarly to the single mutants on a TBZ-free plate to verify whether the poor viability was due to the triple mutation or to aneuploidy. Among the 336 mutants, 188 were tested, and only 12 had an “aneuploid-specific” synergistic effect. Of these 12, a triploid meiosis test showed that 6 had an S/L<0.5 and 3 had an S/L>0.5, while the remaining 3 were not tested due to poor sporulation. This indicates that a large number of mutants showing synergistic effects in the gtub-mad2 screening had poor spore viability in the triploid meiosis test.

### Components of the CCR4-NOT complex might be involved in aneuploid viability

**Figure 2:**
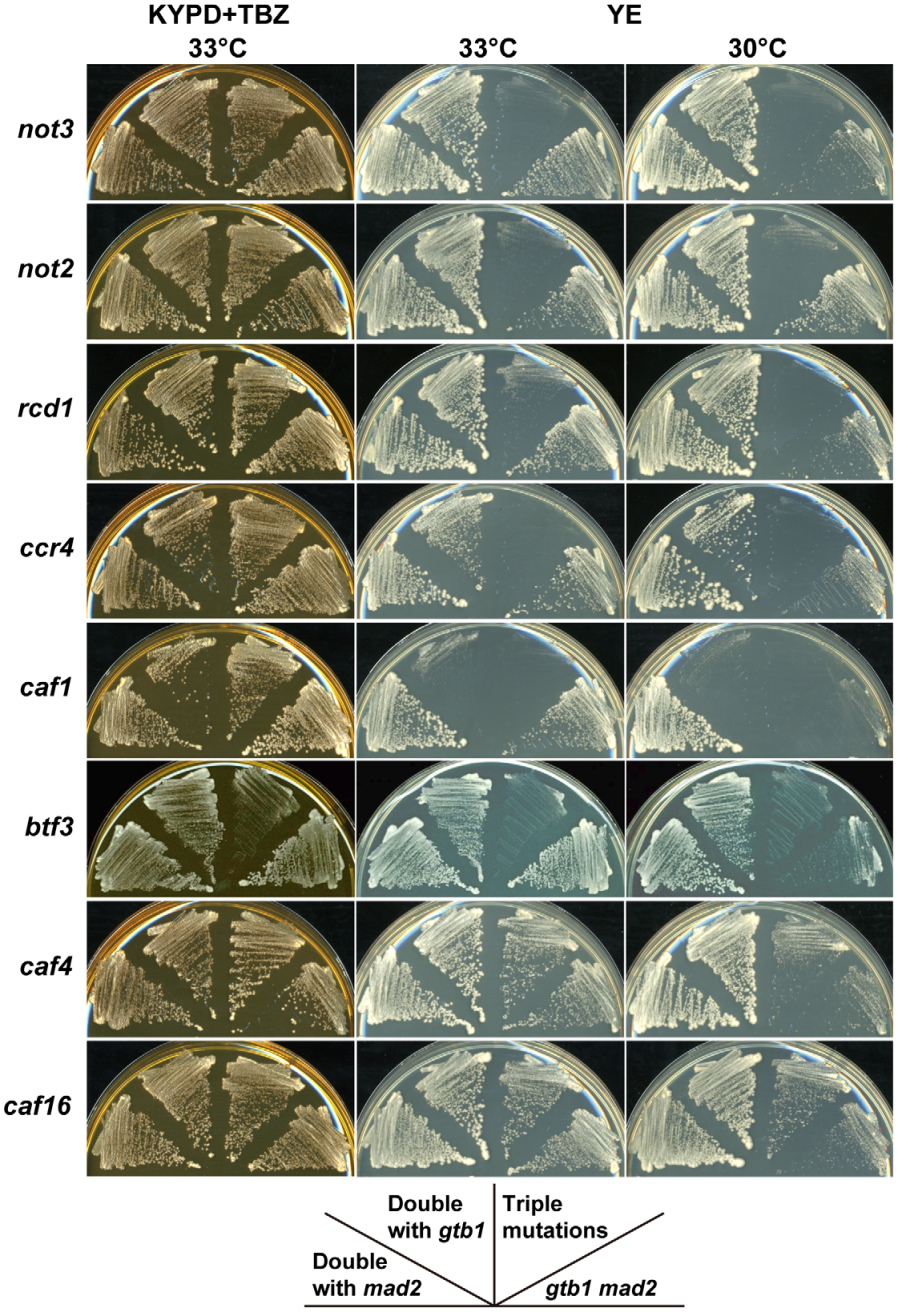
Synergistic effects of CCR4-NOT mutants on the *gtb1 mad2* double mutant. The KYPD+TBZ plate represents a permissive condition for the *gtb1 mad2* double mutant. Chromosome instability of the double mutant was enhanced on the YE plate, particularly at lower temperature. The indicated double and triple mutants were streaked on the plates and incubated at 30°C or 33°C for 3 d.

As indicated above, six mutants were selected from both screening methods. Three of the genes, *not3*, *not2*, and *btf3*, were orthologous to *NOT3* (or *NOT5*), *NOT2*, and *BTT1* in *S. cerevisiae*, respectively (http://old.genedb.org/genedb/pombe/) (see Figure 2 for the gtub-mad2 phenotype). These genes are components of the CCR4-NOT complex, which is a general transcription regulator [18]–[21]. The other three mutants were *swi6* (chromodomain heterochromatin protein), *clp1* (Cdc14-related protein phosphatase), and *SPAC1B1.04c* (predicted to be an ortholog of *PAN3*, a subunit of the poly(A)-specific ribonuclease complex) (Figure S1 and data not shown). In addition, another defective mutant in the poly(A) nuclease (PAN) complex, *ppk26*, had a similar effect on aneuploid viability: a low S/L = 0.52 and a weak but significant synergy with the *gtb1 mad2* mutant (see Figure S1). We did not study these four mutants further.

The deletion collection contained five more mutants defective in CCR4-NOT complex components. Two of these, *caf4* (*CAF4/MDV1* in *S. cerevisiae*) and *caf16* (*CAF16*), were indistinguishable from the wild-type, although the *caf16* mutant had a mild effect on aneuploid viability in a subsequent study, as described below (Table 1). The three other deletion mutants, *ccr4* (*CCR4*), *caf1* (*POP2*), and *rcd1* (*CAF40*), had synergistic effects with the *gtb1 mad2* double mutant, but the *ccr4* and, in particular, *caf1* mutations, also had synergistic effects with the *gtb1* mutation alone (Figure 2), suggesting that their effects are not specific to aneuploidy. These three mutants were not tested in the triploid meiosis screening because they generated unhealthy spores (Table S1), but subsequent examination showed that the aneuploid spores with the *rcd1* mutant had poor viability in the triploid meiosis test, as described below (see Table 1).

**Table 1:** Growth profiles of spores from triploid meiosis.

	Types of spores with indicated microcolony morphology (%)(a)									
	A	C1		C2		others		D	E	
genotype	CF(b)	CF(c)	CNF(b)	CF	CNF	CF	CNF	CNF	CNF	Number of spores tested
Wild type	14.3	13.1 (44.0)	16.7	6.7	0.4	1.6	1.1	22.3	23.8	551
*not3*	18.1	5.5 (26.1)	15.6	5.0	2.0	2.7	1.5	29.7	20.0	744
*not2*	16.9	4.6 (22.5)	15.8	3.3	3.1	2.9	2.4	31.0	20.0	549
*rcd1*	12.4	3.2 (18.3)	14.3	0.0	1.1	1.4	0.7	26.3	40.7	720
*btf3*	12.6	4.5 (28.5)	11.3	4.1	0.6	2.5	1.8	31.1	31.5	682
*caf4*	15.4	10.1 (50.0)	10.1	6.3	0.6	3.4	1.8	30.0	22.4	671
*caf16*	15.0	6.3 (27.0)	17.0	6.1	0.5	1.4	0.4	20.5	32.9	560

(a) For the morphology of C1 and C2 type microcolonies, see Figure 1B.

(b) CF: colony-forming; CNF: colony-not-forming.

(c) Numbers in parentheses indicate percent of colony-forming C1-type microcolonies.

We examined the mutants of the CCR4-NOT complex more closely. Individual spores from triploid meiosis were randomly separated using a micromanipulator and their growth profile was microscopically observed (Materials and Methods). Microcolony morphology was recorded 2 days after spore separation, and the formation of visible colonies was scored 3 to 4 d later (Table 1). We classified microcolonies/cells into six types [8]: type A, large microcolonies comprising normally-shaped cells; C1, microcolonies containing elongated cells with or without septa; C2, microcolonies mainly comprising short and aggregated cells (see Figure 1B for representative C1 and C2 types); D, one germinated cell or two apparently dead cells; E, no apparent germ tube formation or little morphologic change from spores; and others, microcolonies with fewer cells than those in A-type microcolonies, but different qualities from those in types C1 and C2. Types D and E cells in most cases did not divide or showed limited division after day 2. Previous tetrad analyses revealed that A-type microcolonies are produced from haploid or diploid spores, and C1 and C2-types from aneuploid spores [8]. The chromosome 3 disome (n+1) spores made up the C2-type ([8] and present study). As for the D and E types, many of them probably represented aneuploid cells, but some of them were likely euploid cells [8]. Therefore, to evaluate aneuploid viability, we focused on the C1 and C2 types.

As shown in Table 1, the frequencies of A-type microcolonies among the mutants did not significantly differ from wild-type (p<0.05), indicating that the mutations did not significantly affect the viability of euploid spores. In contrast, formation of visible colonies from C1-type microcolonies was reduced in the mutants with the exception of *caf4*. Results from other genetic studies indicate that at least two and possibly five types of aneuploids produce C1-type microcolonies ([8] and O. Niwa, unpublished results). It was not clear whether the CCR4-NOT mutations differentially affected aneuploid types.

**Figure 3:**
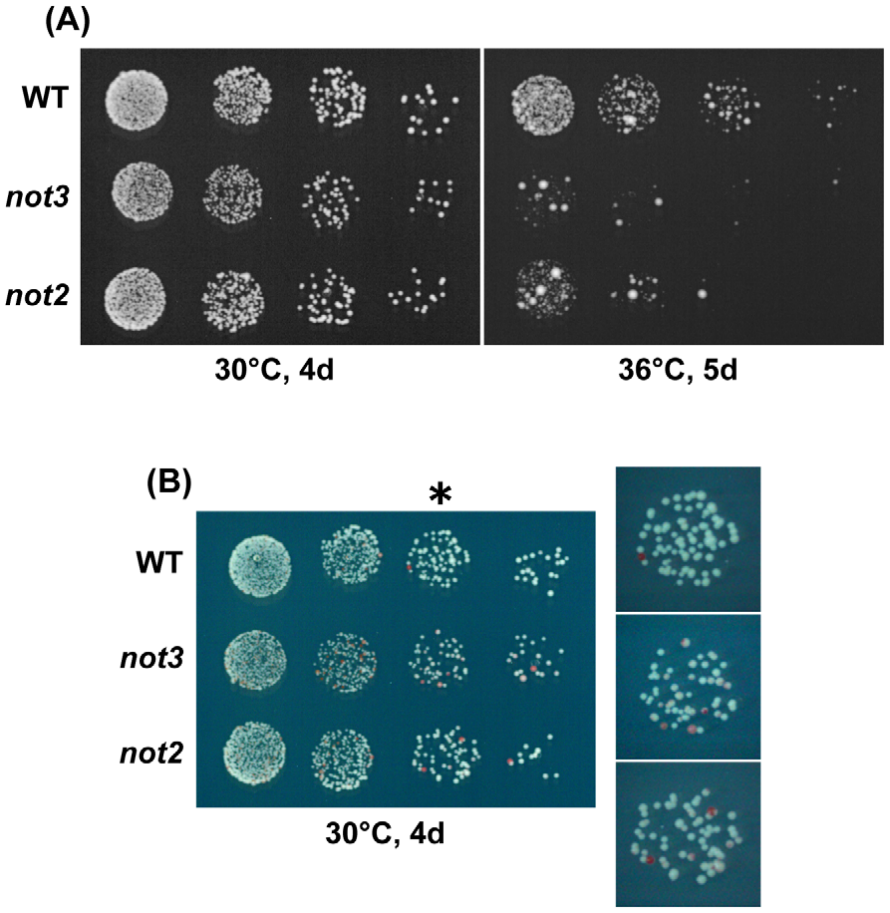
Characterization of chromosome 3 disomes of the CCR4-NOT mutants. (A) Temperature sensitivity of chromosome 3 disomes. 5-fold serial dilutions of the disomes with the indicated mutations were spotted on a selective EMM medium (-adenine) at 30°C for 4 d or at 36°C for 5 d. For all strains, the target cell number for the last dilution was 25. All or most of the large colonies forming after incubation at 36°C were diploid. (B) Stability of chromosome 3 disomes. The indicated strains were spotted on EMM with a low concentration of adenine. Colonies were incubated at 30°C for 4 d. Red colonies represent haploids that lost one copy of chromosome 3. Enlarged images of the third spots (*) on the right show the presence of sectored colonies in the mutants.

Colony formation from C2-type microcolonies appeared to be reduced in the *not3*, *not2*, and particularly in the *rcd1* mutants, while only slightly in the *btf3* mutant. Our previous genetic study suggested that at least the majority of “C2-type” spores from triploid meiosis are chromosome 3 disomes, suggesting that the growth of the chromosome 3 disome is affected in these mutants. To directly address this possibility, we examined the viability of this aneuploid by crossing a “wild-type” disomic strain with a haploid strain carrying one of these mutations. For the *not2* mutant, because the locus is mapped on chromosome 3, we used a different disomic strain, whose *not2* locus was heterozygous with *not2Δ* and *not2* ^+^ (see Materials and Methods). With the exception of *rcd1*, there was little bias against the mutations in meiotic segregants, indicating that these mutations did not appreciably affect the colony-forming efficiency of the disome (Table 2). Thus, it was not clear why the colony-forming efficiency of “C2-type” spores was reduced in some of the CCR4-NOT mutants in the triploid meiosis test. Comparison of the colony size of mutant disomes with that of the wild-type disome, however, revealed that *not3* and *not2* produced much smaller colonies on a selective plate when incubated at 36°C (Figure 3A), a temperature at which the growth of “wild-type” aneuploids was retarded (Figure 3A and Y. Tange and O. Niwa, unpublished results). The temperature sensitivity was more pronounced in the *not3* mutant, so that colonies were barely visible even after prolonged incubation at 36°C. Chromosome 3 disome with *not2*/+ heterozygosity did not show the growth defect, indicating that the *not2* deletion mutation was recessive to the wild-type with respect to the temperature-sensitive growth phenotype (data not shown), and that the presence of the G418-resistance gene did not interfere with aneuploid growth. We also noted that the mutant disomes were less stable so that haploid colonies tended to emerge at increased frequencies (see Figure 3B). Similarly, we compared the disomes of *caf4* and *caf16* mutants with the wild-type. No differences were noted in the colony-forming efficiency or the chromosome stability of these mutant disomes (data not shown). The *not3* and *not2* mutants showed no temperature sensitivity when they were haploid or diploid (see Figure 3A and Figure S2). These findings indicated that deficiency of *not3* and *not2*, but not *caf4* and *caf16*, affected the growth of the chromosome 3 disome, and indicated that the effects of the *not3* and *not2* mutants were not limited to the C1-type aneuploids. In addition, these data suggest that, among the CCR4-NOT genes investigated in this study, the *rcd1* gene has the most important role in aneuploid viability.

**Table 2:** Segregation analysis of chromosome 3 disome.

Relevant genotype of strains crossed with P219 or 56-1 (a)	Number of relevant mutant per number of Ade^+^ segregants (b)
Wild type	0/50 (c)
*not3::kan*	33/50
*not2::kan*	15/24 (d)
*rcd1::kan*	32/50 (e)
*btf3::kan*	22/50
*rad32::nat*	0/20
*rhp51::kan*	0/50
*rhp55::kan*	1/50
*eme1::kan*	0/50
*crb2::kan*	6/50
*rad3::kan*	25/50

(a) P219 (*h* ^−^ *leu1 ade6-M210/ade6-M216*) was crossed with a haploid strain that was *h* ^+^ with *ade6-M216* (or *ade6-M210*) and one of the indicated alleles (except *not2*, which is mapped on chromosome 3). Strain 56-1 (*h* ^−^ *leu1 ade6-M210 not2::kan/ade6-M216 not2* ^+^) was crossed with *h* ^+^ *ade6-M216 not2::kan*. Ade^+^ segregants were selected on an EMM2 plate at 30°C.

(b) Ade^+^ colonies were randomly selected and tested for drug resistance. For the *not2* mutant, see (d).

(c) All tested 12 Ade^+^ segregants had the “unstable Ade^+^” phenotype, indicating a chromosome 3 disome. Note, wild-type did not produce drug-resistant segregants.

(d) For this cross, 24 of 26 Ade^+^ (disomic for chromosome 3) were G-418 resistant. Of these 24 segregants, 15 were homozygous for the *not2::kan* mutant, while 9 were heterozygous (see Materials and methods).

(e) G-418 resistant Ade^+^ segregants in this mutant were generally small, and upon restreaking on YE plates only stable Ade^+^ colonies (probably diploids) and Ade^−^ haploid colonies were produced. Chromosome 3 disome was hardly recovered thereafter.

### Growth retardation of aneuploid cells in the *not3* mutant

**Figure 4:**
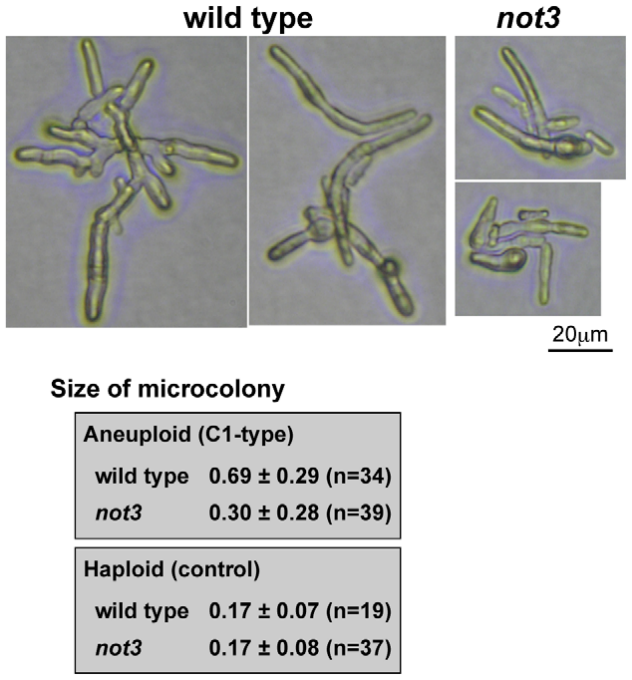
Comparison of C1-type microcolony size in wild-type and *not3* mutant. Pictures are representative images of C1 type microcolonies from aneuploid spores. The difference was statistically significant for aneuploid spores (Mann-Whitney U-test; p = 4×10^−7^), while for the control haploid spores there was no significant difference (p = 0.41). The size estimation procedure is described in the Materials and Methods.

To examine how the *not3* mutation affects the growth of aneuploid cells, we compared the sizes of C1-type microcolonies. Photographs of the microcolonies were obtained after incubation for 52 h at 30°C. The mean area of C1-type microcolonies in the wild-type was approximately two times that of the *not3* mutant (Figure 4). This was not due to a higher incidence of microcolonies containing euploid cells, because microcolonies containing euploid cells were not included in this analysis. The sizes of individual cells in the mutant microcolonies appeared to be smaller than the wild-type. In a control study using haploid spores, the size of the microcolonies after 16 h incubation was indistinguishable between mutant and wild-type. These findings suggest that the wild-type *not3* gene is required to maintain growth of at least some types of aneuploid cells.

### Effect of CCR4-NOT mutations on the genome-wide gene expression profile in fission yeast

The primary function of the CCR4-NOT complex is thought to be the general regulation of mRNA levels for a wide range of genes. Accordingly, genome-wide gene expression analyses in *S. cerevisiae* revealed that some observed genes were either overexpressed or underexpressed by at least 2-fold in deletion mutants of the CCR4-NOT genes, although there are some inconsistencies between studies in the observed frequencies of the affected genes in each of the mutants [21], [22].

We introduced a *not3*, *not2*, or *caf4* deletion mutation in a wild-type haploid fission yeast, and whole genome microarray analysis was performed for these mutants as well as for the parental wild-type strain to determine the effect of each mutation on the overall gene expression pattern in exponentially-growing cells. The expression profile of individual genes in a mutant was presented as the ratio to wild-type (see Materials and Methods). The number of genes with effective values for *not3*, *not2* and *caf4* mutants was 4940, 4926, and 4928, respectively. Among these, the number of genes whose expression was affected by at least 1.5-fold (p<0.05) was 141 (2.9%), 61 (1.2%), and 17 (0.3%), respectively (Table S2). Of these, 30 genes were affected in both *not3* and *not2*, 10 genes in both *not3* and *caf4*, and 4 genes in both *not2* and *caf4*. Among the genes affected in both *not3* and *not2*, 27 of 30 were either overexpressed or underexpressed in both of the mutants, suggesting that Not3 and Not2 components of the CCR4-NOT complex function in the same direction in the regulation of a subset of genes. There are, however, exceptions to this rule. The expression of *urg1* (urg for uracil regulatable gene [23]) and *urg2* (and, to a lesser degree, *urg3* [data not shown]) was reduced in the *not3* mutant, but increased in the *not2* and *caf4* mutants (Table S2). The expression profiles of *SPAPB24D3.07c* was opposite those of the *urg* genes (increased in *not3* and decreased in *not2*).

Another feature of the gene expression profile was that many of the genes that were underexpressed in *not3* and *not2* mutants (Table S2) mapped within two subtelomeric regions of chromosome 2; one is a 110-kb region centered 120 kb from the left terminus and the other is a 70-kb region 90 kb from the right terminus. Several genes were in these regions in the *not3* (28 genes) and *not2* (16 genes) mutants, accounting for 36% and 41%, respectively, of the genes listed as underexpressed in Table S2. It should be noted that most of the genes that mapped to these regions but are not listed in Table S2 also tended to be underexpressed in these mutants (data not shown), suggesting that Not3 and Not2 are involved in the regional control of gene expression. Although we do not understand how these microarray results are relevant to aneuploid phenotypes, the numbers of genes affected in the *not3*, *not2*, and *caf4* mutants roughly correlated with the severity of aneuploid phenotypes, such as the temperature-sensitivity of the chromosome 3 disome as well as the poor colony-forming efficiency of aneuploid spores (see Figure 3 and Table 1). Altered expression of some specific genes might be also relevant to aneuploid viability (see Discussion).

### The CCR4-NOT complex may be also required for aneuploid viability in budding yeast

Because the CCR4-NOT complex is evolutionarily conserved, we examined whether deficiency of the complex in *S. cerevisiae* also affects aneuploid viability. We made triploid strains with *not3* or *caf4* deletion mutations in otherwise similar genetic backgrounds and tested the viability of the resulting spores (Materials and Methods). As a control, we separated spores produced in wild-type triploids either by tetrad dissection or by random spore analysis and scored the number of spores that formed visible colonies after incubation for 6 d at 30°C. Note that due to the large number of chromosomes in this yeast (n = 16), virtually all the spores were aneuploid. With a few exceptions, only aneuploids with fewer than six extra chromosomes are tolerated in this yeast [11]–[13], which comprise about 10% of the total spores produced in triploid yeast. As summarized in Table 3, the overall colony-formation rate was 54.3% (n = 1483), with only a slight difference between the two different triploid strains. This value is greater than those previously reported (18% in [12]) and 38.5% in [24]), which may be due to genetic variations among laboratory yeast strains. These colony-forming efficiency values indicate that a significant portion of aneuploid spores survive and produce colonies, most probably by reducing the number of extra chromosomes during cell proliferation. Experimental spores from the mutant triploids had reduced colony-forming efficiencies of around 34% (n = 987) and 36% (n = 960) for the *not3Δ* and *caf4Δ* mutants, respectively (p<0.01 for both). Because the mutants did not appreciably affect the viability of haploid spores (Table 3), the reduced viability of spores from triploid meioses suggests that these two genes have important roles in sustaining aneuploid viability.

**Table 3:** Viability of spores produced in triploid cells in *S. cerevisiae*.

		Spore viability		
ploidy	Relevant genotype	Visible colony formed (%)	Total number of spores tested	Method
triploid	α/**a**/**a** wild type	182 (60.7)	300	Tetrad dissection
	α/α/**a** wild type	288 (52.9)	544	Tetrad dissection
		335 (52.4)	639	Random spores
	α/α/**a** *not3Δ*	103 (36.3)	284	Tetrad dissection
		222 (31.6)	703	Random spores
	α/α/**a** *caf4Δ*	124 (38.8)	320	Tetrad dissection
		206 (32.2)	640	Random spores
diploid	α/**a** wild type	57 (95.0)	60	Tetrad dissection
	α/**a** *not3Δ*	58 (92.1)	63	Tetrad dissection
	α/**a** *caf4Δ*	60 (93.8)	64	Tetrad dissection

**Figure 5:**
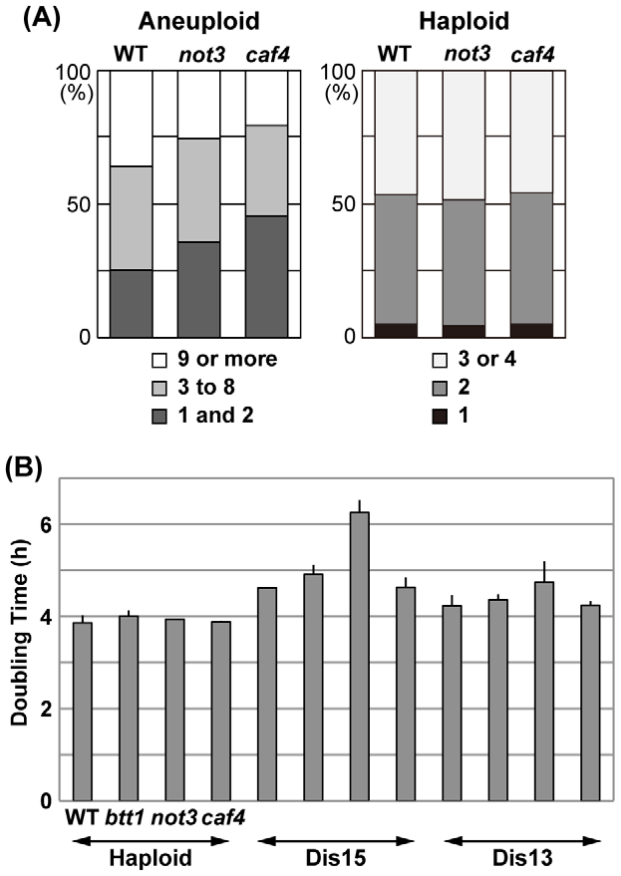
Effects of CCR4-NOT mutants on the proliferation of aneuploid cells in *S. cerevisiae*. (A) Effect of *not3* and *caf4* mutants on aneuploid spores. Randomly selected spores prepared from triploid meioses were individually plated on YPD plates and incubated at 30°C for 15.5 h. The number of cells in each microcolony was counted. A cell with an emerging bud whose diameter was smaller than approximately two-thirds that of the mother cell was counted as one. For haploid spores (wild-type: n = 226; *not3*: n = 216; *caf4*: n = 210), the number of cells was counted after 4.5 h of incubation. (B) Doubling times of *S. cerevisiae* haploid and disomes. Disomes having an extra chromosome (XV or XIII) with the indicated mutations were used. Colonies were incubated in a synthetic SD medium selective for disomy (−His+G418) at 22°C. Culture densities were measured every 2 h. Doubling time was calculated from an exponentially growing phase of each culture (OD_600_ values, approximately from 0.15 to 1.0). Data are shown as mean ± SD (n = 3).

In a separate experiment, we counted the number of cells in each microcolony grown from individual spores after incubation for 15.5 h (Figure 5A), and scored the number of visible colonies after 6 d of incubation. As summarized in Figure 5A, the number of spores that remained single cells or divided only once was significantly increased in both mutants compared to wild-type (p<0.01): 25.3% (wild-type) vs. 35.8% (*not3Δ*) and 45.6% (*caf4Δ*). In contrast, the number of spores that divided many times to produce nine or more cells comprised 35.9% (wild-type), 25.5% (*not3Δ*), and 20.6% (*caf4Δ*), indicating that the proliferation rate of many types of aneuploids was significantly reduced in the mutants (p<0.01). In this experiment, visible colony formation rates were 55.2% (wild-type, n = 384), 39.6% (*not3Δ*, n = 384), and 31.6% (*caf4Δ*, n = 384). For haploid spores, the timing of spore germination and subsequent cell divisions did not significant differ between wild-type and mutants (Figure 5A). Thus, it is likely that these CCR4-NOT mutants decreased the cell proliferation potential of aneuploid cells in *S. cerevisiae*. The *caf4Δ* mutation tended to have a greater effect on spore viability than the *not3Δ* mutation, which contrasts with the results for *S. pombe*, suggesting that the roles of individual components in the CCR4-NOT complex for aneuploid viability differ among these yeasts. More importantly, however, the CCR4-NOT complex appears to have a role in aneuploid viability, suggesting that its function in aneuploidy may be conserved in other eukaryotes.

### Effect of CCR4-NOT gene defects on disomic budding yeast

To address how the CCR4-NOT defects in *S. cerevisiae* impact defined types of aneuploids, we examined the growth rate of several types of disomes that contain *not3*, *caf4,* or *btt1* (*btf3* in *S. pombe*) deletion mutants (see Materials and Methods). Disome XV has a significantly longer doubling time than the wild-type haploid [9]. We found that deficiency of the *NOT3* gene further decreased the growth rate of this type of disome (Figure 5B), which was consistent with the smaller colonies produced by the mutant disome compared with disome XV carrying wild-type *NOT3*. Mild detrimental effects of the *not3* and *btt1* mutants might be seen in disome XIII and disome XV, respectively. As for disomes I and II, we detected no effects of any of the mutants on growth rate (data not shown).

Many types of aneuploids in *S. cerevisiae* are hypersensitive to genotoxic agents, including camptothecin (CPT), phleomycin, and hydroxyurea (HU), and some other types of aneuploids are rather resistant to some drugs such as rapamycin and bleomycin [10], [13]. We examined whether the CCR4-NOT mutations affected the sensitivity of the disomes to CPT, HU, and phleomycin. Based on their colony size, disome I (and disome XIII less clearly) became more resistant and disome II became more sensitive to CPT in the absence of *NOT3* (Figure S3A). Also, disomes II became very weakly resistant to CPT in the *caf4* mutant. In addition, haploids with the *NOT3* defect were slightly sensitive to HU and the sensitivity became more conspicuous in disome I (Figure S3B). With regard to phleomycin, we noted no specific effect of the CCR4-NOT mutants on disomes I, II, XIII, and XV (data not shown). Thus, the CCR4-NOT defects did not have strong effects on the defined types of aneuploids with only one extra chromosome, yet the CCR4-NOT defects did have some specific interactions with the aneuploids.

### Other genes that may affect aneuploid viability

**Figure 6:**
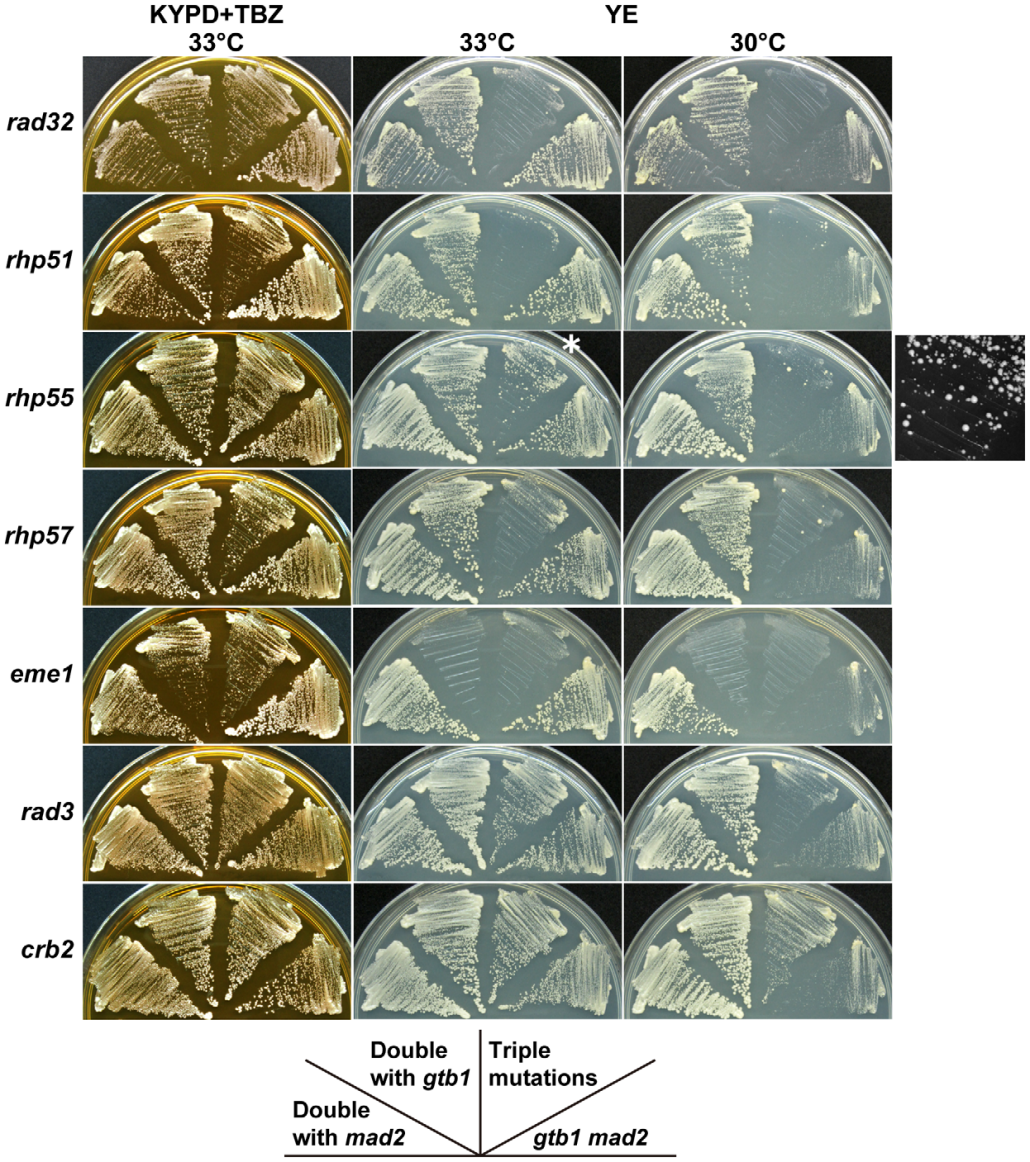
Synergistic effects of DNA repair-related mutants on the *gtb1 mad2* double mutant. See Figure 2 legend for details. *Enlarged black and white image of this portion is shown on the right side.

In the course of the present study, we observed that a *rad32* (a homolog of *MRE11*) mutant was defective in maintaining chromosome 3 disomy (Table 2). It was also synergistic with the *gtb1 mad2* double mutant (Figure 6), suggesting that DNA recombination/repair is involved in aneuploid viability and/or maintenance. We tested whether deletion mutations in recombination/repair-related genes had a synergistic effect with the *gtb1 mad2* double mutant. As shown in Figure 6, *rhp51* (the *RAD51* homolog), *rhp55* (*RAD55*), *rhp57* (*RAD57*), and *eme1* (*MMS4*) showed synergistic interactions with the double mutant. The effect of *rad55* and *rad57* deletions seemed weaker than that of *rad51*, but some ambiguity remains due to the emergence of fast-growing colonies with unknown genetic properties (see Figure 6). All results from this and other repeated tests indicate that these two mutants had synergistic effects with the *gtb1 mad2* double mutant. This finding is consistent with Rhp55 and Rhp57 functioning as a complex to stimulate Rhp51 activity [25]. The Mus81/Eme1 complex is a DNA structure-specific endonuclease that functions in a late stage of homologous recombination repair [26]. Because the *eme1* mutant showed synergistic effects with the *gtb1-93* mutation alone, it is possible that its effect was not specific to aneuploidy. Rad3 and Crb2 are involved in DNA damage checkpoint control [27], but these proteins do not appear to be involved in aneuploid viability, based on the negative results of both the gtub-mad2 assay (Figure 6) and the triploid meiosis test (Table S1). Overall, the results suggest that the core machinery for recombination repair, e.g., Rad32, Rhp51, Rhp55, and Rhp57, are involved in aneuploid viability. Because these gene products are, to a certain extent, required for sporulation, the triploid meiosis test could not be performed adequately. It should be noted that in these mutants, unlike in wild-type cells, the minichromosome Ch16 was not stable ([28] and data not shown). Thus a chromosome-destabilizing effect may explain why triple mutants became sicker than the parental double mutants. It is conceivable that recombination repair is required for aneuploid viability, however, because chromosome-destabilizing mutants did not necessarily have synergistic effects with the *gtb1 mad2* double mutant (see Table S1), and particularly because many types of aneuploid cells have defective DNA damage repair [10]. In the segregation analysis of the chromosome 3 disome, *rad32*, *rhp51*, *rhp55* and *eme1*, no or very few disome segregants were recovered (Table 2). This suggests that these homologous recombination repair genes are required for viability of the disome, maintenance of the extra chromosome, or both.

In addition, we fortuitously found that an mRNA transport mutant, *mex67* (the ortholog of *S. cerevisiae MEX67*) [29], had suppressor activity for the *gtb1 mad2* mutant. That is, the triple mutant produced slightly larger colonies than the parental strain on YE plates, a condition that enhances aneuploid production (Figure S1). We then tested other transport-related mutants by the gtub-mad2 method, including *SPAC14C4.06c* (*S. cerevisiae NAB2*), *nup97* (*NIC96*), *SPAC328.05* (*HRB1/GBP2*), and *crp79* (no ortholog known in other species). Interestingly, *crp79* had a similar effect on the *gtb1 mad2* mutant, although the effect was weaker than that of *mex67*. Crp79 was identified as a multicopy suppressor of the essential transport mutant *rae1* [30]. Among other tested mutants, *SPAC14C4.06c* made the *gtb1 mad2* mutant sicker, while the others had little or no effect.

## Discussion

The two yeasts *S. cerevisiae* and *S. pombe* are in distantly related subgroups of the phylum Ascomycota [31], [32], thus comparisons of these yeasts should provide good insight into the operations of eukaryotic cells. The present results suggest that deletion mutations in at least some components of the CCR4-NOT complex affect the viability of aneuploids in both fission yeast and budding yeast. Genetic as well as biochemical studies in *S. cerevisiae* and other species revealed that the complex is involved in several aspects of mRNA metabolism, including negative and positive regulation of transcription initiation, mRNA elongation, RNA degradation in the nucleus, and deadenylation of the poly(A)-tail for mRNA decay, with its primary function being the regulation of mRNA level in response to different environmental conditions [18]–[20], [33]. In addition, Not4 has ubiquitin ligase activity [34], [35] and the CCR4-NOT complex interacts with a nascent-associated polypeptide complex [35], which suggests a protein metabolism function. In *S. cerevisiae*, a discrete form of the complex, approximately 1 MDa in size and containing 10 subunits, Cdc39 (also known as Not1), Cdc36 (Not2), Not3, Mot2 (Not4), Not5, Ccr4, Pop2 (Caf1), Caf40, Caf130, and Btt1, have been identified [19]–[21]. This “core” complex is associated with other components, including Caf4 and Caf16, probably in a loose manner to form a larger form of the CCR4-NOT complex. The CCR4-NOT complex is thought to be conserved in fission yeast [36], [37], although some structural and functional divergence in its evolution has been proposed [38].

The Ccr4-Pop2(Caf1) moiety of the complex in *S. cerevisiae* has poly(A)-specific deadenylase activity, which together with the PAN complex accounts for the cytoplasmic deadenylase required for mRNA degradation [39]. *S. pombe* Caf1 also has deadenylase activity [37], [40]. Although triploid meiosis data are missing for the *ccr4* and *caf1* mutants, these mutants had a strong synergistic effect in the gtub-mad2 assay (Figure 2). Further, we identified two genes, *SPAC1B1.04c* and *ppk26* (presumed components of the PAN complex in *S. pombe*), whose deficiency was associated with synergistic effects with the *gtb1 mad2* mutant and reduced viability of spores from triploid meioses. Thus, the decrease in cytoplasmic deadenylase activity appeared to be associated with reduced aneuploid viability. The deficiency in deadenylase activity should stabilize the mRNA, resulting in too much functional mRNA, which in turn leads to an increase in protein production that could result in an increased need for protein degradation.

We found that deletion mutants of mRNA export-related factors (Mex67 and Crp79) had opposite effects in the gtub-mad2 test, that is, these mutations partially rescued the poor colony formation of the *gtb1 mad2* double mutant (Figure S1 and Y. Tange and O. Niwa, unpublished results). In these mutants, the amount of functional cytoplasmic mRNA might be decreased, which is opposite to the case in the deadenylase mutants. Our preliminary examination by the triploid meiosis test as well as by the segregation analysis for chromosome 3 disomy, however, indicated that the *mex67* deletion mutation reduced, rather than increased, aneuploidy viability (Y. Tange and O. Niwa, unpublished results). More specifically, the chromosome 3 disome was extremely unstable and the other types of aneuploids had reduced colony-forming efficiency associated with retarded growth. Further studies are needed to understand why there is an apparent rescue of the poor colony growth of the *gtb1 mad2* double mutant by the *mex67* mutant. Nevertheless, it is interesting that a class of putative regulators of mRNA dynamics also probably affects the viability of aneuploids.

How do the other mutations in the CCR4-NOT components, that is, *not3*, *not2*, *rcd1*, and *btf3*, affect aneuploid viability? In the present study, we performed a gene expression analysis of the fission yeast *not3*, *not2*, and *caf4* mutants. The results indicated that the numbers of genes whose expression is strongly affected in the deletion mutants of the CCR4-NOT complex is lower than that in corresponding mutants in *S. cerevisiae* [21], [22]. This finding suggests that components of the CCR4-NOT complex or the complex as a whole in *S. pombe* might have different functions in the gross regulation of mRNA metabolism from those in *S. cerevisiae*, or that fission yeast might have a system against perturbations in mRNA turnover to ensure mRNA homeostasis, at least in haploid cells. Several interesting points may be drawn from our microarray data. Firstly, as already mentioned, the numbers of genes affected in each of the mutants correlated with the severity of aneuploid phenotypes, e.g., the growth defect of the chromosome 3 disome is most severe in the *not3* and least severe in the *caf4* mutant. The larger number of genes affected in the *not3/not2* mutants may be more detrimental to the gene expression imbalance occurs in aneuploid cells. Second, among genes whose expression is affected in the *not3* and *not2* mutants, a number of genes are involved in transport between the cell and its environment. This may be relevant to the fact that fission yeast aneuploids are generally sensitive to environmental changes, including temperature and nutrition ([8] and present study). Third, we observed that a kinetochore protein, CENP-C homolog (Cnp3), is underexpressed by 1.8- and 1.9-fold in the *not3* and *not2* mutants, respectively (Table S2). Fission yeast Cnp3 is required for correct chromosome segregation [41], but because the minichromosome Ch16 is not appreciably destabilized in either of these CCR4-NOT mutants (data not shown), this level of reduction in Cnp3 expression does not seem to interfere with chromosome segregation in the quasi-haploid situation. Also, this reduction may not readily explain the growth retardation observed in a type of aneuploid cells (Figure 4). Provided that chromosome stability is generally reduced in aneuploid yeasts [8], [10], the lower expression of CENP-C may bring about further chromosome destabilization, and thus reduced viability. It remains to be examined whether Cnp3 expression is also reduced in other CCR4-NOT mutants.

Another important point that must be considered is that mutants of the CCR4-NOT complex and its interacting factors are hypersensitive to DNA-damaging agents in both *S. cerevisiae* and *S. pombe* [42]–[45], suggesting that the complex is involved in DNA damage repair and/or checkpoint. In *S. cerevisiae*, *CCR4* and *DHH1* (an RNA helicase interacting with Ccr4/Pop2) are required for resistance to ionizing radiation and other DNA-damaging agents. *POP2*(*CAF1*), *NOT3*, *NOT2*, and some other interacting genes confer radiation hypersensitivity when deleted [43]. In *S. pombe*, *caf1*, *ccr4*, *rcd1*, and *not2* mutants are sensitive to DNA replication stress and/or to an ultraviolet light mimetic agent [44], [45]. Provided that, in both fission and budding yeast, homologous recombination repair function for DNA double strand breaks may be generally impaired in aneuploid cells [10], it is conceivable that the DNA repair function of the CCR4-NOT complex is involved in aneuploid viability. This is consistent with our finding that the genes required for homologous recombination repair had a synergistic effect with a chromosome instability mutant that continuously produced aneuploid cells.

In summary, the present findings demonstrate that the CCR4-NOT complex and other factors involved in the regulation of cellular mRNA level as well as proteins that are required for DNA recombination/repair play a crucial role in determining the fate of aneuploid cells.

## Materials and Methods

### Strains and genetic methods

Culture media used in the study were YE and YPD (rich media), EMM and SD (synthetic media), MEA (for conjugation and sporulation in *S. pombe*), and Sporulation medium (for *S. cerevisiae*) [46], [47]. YE medium was prepared for fission yeast using Bacto Yeast Extract (Becton Dickinson, Franklin Lakes). YES medium contained five supplements (adenine, uracil, leucine, histidine, and lysine) in YE [47]. Phloxine B plates were prepared as described previously [47]. YPD was prepared with Bacto Yeast Extract, Bacto peptone, and dextrose, and used for *S. cerevisiae*. KYPD (K for Kyoto) was analogous to YPD medium, but Polypeptone (394-00115, Nihon Seiyaku, Tokyo) and Yeast Extract (42007000, Oriental-Yeast, Tokyo) were used instead of Bacto Peptone and Bacto Yeast Extract. KYPD was originally used as an optimal medium for the fission yeast aneuploid study, particularly for cultivating the chromosome 3 disome. EMM was another good medium for the chromosome 3 disome, when sodium glutamate (5 g/l) was used as the nitrogen source. NH_4_Cl was a very poor nitrogen source for aneuploid proliferation. KYPD was also used with 5 µg/ml of TBZ as a permissive incubation medium for the *gtb1 mad2* double mutant. Malt Extract Broth was purchased from Oxoid (Basingstoke, UK) for MEA. For SD, Difco Yeast Nitrogen Base (without amino acids or without amino acids and ammonium sulfate) was used (Becton Dickinson).

### Preparation of spores from triploid meiosis

The yeast collection we used in this study was an early version of a deletion library and consisted of 2663 deletion mutants, which covered approximately 73% of non-essential fission yeast genes (3630 genes according to Kim *et al.* [16]). Their genotype was *h* ^+^ *leu1-32 ura4-D18 ade6-M210* (or *M216*) *orfΔ::kanMX4* (most of the open reading frame [ORF] of a gene was disrupted with the G418-resistance gene) [16]. For the triploid meiosis analysis, each strain was crossed with a wild-type *h* ^−^ strain, L972, to isolate *h* ^−^ *orfΔ::kanMX4* and *h* ^+^ *leu1-32 orfΔ::kanMX4* segregants. G-418 (G5013, Sigma-Aldrich Inc, St. Louis, MO) at a concentration equivalent of 100 µg/ml was used for the selection. We failed to obtain the targeted segregants in crosses for 643 deletion mutants.

The *h* ^−^ segregant obtained was then treated with methyl 2-benzimidazole carbamate (MBC; Wako, Osaka) to induce diploidization. Briefly, MBC stock solution (7.5 mg/ml) in dimethyl sulfoxide was added to a logarithmic phase culture in YE medium at 1/300 volume of the medium, followed by incubation at 26°C for 4.5 h. After incubation, we separated the affected cells (elongated cells with swelling or a short protrusion near the middle of the cell) with a micromanipulator on a Phloxine B plate, and incubated them at 26°C to obtain diploid colonies. More than 50% of the separated wild-type cells formed diploid colonies. In some cases, we spread the MBC-treated cell culture directly onto Phloxine B plates and isolated dark-colored colonies as diploid colonies. Stability of the diploid cells was assessed by spreading the cells on Phloxine B plates. For those showing poor stability, we tested the stability of the Ch16 minichromosome. Table S1 provides semi-quantitative data regarding the stability of the diploid and the minichromosome.

The obtained *h* ^−^/*h* ^−^ diploid strain was then crossed with a corresponding *h* ^+^ haploid strain on MEA at 26°C for 2 to 3 d. To isolate spores, the cell mixture on the MEA plate was digested with 0.5% (v/v in water) β-glucuronidase (G7770, Sigma-Aldrich) at 36°C for 3 h or longer. The number of spores was counted microscopically with a counting chamber. Very few vegetative cells escaped from digestion, allowing subsequent experiments to be performed without purifying the spores.

### Observation of cell growth/colony formation from spores

A known number of spores was plated on YE plates and incubated at 30°C for 4 d. The numbers of small and large colonies were manually counted, and their ratio was calculated. In the initial phase of screening, we observed the plates after 2 d incubation, and visible colonies were counted and marked, followed by further incubation for 2 d, when newly appearing small colonies were counted. At this time, the previously marked colonies had generally grown to be “large” colonies. For a detailed analysis of the spores, individual spores were separated with a micromanipulator onto a YE plate. After 2 d incubation at 30°C, the morphology of each cell/microcolony was observed microscopically and classified into six classes according to Niwa *et al.* (2006) [8] (see text and Table 1 and Figure 1), followed by another 3 to 4 d of incubation after which we determined which microcolonies produced visible colonies.

To compare their size, we took photographs of microcolonies after 52 h incubation at 30°C. The photographs were printed with the images of each microcolony, cut out along the edge and weighed to determine their relative sizes. For a control experiment, spores produced by diploids were incubated for 16 h and the size of their microcolonies was determined.

### Screening of deletion mutants that affect the viability of the *gtb1 mad2* double mutant

A *gtb1 mad2* double mutant, YT708 (*h* ^−^), contained the hygromycin B-resistance gene, *hph*, which was inserted 600 bp upstream of the start codon of the *gtb1-93* mutant gene, and the nourseothricin-resistance *nat* gene, which was used to disrupt the *mad2* gene according to the previously described procedure [48], [49]. YT708 was crossed with *h* ^+^ segregants as described above to introduce the G418-resistant deletion mutations to the *gtb1*, *mad2* background. Hygromycin B (H0654, Sigma-Aldrich) and clonNAT (Werner BioAgents, Jena, Germany) were used for *hph* and *nat* gene selection at 50 µg/ml and 100 µg/ml, respectively, together with G418 to select triple-drug resistant recombinants on YES plates with or without 5 µg/ml TBZ (T8904, Sigma-Aldrich) at 33°C. In an initial screening, we selected deletion mutants that produced a reduced number of triple-drug resistant recombinant colonies on the TBZ-free YES plate, compared with wild-type. The *rad32* deletion we used in this study was not obtained from the deletion library. Instead, it was made separately by replacing the whole ORF with the *nat* gene. We confirmed that all deletion mutants listed in Table 1, Table 2, Figure 6, and Figure S1 had the correct disruption.

### Analysis of disomy in the deletion mutants

Strain P219 was a chromosome 3 disome with the mating type of *h* ^−^. Each chromosome 3 contained the *ade6-M210* and *ade6-M216* alleles. Because the *ade6* mutations complement each other, the Ade^+^ phenotype was used to indicate chromosome 3 disomy. As anticipated based on a previous study [8], the Ade^+^ phenotype was associated with the C2-type microcolony morphology. P219 was crossed with an *h* ^+^ haploid strain carrying a deletion mutation (*kan*) and *ade6-M210* (or *M216*). In the *rad32* mutation, the *nat* resistance gene was used for gene disruption. For the *not2* mutation, we used strain 56-1 (*h* ^−^), which was disomic for chromosome 3 and one of the chromosomes carried the *not2* mutation. Random spores produced from these crossings were plated on EMM plates, followed by incubation at 30°C for 5 d to select for Ade^+^ colonies. Fifty (or 20) colonies were randomly chosen and tested for G-418 (or clonNAT) resistance. From the drug-resistant segregants as well as drug-sensitive segregants, up to 12 colonies were randomly selected and tested for instability of the Ade^+^ phenotype (a genetic characteristic of disomy) by streaking them out on YE plates on which the Ade^+^ (white) and Ade^−^ (red) phenotypes could be discerned based on colony color. For the cross using the *not2* mutation, we first selected Ade^+^ and G-418-resistant colonies. Each of them was streaked on YE plates containing G-418, and we determined whether they were homozygous or heterozygous for the *not2* alleles based on the fact that each of the two *ade6* alleles produce characteristic colony colors (*ade6-M210*; deep red; *M216*: pale red). Therefore, disomes that produced an even mixture of two different red colonies on the G-418 plate were judged to be homozygous for the *not2* deletion allele, while if (almost) all of the Ade^−^ colonies were one of the two red colors, we considered them heterozygous.

### Microarray analysis of the CCR4-NOT mutants in fission yeast

Fission yeast wild-type strain L972 was used as the parental strain. The whole ORF of *not3*, *not2*, or *caf4* gene in the parent was replaced with the G418 resistance gene according to the standard procedure [48]. Gene expression analysis was performed independently twice for each of the mutant and parental strains as described below.

We used the Agilent DNA microarray (15k×8 format; Agilent Technologies, Santa Clara, CA) containing 15,208 probe spots in each array. The 5529 probes representing 5529 fission yeast genes from the *S. pombe* genome sequences [50] (GeneDB:: http://old.genedb.org/genedb/pombe/) were designed using the Agilent eArray platform. Each probe was spotted twice (1484 genes) or three times (4030 genes) to fill 15,058 spots in the array format. Probes for 15 genes selected as replicate probes were spotted 10 times.

PolyA-RNA targets for microarray were prepared as follows. A single colony of *S. pombe* cells on a YES plate was inoculated into YES liquid medium. Cells were incubated at 30°C and collected with filtration when they reached a density of 5×10^6^ cells/ml. Total RNA was isolated by the acid phenol method [51] (http://www.sanger.ac.uk/PostGenomics/S_pombe/). Using the Low Input Quick Amp Labeling Kit, one-color including Cy3-CTP (Agilent Technologies), labeled targets were prepared with 200 ng of total RNA. The labeled targets were purified using an RNeasy Mini Kit (Qiagen Japan, Tokyo). Hybridization and washing were performed under the manufacturer recommended conditions (Agilent Technologies) with 50 ng of labeled targets.

Microarrays were scanned using an Agilent array scanner (G2505C). The fluorescence intensity of each spot was processed using the Feature Extraction software (ver. 10.7.3. as recommended in the manufacturer instructions (Agilent Technologies). All subsequent data processing and analyses were performed with the GeneSpring GX software (ver. 11.5; Agilent Technologies). A coefficient of variation of 50% was used as the cutoff value. Averaged values from the replicates were used to calculate fold-changes in the gene expression in the mutants compared with wild-type. Genes whose expression was changed by at least 1.5-fold are listed (Table S2: unpaired T-test, P<0.05). The sequences of the probes and original data from the microarray experiments were submitted to GEO (http://www.ncbi.nlm.nih.gov/geo/index.cgi; accession number GSE36454).

### Construction of triploid strains in *S. cerevisiae*

Four isogenic derivatives of SK1 [52] were kindly provided by A. Shinohara (Osaka University): *MATα HO:::LYS2 lys2 ura3 leu2 trp1* (HM785), *MAT* ***a*** *HO:::LYS2 lys2 ura3 leu2 trp1* (HM786), *MAT* ***a*** *HO:::LYS2 lys2 ura3 leu2 his4B-LEU2 arg4-nsp* (HM787), and *MATα HO:::LYS2 lys2 ura3 leu2 his4X-LEU2-URA3 arg4-bgl* (HM788). Diploid cells (nonmater) from conjugation between HM785 and HM786 were lightly irradiated with ultraviolet light and plated onto a rich medium plate. To select a mating-proficient diploid colony, colonies formed from this plate were replica plated onto the lawn of HM787 or HM788 cells placed on an appropriate minimal plate. Triploid cells were simultaneously isolated as Leu^+^ Trp^+^ His^+^ Arg^+^ (Ura^+^) colonies on the minimal plate and incubated overnight on a fresh minimal medium plate without prior purification, followed by inoculation onto the sporulation medium. For random spore analysis, asci were digested with 0.25 mg/ml Zymolyase-100T at 36°C for 2 h. When required, individual spores were separated with a micromanipulator on YPD plates and incubated for the indicated period. Diploid cells for control sporulation were made from HM785 and HM787. To obtain triploid cells with homozygous *not3Δ* or *caf4Δ* mutations, respective genes were disrupted individually in HM785, HM786, and HM787 by replacing the whole ORF with the G418 resistance *kan* gene. The resulting three mutant strains were used to construct triploid cells as described above.

### Disomic strains in *S. cerevisiae*

Media used for disomic *S. cerevisiae* were as follows. SD (−His+G418) was a selective medium for all disomic strains (Table S3). Sodium glutamate (1%) was used as a nitrogen source. G418 was added to a final concentration of 200 µg/ml. All disomic strains and control strains were kindly constructed and provided as frozen stocks by J. Sheltzer (Massachusetts Institute of Technology, Cambridge, MA). Disomic strains were tested by CGH [9] to confirm correct whole chromosome disomy immediately before freezing the cultures. Frozen cells were inoculated and incubated on the selective plates at 26°C overnight. The resulting patches were scraped to inoculate liquid selective medium, followed by incubation at 22°C with vigorous shaking. To determine the doubling time of each strain, the OD_600_ was measured every 2 h and values between 0.15 and 1.0 were used to indicate an exponentially growing culture. To minimize possible overgrowth of an unwanted fast-growing cell population in the culture, the OD measurement was started within 24 h from the inoculation for liquid culture, with one interim dilution in fresh medium. At the end of the OD measurement, cultures were spotted on selective plates to ensure that the culture did not contain an abnormal number of fast-growing cells compared with the original frozen stock. Phleomycin (ant-ph-1, InvivoGen, San Diego, CA), camptothecin (208925, Calbiochem, Darmstadt, Germany), and hydroxyurea (H8627, Sigma-Aldrich) were added to YPD medium at the indicated concentrations for the test.

## Supporting Information

Figure S1 Synergistic effects of the indicated mutants on the *gtb1 mad2* double mutant. See Figure 2 legend for details.
(TIF)

Figure S2 Temperature effect on haploid strains with *not3* and *not2* deletion mutations. Serial dilutions were spotted as in Figure 3 and incubated at 30°C or at 36°C for 3 d.
(EPS)

Figure S3 Drug sensitivity of *S. cerevisiae* disomes. (A) Sensitivity of *S. cerevisiae* disome I (Dis1), II (Dis2), or XIII (Dis13) with indicated CCR4-NOT mutations to CPT. YPD plates with the indicated concentration of CPT were incubated at 30°C for the indicated periods. As a control, haploid strains were also tested. (B) HU-sensitivity of Dis1.
(EPS)

Table S1 Effect of the deletion mutant on aneuploid cells. The triploid meiosis shows the S/L ratio (see text for details). The gtub mad2 column indicates whether the gene mutation had a synergistic toxic effect on the gtb1 mad2 double mutant. n: no or little effect; y: appreciable effect. When applicable, the second result was obtained using a different assessment method (see text). The diploid column indicates diploid stability. “1” indicates that this diploid makes a small and deep red colony on the Phloxine B-plate; “2” indicates that the colony size is heterogeneous, especially in the diploid colony; “3” indicates that the haploid makes a small colony; “4” indicates that the colony size is heterogeneous in the haploid colony; “5“ indicates the colony size and color are heterogeneous in the diploid colony; and “6” indicates that the color varies in the diploid colony. ± and −/+ indicate weak and weaker phenotypes, respectively. Cells that have no value indicate the diploid is stable. For those mutants, only the gtub mad2 test was performed, and the diploid stability test was not. The last column shows the instability of the Ch16 minichromosome. ++: highly unstable (∼50% colonies were Ade^−^); +: unstable (about 10%); ±: mildly unstable (1–2%); −/+: slightly unstable (less than 1%); and n: stable (no Ade^−^ found).
(DOC)

Table S2 Microarray analysis of gene expression in the CCR4-NOT mutants. Genes whose expression changed by at least 1.5-fold in the CCR4-NOT mutants (*not3*, *not2*, and *caf4*). In the two columns next to the fold-change column in each mutant section, a: gene expression was increased at least 1.5-fold in the indicated mutant; b: gene expression decreased at least 1.5-fold; c: gene expression was changed but in the opposite direction. *Genes underexpressed in *not3* and/or *not2* mapped within a region near the left terminus of chromosome 2; **mapped near the right terminus (see text for details).
(XLSX)

Table S3 *S. cerevisiae* disomes and control haploid strains used in the present study.
(XLSX)
