# Supplementary figures and images for: The CCR4-NOT Complex Is Implicated in the Viability of Aneuploid Yeasts
Source: PLoS Genet. 2012 Jun 21;8(6):e1002776. doi: 10.1371/journal.pgen.1002776 (PMC3380822; doi:10.1371/journal.pgen.1002776)

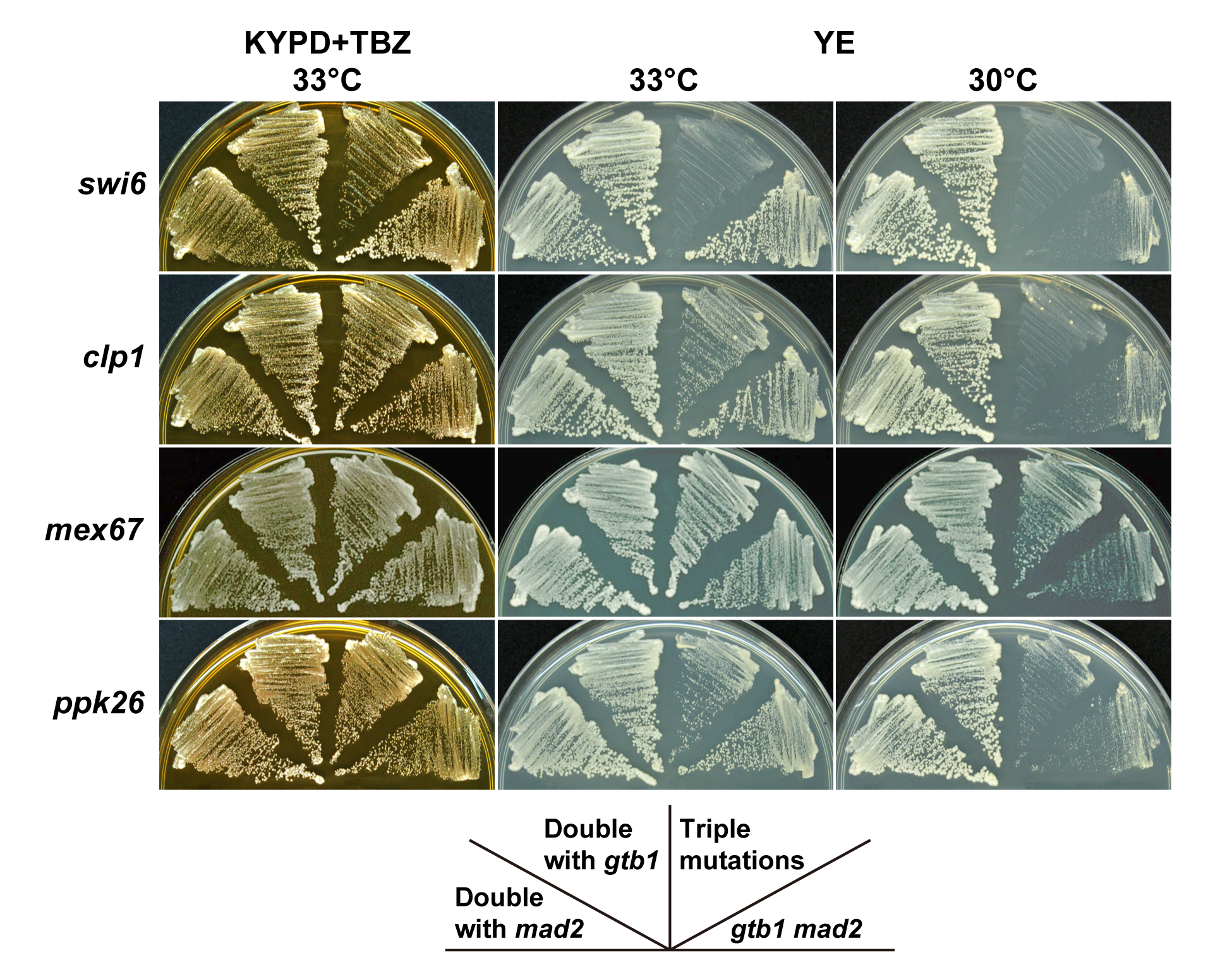

Supplement: Figure S1 — Synergistic effects of the indicated mutants on the gtb1 mad2 double mutant. See Figure 2 legend for details. (TIF) [file pgen.1002776.s001.tif]
