# Supplementary material for: The CCR4-NOT Complex Is Implicated in the Viability of Aneuploid Yeasts
Source: PLoS Genet. 2012 Jun 21;8(6):e1002776. doi: 10.1371/journal.pgen.1002776 (PMC3380822; doi:10.1371/journal.pgen.1002776)
Supplement: Table S1 — Effect of the deletion mutant on aneuploid cells. The triploid meiosis shows the S/L ratio (see text for details). The gtub mad2 column indicates whether the gene mutation had a synergistic toxic effect on the gtb1 mad2 double mutant. n: no or little effect; y: appreciable effect. When applicable, the second result was obtained using a different assessment method (see text). The diploid column indicates diploid stability. “1” indicates that this diploid makes a small and deep red colony on the Phloxine B-plate; “2” indicates that the colony size is heterogeneous, especially in the diploid colony; “3” indicates that the haploid makes a small colony; “4” indicates that the colony size is heterogeneous in the haploid colony; “5“ indicates the colony size and color are heterogeneous in the diploid colony; and “6” indicates that the color varies in the diploid colony. ± and −/+ indicate weak and weaker phenotypes, respectively. Cells that have no value indicate the diploid is stable. For those mutants, only the gtub mad2 test was performed, and the diploid stability test was not. The last column shows the instability of the Ch16 minichromosome. ++: highly unstable (∼50% colonies were Ade−); +: unstable (about 10%); ±: mildly unstable (1–2%); −/+: slightly unstable (less than 1%); and n: stable (no Ade− found). (DOC) [file pgen.1002776.s004.doc]

| **Gene ID (gene name)** | **triploid meiosis** | **gtub mad2** | **diploid** | **Ch16** |
| --- | --- | --- | --- | --- |
| SPBC4B4.06(vps25) | 0.08 | n |  |  |
| SPAC30.01c(sec72) | 0.08 | n |  |  |
| SPAC25H1.03(mug66) | 0.08 | y, n |  |  |
| SPCP31B10.02 | 0.11 | n |  |  |
| SPBC19G7.04 | 0.12 | n |  |  |
| SPBPB10D8.01 | 0.14 | n |  |  |
| SPAC10F6.16(mug134) | 0.15 | n |  |  |
| SPAC222.14c | 0.21 | n |  |  |
| SPCC584.11c | 0.21 | y, n |  |  |
| SPAC4F10.18(nup37) | 0.24 | y, n |  |  |
| SPAC328.04 | 0.26 | n |  |  |
| SPAC20H4.09 | 0.26 | n |  |  |
| SPAC22A12.11(dak1) | 0.27 | n |  |  |
| SPBC17F3.01c(rga5) | 0.27 | n |  |  |
| SPAC167.07c | 0.30 | n |  |  |
| SPBC25B2.04c(mtg1) | 0.32 | n | 5± | n |
| SPAC1805.08(dlc1) | 0.33 |  |  |  |
| SPAC27F1.05c | 0.34 | n |  |  |
| SPBC1734.15(rsc4) | 0.35 | n |  |  |
| SPAC19A8.05c(sst4) | 0.35 | n |  |  |
| SPAC186.04c | 0.35 | n |  |  |
| SPBPJ4664.06(gpt1) | 0.35 | y, n |  |  |
| SPAC1420.01c | 0.35 |  |  |  |
| SPCC569.07 | 0.36 | n |  |  |
| SPAC9E9.03(leu2) | 0.37 | y, n |  |  |
| SPBC651.03c(gyp10) | 0.37 | y, n |  |  |
| SPBC1105.04c(cbp1) | 0.40 | n |  |  |
| SPBC577.10 | 0.40 | n, n |  |  |
| SPCC364.06(nap1) | 0.41 | y, n |  |  |
| SPBC25H2.05 | 0.42 | n, n |  |  |
| SPCC4G3.15c(not2) | 0.42 | y, y |  |  |
| SPBC17A3.06 | 0.43 | n |  |  |
| SPAC27E2.04c(mug155) | 0.43 | n |  |  |
| SPAC11E3.04c(ubc13) | 0.43 |  |  |  |
| SPAPB1A10.03(nxt1) | 0.44 | n |  |  |
| SPBC16E9.11c(pub3) | 0.44 | n |  |  |
| SPBC17D1.06(dbp3) | 0.44 | n | 5-/+ | n |
| SPBC691.01 | 0.44 | n, n |  |  |
| SPCC757.09c(rnc1) | 0.44 | y, n |  |  |
| SPAC16E8.13 | 0.44 |  |  |  |
| SPBC19G7.06(mbx1) | 0.45 | y, n |  |  |
| SPBC336.13c | 0.46 | y, n |  |  |
| SPAC17G8.09(shg1) | 0.46 |  |  |  |
| SPBC2G2.02(syj1) | 0.47 | n |  |  |
| SPCPJ732.03(meu15) | 0.47 | n |  |  |
| SPAC458.04c(dil1) | 0.47 |  |  |  |
| SPCPB1C11.02 | 0.48 | n |  |  |
| SPAC6G10.06 | 0.48 | n |  |  |
| SPAC31A2.09c(apm4) | 0.48 | y, n |  |  |
| SPCC16C4.01(sif2) | 0.48 |  |  |  |
| SPAPB1A10.10c(ypt71) | 0.48 |  |  |  |
| SPBC31F10.12 | 0.49 | n |  |  |
| SPAC186.02c | 0.49 |  |  |  |
| SPBC16H5.06 | 0.50 | n |  |  |
| SPBC18E5.11c | 0.50 | n |  |  |
| SPCC553.04 | 0.50 | n |  |  |
| SPAC1142.08 | 0.50 | n |  |  |
| SPBC16H5.12c | 0.50 | y |  |  |
| SPAC1D4.11c | 0.51 | n | 6 | n |
| SPAC824.08 | 0.51 | n |  |  |
| SPAC144.04c | 0.51 | n |  |  |
| SPAC13G7.05 | 0.51 | n |  |  |
| SPBC947.01 | 0.51 | n |  |  |
| SPBP8B7.21 | 0.51 | n |  |  |
| SPAC25B8.01 | 0.51 | n |  |  |
| SPBC30D10.16 | 0.51 | n |  |  |
| SPBP35G2.13c | 0.51 | n |  |  |
| SPAC24C9.12c | 0.51 | n |  |  |
| SPAC11D3.09 | 0.51 |  |  |  |
| SPAC3H1.14 | 0.51 |  |  |  |
| SPAC9G1.12 | 0.52 | n |  |  |
| SPAC25B8.08 | 0.52 | n |  |  |
| SPBC2A9.11c | 0.52 | n |  |  |
| SPCC553.12c | 0.52 | n |  |  |
| SPBC21C3.09c | 0.52 | n |  |  |
| SPAC23G3.08c | 0.52 | n |  |  |
| SPAC212.06c | 0.52 |  |  |  |
| SPCC4B3.01 | 0.52 |  |  |  |
| SPBC1734.08 | 0.53 | n |  |  |
| SPBC2D10.15c | 0.53 | n |  |  |
| SPAC1705.02 | 0.53 | n |  |  |
| SPBC800.09 | 0.53 | n |  |  |
| SPCC1620.04c | 0.53 | n |  |  |
| SPBC23G7.07c | 0.53 | n |  |  |
| SPAC12B10.01c | 0.53 | y, n |  |  |
| SPACUNK4.16c | 0.53 |  |  |  |
| SPAC6G9.08 | 0.54 | n |  |  |
| SPBC26H8.09c | 0.54 | n |  |  |
| SPBC146.02 | 0.54 | n |  |  |
| SPAC27E2.03c | 0.54 | y |  |  |
| SPCC1223.04c(set11) | 0.54 | y | 5 | + |
| SPAC2F3.15 | 0.54 |  |  |  |
| SPBC365.02c | 0.55 | n |  |  |
| SPBC3E7.12c | 0.55 | n |  |  |
| SPAC27E2.02 | 0.55 | n |  |  |
| SPAC1805.16c | 0.55 | n |  |  |
| SPBC1683.03c | 0.55 | n |  |  |
| SPBC354.04 | 0.55 | n |  |  |
| SPBC21C3.14c | 0.55 | n |  |  |
| SPCC1902.01 | 0.55 | n |  |  |
| SPAC29A4.13 | 0.55 | n |  |  |
| SPBC1685.12c | 0.55 | n |  |  |
| SPAC11G7.01 | 0.55 |  |  |  |
| SPAC13C5.04 | 0.55 |  |  |  |
| SPAC186.08c | 0.55 |  |  |  |
| SPBC1709.11c | 0.56 | n |  |  |
| SPAC1687.05 | 0.56 | n | 1 | ± |
| SPAC328.01c | 0.56 | n |  |  |
| SPBC1703.03c | 0.56 | n |  |  |
| SPBC577.11 | 0.56 | n |  |  |
| SPBC106.13 | 0.56 | n |  |  |
| SPAC105.01c | 0.56 | n |  |  |
| SPCC1739.01 | 0.56 | n |  |  |
| SPBC16A3.08c | 0.56 | n |  |  |
| SPBC2F12.04 | 0.56 | y, n |  |  |
| SPCP31B10.05 | 0.56 |  |  |  |
| SPBC1709.14 | 0.57 | n |  |  |
| SPAC19D5.03 | 0.57 | n |  |  |
| SPAC16E8.05c | 0.57 | n |  |  |
| SPAC4F10.04 | 0.57 | y, y |  |  |
| SPBC18H10.10c | 0.57 |  |  |  |
| SPAC23D3.03c | 0.58 | n |  |  |
| SPBC337.15c | 0.58 | n |  |  |
| SPAC14C4.11 | 0.58 | n |  |  |
| SPAC27D7.02c | 0.58 | n |  |  |
| SPAC15E1.02c | 0.58 | n |  |  |
| SPBC1539.08 | 0.58 | n |  |  |
| SPBC1539.03c | 0.58 | n |  |  |
| SPBC28E12.06c | 0.58 | n |  |  |
| SPBPB2B2.15 | 0.58 | n |  |  |
| SPBPB2B2.13(gal1) | 0.58 | y |  |  |
| SPAC30D11.02c | 0.58 | y, n |  |  |
| SPAC750.04c | 0.58 |  |  |  |
| SPAC1687.14c | 0.59 | n |  |  |
| SPAC7D4.02c | 0.59 | n |  |  |
| SPBC106.07c | 0.59 | n |  |  |
| SPCC126.13c | 0.59 | n |  |  |
| SPBC839.17c | 0.59 | n |  |  |
| SPBC725.01 | 0.59 | n |  |  |
| SPBC1348.08c | 0.59 |  |  |  |
| SPAC6B12.05c | 0.59 |  |  |  |
| SPCP31B10.04 | 0.59 |  |  |  |
| SPAC4G8.04 | 0.60 | n |  |  |
| SPAC9.06c | 0.60 | n |  |  |
| SPBC19G7.03c | 0.60 | n |  |  |
| SPBC4.05 | 0.60 | n |  |  |
| SPAC167.08 | 0.60 | n |  |  |
| SPAC26A3.09c | 0.60 | n |  |  |
| SPBC713.05 | 0.60 | n |  |  |
| SPCC736.05 | 0.60 | n |  |  |
| SPAC1565.07c | 0.60 | n |  |  |
| SPAC13G7.04c(mac1) | 0.60 | y |  |  |
| SPAC4F10.13c(mpd2) | 0.60 | y |  |  |
| SPAC1782.04 | 0.60 |  |  |  |
| SPCP20C8.03 | 0.60 |  |  |  |
| SPAC24C9.14 | 0.61 | n |  |  |
| SPAC19G12.04 | 0.61 | n |  |  |
| SPAC29A4.02c | 0.61 | n |  |  |
| SPAC22H10.04 | 0.61 | n |  |  |
| SPBP22H7.05c | 0.61 | n |  |  |
| SPAC26F1.05 | 0.61 | n |  |  |
| SPCC1494.01 | 0.61 | n |  |  |
| SPCC663.14c | 0.61 | n |  |  |
| SPBC4F6.04(rpl2502) | 0.61 | y |  |  |
| SPAC17A2.14 | 0.61 |  |  |  |
| SPAC17C9.14 | 0.61 |  |  |  |
| SPAC664.04c | 0.62 | n |  |  |
| SPCC162.03 | 0.62 | n |  |  |
| SPAC23G3.04 | 0.62 | n |  |  |
| SPAC1805.12c | 0.62 | n |  |  |
| SPAC6G9.11 | 0.62 | n |  |  |
| SPAC14C4.14(atp1) | 0.62 | y |  |  |
| SPCC306.04c | 0.62 | y, n |  |  |
| SPAC19E9.02 | 0.62 | y, n |  |  |
| SPAC1F12.05 | 0.62 |  |  |  |
| SPAC26F1.04c | 0.63 | n |  |  |
| SPAC30C2.04 | 0.63 | n |  |  |
| SPBC16E9.01c | 0.63 | n |  |  |
| SPCC70.04c | 0.63 | n |  |  |
| SPAC1805.15c | 0.63 | n | 5± | n |
| SPAC823.13c | 0.63 | n |  |  |
| SPAC806.08c(mod21) | 0.63 | y |  |  |
| SPBPB7E8.01 | 0.63 | y |  |  |
| SPCC191.11 | 0.63 | y, n |  |  |
| SPAC1782.11 | 0.63 |  |  |  |
| SPAC222.15 | 0.64 | n |  |  |
| SPAC589.06c | 0.64 | n |  |  |
| SPAC23H3.05c | 0.64 | n |  |  |
| SPAC31G5.18c | 0.64 | n |  |  |
| SPBC28F2.05c | 0.64 | n |  |  |
| SPBC25B2.10 | 0.64 | n |  |  |
| SPBC725.07 | 0.64 | n | 5-/+ | n |
| SPCC4B3.02c | 0.64 | n |  |  |
| SPAC6F12.03c | 0.64 | n |  |  |
| SPAC688.04c | 0.64 | n |  |  |
| SPBP4G3.02(pho1) | 0.64 | y |  |  |
| SPAC23E2.01(fep1) | 0.64 | y |  |  |
| SPBC32F12.07c | 0.64 | y, n |  |  |
| SPAC1952.05 | 0.64 |  |  |  |
| SPBC887.08 | 0.64 |  |  |  |
| SPBC4B4.04 | 0.65 | n |  |  |
| SPBC17G9.05 | 0.65 | n |  |  |
| SPBC543.03c | 0.65 | n |  |  |
| SPAC1B3.01c | 0.65 | n |  |  |
| SPBC660.12c | 0.65 | n |  |  |
| SPAC3A12.17c | 0.65 | n | 5± | n |
| SPAC23C4.12 | 0.65 | n |  |  |
| SPBC1289.10c | 0.65 | n |  |  |
| SPCC613.08 | 0.65 | n |  |  |
| SPAC23H4.08 | 0.65 | n |  |  |
| SPBC26H8.05c | 0.65 | y, n |  |  |
| SPBC1198.06c | 0.65 | y, n |  |  |
| SPCC757.12 | 0.65 |  |  |  |
| SPCC1620.12c | 0.66 | n |  |  |
| SPCC1235.05c | 0.66 | n |  |  |
| SPAC890.03 | 0.66 | n |  |  |
| SPACUNK12.02c | 0.66 | n |  |  |
| SPAC57A7.09 | 0.66 | n |  |  |
| SPAC24C9.02c | 0.66 | n |  |  |
| SPBC1709.05 | 0.66 | n |  |  |
| SPBC16A3.06 | 0.66 | n |  |  |
| SPAC31A2.14 | 0.66 | n |  |  |
| SPBC8D2.12c | 0.66 | n | 5± | n |
| SPAPB1A11.03 | 0.66 | n |  |  |
| SPAC2E1P5.03 | 0.66 | n |  |  |
| SPBC12C2.05c | 0.66 | n |  |  |
| SPAC688.10 | 0.66 | y, n |  |  |
| SPAC637.09 | 0.66 |  |  |  |
| SPBC713.09 | 0.66 |  |  |  |
| SPBC1773.16c | 0.67 | n |  |  |
| SPCC74.09 | 0.67 | n |  |  |
| SPBC30D10.18c | 0.67 | n |  |  |
| SPBC25B2.01 | 0.67 | n |  |  |
| SPAC1296.01c | 0.67 | n |  |  |
| SPAC23A1.17 | 0.67 | n |  |  |
| SPAC14C4.10c | 0.67 | n |  |  |
| SPBC2D10.18 | 0.67 | n |  |  |
| SPBC2G2.09c | 0.67 | n |  |  |
| SPBC354.11c | 0.67 | n |  |  |
| SPCC13B11.02c | 0.67 | n |  |  |
| SPBC4.01 | 0.67 | n |  |  |
| SPAC1399.05c | 0.67 | n |  |  |
| SPAPB8E5.08 | 0.67 | n |  |  |
| SPBC1198.14c | 0.67 | n |  |  |
| SPAC1002.12c | 0.67 | n |  |  |
| SPAC1751.01c | 0.67 |  |  |  |
| SPAC323.03c | 0.67 |  |  |  |
| SPCPJ732.02c | 0.67 |  |  |  |
| SPCC622.19 | 0.67 |  |  |  |
| SPBC887.16 | 0.67 |  |  |  |
| SPCC1682.01 | 0.67 |  |  |  |
| SPBC582.08 | 0.68 | n |  |  |
| SPBP8B7.25 | 0.68 | n |  |  |
| SPCC1795.03 | 0.68 | n |  |  |
| SPAC12G12.07c | 0.68 | n |  |  |
| SPAC29B12.12 | 0.68 | n |  |  |
| SPAC25H1.05 | 0.68 | n |  |  |
| SPAC31G5.11 | 0.68 | n |  |  |
| SPCC4E9.01c | 0.68 | n |  |  |
| SPBP8B7.10c | 0.68 | n |  |  |
| SPAC17H9.01 | 0.68 |  |  |  |
| SPBC11C11.12 | 0.68 |  |  |  |
| SPAC2G11.13 | 0.68 |  |  |  |
| SPAPB1A10.15 | 0.68 |  |  |  |
| SPBC8D2.01 | 0.69 | n |  |  |
| SPAC26A3.02 | 0.69 | n |  |  |
| SPCC24B10.03 | 0.69 | n |  |  |
| SPBC14C8.03 | 0.69 | n |  |  |
| SPAC6F6.17 | 0.69 | n |  |  |
| SPBC685.03 | 0.69 | n |  |  |
| SPCC663.06c | 0.69 | n |  |  |
| SPBC651.04 | 0.69 | n |  |  |
| SPBPB2B2.18 | 0.69 | n |  |  |
| SPBC405.01 | 0.69 | y, n |  |  |
| SPAPB1E7.05 | 0.69 |  |  |  |
| SPAC20H4.03c | 0.69 |  |  |  |
| SPAC23C4.02 | 0.70 | n |  |  |
| SPAC17G6.13 | 0.70 | n |  |  |
| SPBC577.13 | 0.70 | n |  |  |
| SPAC3H1.10 | 0.70 | n |  |  |
| SPAC19G12.15c | 0.70 | n |  |  |
| SPCC13B11.01 | 0.70 | n |  |  |
| SPBC8D2.12c | 0.70 | n |  |  |
| SPCC1235.02 | 0.70 | n |  |  |
| SPAC869.05c | 0.70 | n |  |  |
| SPCC584.03c | 0.70 | y |  |  |
| SPAC13G7.02c(ssa1) | 0.70 | y |  |  |
| SPAC3G6.06c | 0.70 | y, n |  |  |
| SPBC20F10.05 | 0.70 | y, n |  |  |
| SPBC409.20c | 0.70 | y, n |  |  |
| SPAC57A7.07c | 0.70 |  |  |  |
| SPAP27G11.02 | 0.70 |  |  |  |
| SPAC977.03 | 0.70 |  |  |  |
| SPAC17H9.06c | 0.70 |  |  |  |
| SPAC19B12.06c | 0.71 | n |  |  |
| SPBC1347.02 | 0.71 | n |  |  |
| SPCC1672.03c | 0.71 | n |  |  |
| SPAC1B3.03c | 0.71 | n |  |  |
| SPAC23H4.08 | 0.71 | n |  |  |
| SPAC2F7.17 | 0.71 | n |  |  |
| SPBC17D11.03c | 0.71 | n |  |  |
| SPBC23E6.10c | 0.71 | n |  |  |
| SPCC1322.02 | 0.71 | n |  |  |
| SPAPB24D3.05c | 0.71 | n |  |  |
| SPAC1805.03c | 0.71 | n |  |  |
| SPAC4G8.13c | 0.71 | n |  |  |
| SPAC9G1.08c | 0.71 | n |  |  |
| SPBC106.04 | 0.71 | y, n |  |  |
| SPBC16A3.01 | 0.71 | y, n |  |  |
| SPAC8C9.04 | 0.71 |  |  |  |
| SPAC18B11.03c | 0.71 |  |  |  |
| SPAC22E12.08 | 0.71 |  |  |  |
| SPAC9.12c | 0.72 | n |  |  |
| SPCC23B6.05c | 0.72 | n |  |  |
| SPAC2E12.03c | 0.72 | n |  |  |
| SPAC27F1.03c | 0.72 | n |  |  |
| SPAC31G5.21 | 0.72 | n |  |  |
| SPBC16C6.08c | 0.72 | n |  |  |
| SPBC83.18c | 0.72 | n |  |  |
| SPBC800.02 | 0.72 | n |  |  |
| SPBC8E4.02c | 0.72 | n |  |  |
| SPAC17G6.17 | 0.72 | n |  |  |
| SPCC126.10 | 0.72 | n |  |  |
| SPCC569.06 | 0.72 | n |  |  |
| SPBC12C2.09c | 0.72 | n |  |  |
| SPBP19A11.07c | 0.72 | n |  |  |
| SPAC29A4.19c | 0.72 | n |  |  |
| SPAC694.06c | 0.72 | n |  |  |
| SPAC23H4.16c | 0.72 | n | 2 | n |
| SPAC11E3.13c(gas5) | 0.72 | y |  |  |
| SPBC2F12.05c | 0.72 | y |  |  |
| SPAC2C4.07c | 0.72 | y, n |  |  |
| SPAC890.06 | 0.72 |  |  |  |
| SPAC5H10.06c | 0.72 |  |  |  |
| SPAC3F10.10c | 0.73 | n |  |  |
| SPBC839.05c | 0.73 | n | 5-/+ | n |
| SPCP25A2.02c | 0.73 | n |  |  |
| SPAPYUG7.04c | 0.73 | n |  |  |
| SPAC12B10.07 | 0.73 | n |  |  |
| SPAC9.11 | 0.73 | n |  |  |
| SPBC18H10.18c | 0.73 | n |  |  |
| SPCC622.15c | 0.73 | n |  |  |
| SPCC663.08c | 0.73 | n |  |  |
| SPAC6C3.04 | 0.73 | n |  |  |
| SPBC839.07 | 0.73 | n |  |  |
| SPAC23G3.07c | 0.73 | n |  |  |
| SPAC1782.02c | 0.73 | y |  |  |
| SPAP27G11.08c | 0.73 |  |  |  |
| SPBC359.06 | 0.74 | n |  |  |
| SPAC25H1.02 | 0.74 | n |  |  |
| SPBC16H5.05c | 0.74 | n |  |  |
| SPCC1183.04c | 0.74 | n |  |  |
| SPAC4H3.07c | 0.74 | n |  |  |
| SPAC9G1.11c | 0.74 | n |  |  |
| SPAC1565.01 | 0.74 | n |  |  |
| SPBC18H10.05 | 0.74 | n |  |  |
| SPAC29E6.01 | 0.74 | n |  |  |
| SPAC1805.05 | 0.74 | n |  |  |
| SPBC947.10 | 0.74 | n |  |  |
| SPBC16G5.11c | 0.74 | n |  |  |
| SPCC18B5.09c | 0.74 | n |  |  |
| SPAC24C9.08 | 0.74 | n |  |  |
| SPAC18G6.12c | 0.74 | y |  |  |
| SPBC56F2.06(mug147) | 0.74 | y |  |  |
| SPAC15E1.10 | 0.74 |  |  |  |
| SPAC6B12.08 | 0.74 |  |  |  |
| SPAC20H4.08 | 0.74 |  |  |  |
| SPAC19B12.11c | 0.75 | n |  |  |
| SPAC3A12.12 | 0.75 | n |  |  |
| SPBC3E7.05c | 0.75 | n |  |  |
| SPAC22H12.04c | 0.75 | n |  |  |
| SPBC530.14c | 0.75 | n | 5-/+ | n |
| SPCC338.04 | 0.75 | n |  |  |
| SPAC4C5.01 | 0.75 | n |  |  |
| SPAC15F9.01c | 0.75 | n |  |  |
| SPAC16.02c | 0.75 | n |  |  |
| SPBC646.17c | 0.75 | n |  |  |
| SPAC31A2.15c | 0.75 | n |  |  |
| SPBC19C2.02 | 0.75 | n |  |  |
| SPBC1773.08c | 0.75 | n |  |  |
| SPBC725.04 | 0.75 | n |  |  |
| SPCC11E10.01 | 0.75 | n |  |  |
| SPCC1393.02c | 0.75 | n |  |  |
| SPBC691.03c | 0.75 | n |  |  |
| SPBC428.07 | 0.75 | n |  |  |
| SPCC622.03c | 0.75 | n |  |  |
| SPAC23A1.14c | 0.75 | n |  |  |
| SPAC31G5.10 | 0.75 | n |  |  |
| SPAC4G9.20c | 0.75 | n |  |  |
| SPAC630.05 | 0.75 | n |  |  |
| SPCP1E11.10 | 0.75 | n | 5-/+ | n |
| SPBC13E7.01(cwf22) | 0.75 | y |  |  |
| SPAC139.05 | 0.75 |  |  |  |
| SPAC1039.06 | 0.75 |  |  |  |
| SPAC3H5.11 | 0.75 |  |  |  |
| SPBC660.11 | 0.75 |  |  |  |
| SPAC806.05 | 0.75 |  |  |  |
| SPCC613.02 | 0.76 | n |  |  |
| SPCC1259.02c | 0.76 | n |  |  |
| SPAC26F1.14c | 0.76 | n |  |  |
| SPBC27.04 | 0.76 | n |  |  |
| SPAC3H8.03 | 0.76 | n |  |  |
| SPAC27D7.13c | 0.76 | n |  |  |
| SPAC1556.04c | 0.76 | n |  |  |
| SPAC24B11.14 | 0.76 | n |  |  |
| SPAC25G10.02 | 0.76 | n |  |  |
| SPBC1685.02c | 0.76 | n |  |  |
| SPBC2F12.15c | 0.76 | n | 6 | n |
| SPBC106.19 | 0.76 | n |  |  |
| SPAC1687.16c | 0.76 | n |  |  |
| SPAC1B3.20 | 0.76 | n |  |  |
| SPBC1271.05c | 0.76 | n |  |  |
| SPBC30D10.14 | 0.76 | n |  |  |
| SPBC1271.06c | 0.76 | n |  |  |
| SPBC15D4.06 | 0.76 | n |  |  |
| SPBC21B10.08c | 0.76 | y |  |  |
| SPBC30B4.03c | 0.76 |  |  |  |
| SPAC4A8.02c | 0.76 |  |  |  |
| SPAC688.03c | 0.76 |  |  |  |
| SPAC29B12.14c | 0.77 | n |  |  |
| SPAC4G9.11c | 0.77 | n |  |  |
| SPAC823.14 | 0.77 | n |  |  |
| SPAC2C4.17c | 0.77 | n | 5-/+ | n |
| SPAC8C9.17c | 0.77 | n |  |  |
| SPAC15A10.10 | 0.77 | n |  |  |
| SPBC2A9.03 | 0.77 | n |  |  |
| SPAC22H10.03c | 0.77 | n |  |  |
| SPBC1289.09 | 0.77 | n |  |  |
| SPBC1709.13c | 0.77 | n |  |  |
| SPBC651.06 | 0.77 | n |  |  |
| SPCC1020.08 | 0.77 | n |  |  |
| SPCC576.02 | 0.77 | n |  |  |
| SPCP1E11.05c | 0.77 | n |  |  |
| SPCC70.08c | 0.77 | n |  |  |
| SPCC576.17c | 0.77 | n |  |  |
| SPCC613.11c | 0.77 | n |  |  |
| SPAC227.10 | 0.77 | n |  |  |
| SPAPB1A10.12c | 0.77 | n |  |  |
| SPBPB2B2.07c | 0.77 | n |  |  |
| SPBC2G5.03 | 0.77 | n |  |  |
| SPAC19G12.13c | 0.77 | n |  |  |
| SPBC216.01c | 0.77 | y |  |  |
| SPAC14C4.06c | 0.77 | y |  |  |
| SPAC1002.06c | 0.77 |  |  |  |
| SPAC1A6.07 | 0.77 |  |  |  |
| SPCC1450.09c | 0.77 |  |  |  |
| SPCC1450.12 | 0.77 |  |  |  |
| SPAC17C9.16c | 0.77 |  |  |  |
| SPAC1805.02c | 0.78 | n |  |  |
| SPCC4E9.02 | 0.78 | n |  |  |
| SPAC1783.08c | 0.78 | n |  |  |
| SPCC338.11c | 0.78 | n |  |  |
| SPAC14C4.08 | 0.78 | n |  |  |
| SPAC25B8.09 | 0.78 | n |  |  |
| SPBC25B2.08 | 0.78 | n |  |  |
| SPCC1494.09c | 0.78 | n |  |  |
| SPBC649.02 | 0.78 | n |  |  |
| SPBP18G5.03 | 0.78 | n |  |  |
| SPCC1235.11 | 0.78 | n |  |  |
| SPBC29A10.05 | 0.78 | y, n |  |  |
| SPBC336.10c | 0.78 | y, n |  |  |
| SPBC25H2.14 | 0.78 | y, n |  |  |
| SPAC589.11 | 0.78 |  |  |  |
| SPAC26A3.10 | 0.79 | n |  |  |
| SPBC365.01 | 0.79 | n |  |  |
| SPAC3F10.11c | 0.79 | n |  |  |
| SPAC1F7.11c | 0.79 | n |  |  |
| SPAC2F3.02 | 0.79 | n |  |  |
| SPCC320.03 | 0.79 | n | 5± | n |
| SPAC227.14 | 0.79 | n |  |  |
| SPAC2F3.16 | 0.79 | n |  |  |
| SPAC22H12.01c | 0.79 | n |  |  |
| SPAC977.12 | 0.79 | n |  |  |
| SPBC1861.06c | 0.79 | n |  |  |
| SPBC19C7.12c | 0.79 | n |  |  |
| SPBC19G7.07c | 0.79 | n |  |  |
| SPBC3E7.07c | 0.79 | n |  |  |
| SPBP8B7.31 | 0.79 | n |  |  |
| SPBC19F5.01c | 0.79 | n |  |  |
| SPCC338.02 | 0.79 | n |  |  |
| SPCC4G3.11 | 0.79 | n |  |  |
| SPCC306.10 | 0.79 | n |  |  |
| SPBC409.11 | 0.79 | n |  |  |
| SPCC663.10 | 0.79 | n |  |  |
| SPAPB8E5.04c | 0.79 | n |  |  |
| SPAC4A8.03c | 0.79 | n |  |  |
| SPCC417.09c | 0.79 | n |  |  |
| SPBC2G2.13c | 0.79 | n | 5 | n |
| SPAC57A7.08(pzh1) | 0.79 | y |  |  |
| SPBC18H10.15 | 0.79 | y, n |  |  |
| SPAC12G12.12 | 0.79 | y, n |  |  |
| SPAC6C3.07 | 0.79 |  |  |  |
| SPCC584.15c | 0.79 |  |  |  |
| SPAPB1E7.08c | 0.79 |  |  |  |
| SPAC31A2.16 | 0.80 | n |  |  |
| SPAC57A10.09c | 0.80 | n |  |  |
| SPBC146.04 | 0.80 | n |  |  |
| SPAC31G5.07 | 0.80 | n |  |  |
| SPAC17C9.11c | 0.80 | n |  |  |
| SPBC32H8.08c | 0.80 | n |  |  |
| SPBC3H7.07c | 0.80 | n |  |  |
| SPAC23D3.01 | 0.80 | n |  |  |
| SPCC645.05c | 0.80 | n |  |  |
| SPAC6G10.02c | 0.80 | n |  |  |
| SPBC800.11 | 0.80 | n |  |  |
| SPAC29B12.13 | 0.80 | n |  |  |
| SPCC1020.02 | 0.80 | n |  |  |
| SPBP4H10.17c | 0.80 | n |  |  |
| SPCC1442.17c | 0.80 | n |  |  |
| SPCC417.12 | 0.80 | n |  |  |
| SPAC5H10.05c | 0.80 | n |  |  |
| SPAC823.09c | 0.80 | n |  |  |
| SPCC663.11 | 0.80 | n |  |  |
| SPBC30B4.02c | 0.80 | n |  |  |
| SPBC16G5.16 | 0.80 | n |  |  |
| SPBC115.03 | 0.80 | y |  |  |
| SPBC2G2.10c(mug110) | 0.80 | y |  |  |
| SPBC32H8.02c(nep2) | 0.80 | y |  |  |
| SPAPB1A11.04c | 0.80 | y |  |  |
| SPAC13G7.03 | 0.80 |  |  |  |
| SPAC869.08 | 0.80 |  |  |  |
| SPBP4H10.08 | 0.80 |  |  |  |
| SPAC869.06c | 0.80 |  |  |  |
| SPAC6B12.04c | 0.81 | n |  |  |
| SPBC4F6.08c | 0.81 | n |  |  |
| SPBC29A10.14 | 0.81 | n | 5-/+ | n |
| SPAC6F12.12 | 0.81 | n |  |  |
| SPBC1683.06c | 0.81 | n |  |  |
| SPAC23A1.03 | 0.81 | n |  |  |
| SPAC22F8.03c | 0.81 | n |  |  |
| SPBC1347.11 | 0.81 | n |  |  |
| SPBC1683.11c | 0.81 | n |  |  |
| SPCC1450.16c | 0.81 | n |  |  |
| SPBC2A9.04c | 0.81 | n |  |  |
| SPBC36B7.05c | 0.81 | n |  |  |
| SPAC4G9.14 | 0.81 | n |  |  |
| SPBC1198.07c | 0.81 | y, n |  |  |
| SPAC12B10.12c | 0.81 |  | 5 | n |
| SPBC1348.12 | 0.81 |  |  |  |
| SPAC17C9.05c | 0.81 |  |  |  |
| SPAC227.11c | 0.81 |  |  |  |
| SPBC1709.04c | 0.82 | n |  |  |
| SPAC1F3.05 | 0.82 | n |  |  |
| SPBC2G5.02c | 0.82 | n |  |  |
| SPAC26H5.03 | 0.82 | n |  |  |
| SPAC3C7.05c | 0.82 | n |  |  |
| SPBC1271.09 | 0.82 | n |  |  |
| SPCC1840.06 | 0.82 | n |  |  |
| SPAC8E11.06 | 0.82 | n |  |  |
| SPBC12C2.01c | 0.82 | n |  |  |
| SPBC19C2.06c | 0.82 | n |  |  |
| SPBC409.06 | 0.82 | n |  |  |
| SPCC1223.03c | 0.82 | n |  |  |
| SPBC19C7.08c | 0.82 | n |  |  |
| SPCC576.16c | 0.82 | n |  |  |
| SPCC622.16c | 0.82 | n |  |  |
| SPAC343.11c | 0.82 | n |  |  |
| SPAC3A11.09 | 0.82 | n |  |  |
| SPAC4F10.02 | 0.82 | n |  |  |
| SPCC162.08c | 0.82 | n |  |  |
| SPBPJ4664.01(dps1) | 0.82 | y |  |  |
| SPBC18A7.02c | 0.82 | y, n |  |  |
| SPBC19C7.01 | 0.82 | y, n |  |  |
| SPAC9G1.04 | 0.82 |  |  |  |
| SPAC926.06c | 0.82 |  |  |  |
| SPAC1B3.15c | 0.82 |  |  |  |
| SPCP1E11.11 | 0.82 |  |  |  |
| SPAC652.01 | 0.82 |  |  |  |
| SPAPB1A10.14 | 0.82 |  |  |  |
| SPBC11G11.03 | 0.83 | n |  |  |
| SPBC839.04 | 0.83 | n |  |  |
| SPAC607.06c | 0.83 | n |  |  |
| SPAC1002.05c | 0.83 | n |  |  |
| SPBC354.05c | 0.83 | n |  |  |
| SPAC6F12.09 | 0.83 | n |  |  |
| SPAC1687.07 | 0.83 | n |  |  |
| SPAC24H6.10c | 0.83 | n |  |  |
| SPAC31A2.12 | 0.83 | n |  |  |
| SPAC8E11.04c | 0.83 | n |  |  |
| SPBC11C11.06c | 0.83 | n |  |  |
| SPBC26H8.13c | 0.83 | n |  |  |
| SPBC365.20c | 0.83 | n |  |  |
| SPAC14C4.15c | 0.83 | n |  |  |
| SPCC126.02c | 0.83 | n |  |  |
| SPAPB8E5.06c | 0.83 | n |  |  |
| SPAC25H1.07 | 0.83 | n |  |  |
| SPBC16C6.05 | 0.83 | n |  |  |
| SPAC644.08 | 0.83 | n |  |  |
| SPAC17A5.01(pex6) | 0.83 | y |  |  |
| SPBC530.06c | 0.83 | y |  |  |
| SPAC1705.03c(ecm33) | 0.83 | y |  |  |
| SPCC895.09c(ucp12) | 0.83 | y |  |  |
| SPAC13G6.13 | 0.83 |  |  |  |
| SPAC15A10.07 | 0.83 |  |  |  |
| SPAC1F7.08 | 0.83 |  |  |  |
| SPBC3D6.02 | 0.83 |  |  |  |
| SPAC694.03 | 0.83 |  |  |  |
| SPAC26A3.16 | 0.84 | n |  |  |
| SPAC23H4.02 | 0.84 | n |  |  |
| SPAC1687.06c | 0.84 | n |  |  |
| SPAC14C4.12c | 0.84 | n |  |  |
| SPBC1539.04 | 0.84 | n |  |  |
| SPBC317.01 | 0.84 | n |  |  |
| SPBC2D10.04 | 0.84 | n |  |  |
| SPBC3E7.11c | 0.84 | n |  |  |
| SPBC21C3.06 | 0.84 | n |  |  |
| SPCC1494.11c | 0.84 | n |  |  |
| SPBC713.10 | 0.84 | n |  |  |
| SPBC16E9.02c | 0.84 | n |  |  |
| SPAC2F7.09c | 0.84 | n |  |  |
| SPBP16F5.02 | 0.84 | n |  |  |
| SPBC15D4.06 | 0.84 | n |  |  |
| SPAC20G8.08c | 0.84 | n |  |  |
| SPAC12G12.15(sif3) | 0.84 | y |  |  |
| SPAP14E8.02 | 0.84 | y, n |  |  |
| SPAC1F12.03c | 0.84 |  |  |  |
| SPAC20G8.07c | 0.84 |  |  |  |
| SPBC800.12c | 0.84 |  |  |  |
| SPAC8E11.01c | 0.84 |  |  |  |
| SPAP8A3.04c | 0.84 |  |  |  |
| SPAC1805.13 | 0.85 | n |  |  |
| SPAC694.02 | 0.85 | n |  |  |
| SPBC1347.12 | 0.85 | n |  |  |
| SPAC12B10.03 | 0.85 | n |  |  |
| SPBP35G2.10 | 0.85 | n |  |  |
| SPAC29A4.14c | 0.85 | n |  |  |
| SPBC106.16 | 0.85 | n |  |  |
| SPBC1703.09 | 0.85 | n |  |  |
| SPBC29A10.16c | 0.85 | n |  |  |
| SPBC337.11 | 0.85 | n |  |  |
| SPCC1183.11 | 0.85 | n |  |  |
| SPAC1783.02c | 0.85 | n |  |  |
| SPCC1020.06c | 0.85 | n |  |  |
| SPAPB2C8.01 | 0.85 | n |  |  |
| SPCC736.09c | 0.85 | n |  |  |
| SPBC32H8.13c | 0.85 | n |  |  |
| SPAC27D7.11c | 0.85 | y, n |  |  |
| SPAC17A5.10 | 0.85 |  |  |  |
| SPAC1639.02c | 0.85 |  |  |  |
| SPAC6C3.02c | 0.85 |  |  |  |
| SPCC24B10.12 | 0.85 |  |  |  |
| SPCC16A11.08 | 0.85 |  |  |  |
| SPBC29A10.09c | 0.86 | n |  |  |
| SPAC1805.11c | 0.86 | n |  |  |
| SPBC1703.06 | 0.86 | n |  |  |
| SPBC29A10.10c | 0.86 | n |  |  |
| SPAC1556.03 | 0.86 | n |  |  |
| SPAC23G3.03 | 0.86 | n |  |  |
| SPAC589.08c | 0.86 | n |  |  |
| SPBC32F12.03c | 0.86 | n |  |  |
| SPAPB24D3.08c | 0.86 | n |  |  |
| SPBC691.05c | 0.86 | n |  |  |
| SPBC8D2.19 | 0.86 | n |  |  |
| SPAC1006.04c | 0.86 |  |  |  |
| SPAC1327.01c | 0.86 |  |  |  |
| SPAC1952.10c | 0.86 |  |  |  |
| SPAC4D7.11 | 0.86 |  |  |  |
| SPCPB16A4.05c | 0.86 |  |  |  |
| SPCC126.12 | 0.87 | n |  |  |
| SPBC12D12.06 | 0.87 | n |  |  |
| SPCC330.14c | 0.87 | n |  |  |
| SPAC2G11.05c | 0.87 | n |  |  |
| SPAPB1A11.01 | 0.87 | n |  |  |
| SPAC26H5.07c | 0.87 | n |  |  |
| SPAC25B8.07c | 0.87 | n |  |  |
| SPBC1105.09 | 0.87 | n |  |  |
| SPBC1709.16c | 0.87 | n |  |  |
| SPBC1289.16c | 0.87 | n |  |  |
| SPCC364.01 | 0.87 | n |  |  |
| SPCC645.12c | 0.87 | n |  |  |
| SPCC24B10.02c | 0.87 | n |  |  |
| SPCC622.14 | 0.87 | n |  |  |
| SPCC24B10.13(skb5) | 0.87 | y |  |  |
| SPAC343.12 (rds1) | 0.87 | y |  |  |
| SPBC19F8.06c | 0.87 | y, n |  |  |
| SPBC16H5.11c | 0.87 | y, n |  |  |
| SPAPYUG7.06 | 0.87 | y, n |  |  |
| SPAC9E9.12c | 0.87 |  |  |  |
| SPAC1002.02 | 0.87 |  |  |  |
| SPAC1039.08 | 0.87 |  |  |  |
| SPAC1002.01 | 0.87 |  |  |  |
| SPAC17C9.01c | 0.87 |  |  |  |
| SPBC4B4.11 | 0.87 |  |  |  |
| SPAC12G12.03 | 0.88 | n |  |  |
| SPBC14F5.07 | 0.88 | n |  |  |
| SPBC28F2.11 | 0.88 | n |  |  |
| SPAC23G3.12c | 0.88 | n |  |  |
| SPBC725.02 | 0.88 | n |  |  |
| SPCC622.17 | 0.88 | n |  |  |
| SPCC1442.07c | 0.88 | n |  |  |
| SPAC29B12.05c | 0.88 | n |  |  |
| SPAC2G11.04 | 0.88 | n |  |  |
| SPCC18.02 | 0.88 | n |  |  |
| SPBC11C11.01 | 0.88 | n |  |  |
| SPBC12C2.12c | 0.88 | n |  |  |
| SPBC685.04c | 0.88 | n |  |  |
| SPCC1322.07c | 0.88 | n |  |  |
| SPBC32H8.01c | 0.88 | n |  |  |
| SPBC713.07c | 0.88 | n |  |  |
| SPAPB24D3.03 | 0.88 | n |  |  |
| SPBC18H10.08c(ubp4) | 0.88 | y |  |  |
| SPAC1002.03c | 0.88 | y, n |  |  |
| SPAC1093.03 | 0.88 |  |  |  |
| SPAC10F6.14c | 0.88 |  |  |  |
| SPAC11D3.11c | 0.88 |  |  |  |
| SPAC1B3.10c | 0.88 |  |  |  |
| SPAC4G9.12 | 0.88 |  |  |  |
| SPAC4H3.05 | 0.88 |  | 5 | n |
| SPAC57A10.04 | 0.88 |  |  |  |
| SPAC458.06 | 0.88 |  |  |  |
| SPBC2D10.16 | 0.88 |  |  |  |
| SPBC26H8.12 | 0.89 | n |  |  |
| SPAC11D3.17 | 0.89 | n | 5-/+ | n |
| SPAC24B11.08c | 0.89 | n |  |  |
| SPAC688.13 | 0.89 | n |  |  |
| SPAC1486.02c | 0.89 | n |  |  |
| SPAC23D3.05c | 0.89 | n |  |  |
| SPAC3C7.02c | 0.89 | n |  |  |
| SPAC8E11.10 | 0.89 | n |  |  |
| SPBC2G5.01 | 0.89 | n |  |  |
| SPBC530.03c | 0.89 | n |  |  |
| SPBC6B1.03c | 0.89 | n |  |  |
| SPCC306.02c | 0.89 | n |  |  |
| SPBPB10D8.05c | 0.89 | n |  |  |
| SPCC70.09c | 0.89 | n |  |  |
| SPBC11B10.05c | 0.89 | n |  |  |
| SPCC1259.14c | 0.89 | n |  |  |
| SPCC1281.04 | 0.89 | n |  |  |
| SPAC3A12.03c | 0.89 | n |  |  |
| SPAC227.05 | 0.89 | y |  |  |
| SPAC139.02c | 0.89 |  |  |  |
| SPAC11H11.05c | 0.89 |  |  |  |
| SPAC11D3.01c | 0.89 |  |  |  |
| SPAC11D3.04c | 0.89 |  |  |  |
| SPAC4F10.08 | 0.89 |  |  |  |
| SPAC5H10.10 | 0.89 |  |  |  |
| SPAC513.07 | 0.89 |  |  |  |
| SPBC13A2.04c | 0.89 |  |  |  |
| SPCPB16A4.02c | 0.89 |  |  |  |
| SPAC9.10 | 0.90 | n |  |  |
| SPAC14C4.05c | 0.90 | n |  |  |
| SPAC1250.05 | 0.90 | n |  |  |
| SPBC2G2.06c | 0.90 | n |  |  |
| SPCC126.01c | 0.90 | n |  |  |
| SPCC11E10.09c | 0.90 | n |  |  |
| SPAC2C4.05 | 0.90 | n |  |  |
| SPBC1D7.01 | 0.90 | n | 5-/+ | n |
| SPBP4H10.04 | 0.90 | y, n |  |  |
| SPAC3F10.02c | 0.90 | y, n |  |  |
| SPBC16A3.14 | 0.90 |  |  |  |
| SPAC11D3.16c | 0.90 |  |  |  |
| SPAC3H8.04 | 0.90 |  |  |  |
| SPAC9E9.17c | 0.90 |  |  |  |
| SPBC13E7.08c | 0.90 |  |  |  |
| SPCC1620.07c | 0.90 |  |  |  |
| SPAC1D4.13 | 0.91 | n |  |  |
| SPAC222.07c | 0.91 | n |  |  |
| SPAC3F10.06c | 0.91 | n |  |  |
| SPBC29B5.02c | 0.91 | n |  |  |
| SPBC1709.18 | 0.91 | n |  |  |
| SPBC31F10.07 | 0.91 | n |  |  |
| SPAC13G7.11 | 0.91 | n |  |  |
| SPAC2E12.05 | 0.91 | n |  |  |
| SPAC22F8.05 | 0.91 | n |  |  |
| SPAC30D11.05 | 0.91 | n |  |  |
| SPAC3C7.01c | 0.91 | n |  |  |
| SPBC1271.14 | 0.91 | n |  |  |
| SPBC29A10.11c | 0.91 | n |  |  |
| SPBC405.05 | 0.91 | n |  |  |
| SPBC4C3.03 | 0.91 | n |  |  |
| SPAC16C9.01c | 0.91 | n |  |  |
| SPAC3H8.08c | 0.91 | n |  |  |
| SPAC1B3.04c | 0.91 | n |  |  |
| SPBC902.02c | 0.91 | n |  |  |
| SPBC354.15 | 0.91 | n |  |  |
| SPCP1E11.03(mug170) | 0.91 | y |  |  |
| SPAC869.10c | 0.91 |  |  |  |
| SPAC6F6.11c | 0.91 |  |  |  |
| SPBC16G5.03 | 0.91 |  |  |  |
| SPBPB2B2.19c | 0.91 |  |  |  |
| SPCC4B3.05c | 0.91 |  |  |  |
| SPBC83.04 | 0.91 |  |  |  |
| SPBC3H7.06c | 0.91 |  |  |  |
| SPAC1F8.01 | 0.92 | n |  |  |
| SPBC4C3.04c | 0.92 | n |  |  |
| SPBP4H10.16c | 0.92 | n |  |  |
| SPAC26H5.02c | 0.92 | n |  |  |
| SPCC18B5.05c | 0.92 | n |  |  |
| SPAC16C9.02c | 0.92 | n |  |  |
| SPBC1683.12 | 0.92 | n |  |  |
| SPBC1861.02 | 0.92 | n |  |  |
| SPBC11C11.11c | 0.92 | n |  |  |
| SPBC3H7.12 | 0.92 | n |  |  |
| SPAC1782.07 | 0.92 | n |  |  |
| SPCC330.02 | 0.92 | n |  |  |
| SPAC26F1.12c | 0.92 | n |  |  |
| SPAC56F8.06c(alg10) | 0.92 | y |  |  |
| SPCC306.11 | 0.92 | y |  |  |
| SPBC1348.10c | 0.92 |  |  |  |
| SPAC11E3.14 | 0.92 |  |  |  |
| SPAC688.12c | 0.92 |  |  |  |
| SPAC8F11.09c | 0.92 |  |  |  |
| SPCC16C4.04 | 0.92 |  |  |  |
| SPAC1782.06c | 0.93 | n |  |  |
| SPAC6G9.05 | 0.93 | n |  |  |
| SPAC13G6.04 | 0.93 | n |  |  |
| SPBC115.02c | 0.93 | n |  |  |
| SPAC3C7.14c | 0.93 | n |  |  |
| SPBP4H10.13 | 0.93 | n |  |  |
| SPCC4G3.02 | 0.93 | n |  |  |
| SPAC1F7.09c | 0.93 | n |  |  |
| SPAC637.11 | 0.93 | n |  |  |
| SPBC1773.02c | 0.93 | n |  |  |
| SPBC2F12.03c | 0.93 | n |  |  |
| SPAC27D7.12c | 0.93 | n |  |  |
| SPBC83.16c | 0.93 | n |  |  |
| SPCC1682.06 | 0.93 | n |  |  |
| SPCC1494.08c | 0.93 | n |  |  |
| SPCC1840.08c | 0.93 | n |  |  |
| SPCC594.03 | 0.93 | n |  |  |
| SPBPB2B2.11 | 0.93 | n |  |  |
| SPAC11D3.05 | 0.93 | n |  |  |
| SPAC977.04 | 0.93 | y, n |  |  |
| SPAC11G7.03 | 0.93 |  |  |  |
| SPAC212.01c | 0.93 |  |  |  |
| SPAC1F7.10 | 0.93 |  |  |  |
| SPAC1B3.16c | 0.93 |  |  |  |
| SPCC1919.04 | 0.93 |  |  |  |
| SPBC1778.03c | 0.94 | n |  |  |
| SPAC30D11.14c | 0.94 | n |  |  |
| SPBC1683.04 | 0.94 | n |  |  |
| SPBP4H10.18c | 0.94 | n |  |  |
| SPBP8B7.07c | 0.94 | n |  |  |
| SPCC663.09c | 0.94 | n |  |  |
| SPBC1A4.02c | 0.94 | n |  |  |
| SPAC23C11.14 | 0.94 | n |  |  |
| SPBC15C4.06c | 0.94 | n |  |  |
| SPBC582.10c | 0.94 | y, n |  |  |
| SPBC359.05 | 0.94 |  |  |  |
| SPAC12G12.11c | 0.94 |  |  |  |
| SPAC1F7.06 | 0.94 |  |  |  |
| SPAC4H3.04c | 0.94 |  |  |  |
| SPAC5H10.01 | 0.94 |  |  |  |
| SPBC1778.09 | 0.95 | n |  |  |
| SPBP35G2.06c | 0.95 | n | 3 | n |
| SPBC13E7.11 | 0.95 | n |  |  |
| SPAC3C7.06c | 0.95 | n |  |  |
| SPCC4B3.13 | 0.95 | n |  |  |
| SPAC10F6.11c | 0.95 | n |  |  |
| SPBC119.03 | 0.95 | n |  |  |
| SPAC926.02 | 0.95 | n |  |  |
| SPBC1105.18c | 0.95 | n |  |  |
| SPAC17A5.05c | 0.95 | n |  |  |
| SPBC8D2.16c | 0.95 | n |  |  |
| SPCC24B10.18 | 0.95 | n |  |  |
| SPBC18A7.01 | 0.95 | n |  |  |
| SPAC17D4.04 | 0.95 | n |  |  |
| SPAC6B12.09 | 0.95 | n |  |  |
| SPAC2C4.09 | 0.95 | n |  |  |
| SPBC428.11 | 0.95 | y |  |  |
| SPAC3G9.03(rpl2301) | 0.95 | y |  |  |
| SPBC1198.09(ubc16) | 0.95 | y | 5-/+ | n |
| SPCC576.13(swc5) | 0.95 | y |  |  |
| SPAC31F12.01(zds1) | 0.95 | y, y? |  |  |
| SPAC1093.02 | 0.95 |  |  |  |
| SPAC12B10.16c | 0.95 |  |  |  |
| SPAC222.13c | 0.95 |  |  |  |
| SPAC750.07c | 0.95 |  |  |  |
| SPAC4H3.01 | 0.95 |  |  |  |
| SPAPB8E5.10 | 0.95 |  |  |  |
| SPBC543.02c | 0.96 | n |  |  |
| SPCC736.14 | 0.96 | n |  |  |
| SPAC22A12.14c | 0.96 | n |  |  |
| SPAC1071.12c | 0.96 | n |  |  |
| SPCC11E10.05c | 0.96 | n |  |  |
| SPAC22H10.13 | 0.96 | n |  |  |
| SPAC22H10.08 | 0.96 | n |  |  |
| SPAC24H6.08 | 0.96 | n |  |  |
| SPAC3C7.09 | 0.96 | n |  |  |
| SPBC11C11.08 | 0.96 | n |  |  |
| SPAC2H10.02c | 0.96 | n |  |  |
| SPCC1884.02 | 0.96 | n |  |  |
| SPBC1271.03c | 0.96 | n |  |  |
| SPBC1683.07 | 0.96 | n |  |  |
| SPBC1105.13c | 0.96 | n |  |  |
| SPBC1105.05 | 0.96 | n |  |  |
| SPBC25B2.07c | 0.96 | n |  |  |
| SPCC191.06 | 0.96 | n |  |  |
| SPCC1450.08c | 0.96 | n |  |  |
| SPCC63.13 | 0.96 | n |  |  |
| SPAC11E3.09 | 0.96 | n |  |  |
| SPBC2G2.05 | 0.96 | y, n | 5-/+ | n |
| SPBC3B8.04c | 0.96 |  |  |  |
| SPAC1A6.09c | 0.96 |  |  |  |
| SPAC630.10 | 0.96 |  |  |  |
| SPBC30D10.12c | 0.96 |  |  |  |
| SPAC1142.06 | 0.96 |  |  |  |
| SPCC825.04c | 0.96 |  |  |  |
| SPBC646.06c | 0.96 |  |  |  |
| SPAPB1A10.05 | 0.96 |  |  |  |
| SPBC4B4.08 | 0.97 | n |  |  |
| SPBC215.01 | 0.97 | n |  |  |
| SPAC688.14 | 0.97 | n |  |  |
| SPBC11C11.10 | 0.97 | n |  |  |
| SPAC1002.17c | 0.97 | n |  |  |
| SPAC23A1.04c | 0.97 | n |  |  |
| SPBP8B7.18c | 0.97 | n |  |  |
| SPAC29A4.17c | 0.97 | n |  |  |
| SPAC23C4.05c | 0.97 | n |  |  |
| SPBC119.16c | 0.97 | n |  |  |
| SPBC1604.16c | 0.97 | n |  |  |
| SPBC11C11.02 | 0.97 | n |  |  |
| SPBC947.05c | 0.97 | n |  |  |
| SPBC3D6.15 | 0.97 | n |  |  |
| SPAC323.04 | 0.97 | n |  |  |
| SPAC30.03c | 0.97 | n |  |  |
| SPAC139.06 | 0.97 | n |  |  |
| SPBC365.11 | 0.97 | y |  |  |
| SPAC1142.09 | 0.97 |  |  |  |
| SPAC1296.04 | 0.97 |  |  |  |
| SPAC3G6.07 | 0.97 |  |  |  |
| SPBC651.11c | 0.97 |  |  |  |
| SPAC589.02c | 0.97 |  |  |  |
| SPCC777.02 | 0.98 | n |  |  |
| SPCC1919.11 | 0.98 | n |  |  |
| SPAC1142.03c | 0.98 | n |  |  |
| SPCC1739.03 | 0.98 | n |  |  |
| SPCC965.14c | 0.98 | n |  |  |
| SPAC17G6.03 | 0.98 | n |  |  |
| SPAC15A10.08 | 0.98 | n |  |  |
| SPBC887.17 | 0.98 | n |  |  |
| SPBC1773.06c | 0.98 | n |  |  |
| SPBC2D10.09 | 0.98 | n |  |  |
| SPBC29A3.12 | 0.98 | n |  |  |
| SPBC3H7.14 | 0.98 | n |  |  |
| SPBC776.14 | 0.98 | n |  |  |
| SPBP4H10.12 | 0.98 | n |  |  |
| SPBC8E4.03 | 0.98 | n |  |  |
| SPCC663.17 | 0.98 | n |  |  |
| SPAC3C7.04 | 0.98 | n |  |  |
| SPAC688.06c | 0.98 | n |  |  |
| SPAC23D3.11 | 0.98 | n |  |  |
| SPBP8B7.23 | 0.98 | y |  |  |
| SPCC1020.12c | 0.98 | y, n |  |  |
| SPAC26F1.10c | 0.98 | y, n |  |  |
| SPAC1142.01 | 0.98 |  |  |  |
| SPAC12B10.11 | 0.98 |  |  |  |
| SPAC12B10.15c | 0.98 |  |  |  |
| SPAC1F7.12 | 0.98 |  |  |  |
| SPAC18B11.09c | 0.98 |  |  |  |
| SPAC186.01 | 0.98 |  |  |  |
| SPAC6G9.03c | 0.98 |  |  |  |
| SPCC24B10.20 | 0.98 |  |  |  |
| SPAC56E4.03 | 0.99 | n |  |  |
| SPAC17H9.14c | 0.99 | n |  |  |
| SPAC13A11.05 | 0.99 | n |  |  |
| SPAPB24D3.04c | 0.99 | n |  |  |
| SPAC15A10.05c | 0.99 | n |  |  |
| SPBC14C8.08c | 0.99 | n |  |  |
| SPBC13E7.07 | 0.99 | n |  |  |
| SPBC530.04 | 0.99 | n |  |  |
| SPBC9B6.11c | 0.99 | n |  |  |
| SPBP8B7.26 | 0.99 | n |  |  |
| SPBC18H10.20c | 0.99 | n |  |  |
| SPCC1281.07c | 0.99 | n |  |  |
| SPCC663.13c | 0.99 | n |  |  |
| SPAPB24D3.07c | 0.99 | n |  |  |
| SPAC23G3.02c | 0.99 | n |  |  |
| SPAC23D3.10c | 0.99 | n |  |  |
| SPBC1348.02 | 0.99 | y |  |  |
| SPAC11E3.10 | 0.99 |  |  |  |
| SPAC17H9.12c | 0.99 |  |  |  |
| SPAC22A12.06c | 0.99 |  |  |  |
| SPAC3H8.02 | 0.99 |  |  |  |
| SPAC56E4.07 | 0.99 |  |  |  |
| SPAC4H3.02c | 0.99 |  |  |  |
| SPAC9.08c | 0.99 |  |  |  |
| SPBC31F10.17c | 0.99 |  |  |  |
| SPBC660.05 | 0.99 |  |  |  |
| SPBC23E6.03c | 0.99 |  |  |  |
| SPBC32H8.03 | 1.00 | n |  |  |
| SPBC25B2.03 | 1.00 | n |  |  |
| SPCC1223.11 | 1.00 | n |  |  |
| SPBC13G1.04c | 1.00 | n |  |  |
| SPAC12G12.16c | 1.00 | n |  |  |
| SPCC1682.14 | 1.00 | n |  |  |
| SPAC32A11.03c | 1.00 | n |  |  |
| SPBC21B10.07 | 1.00 | n |  |  |
| SPCC18B5.10c | 1.00 | n |  |  |
| SPCC569.04 | 1.00 | n |  |  |
| SPCC1919.06c | 1.00 | n |  |  |
| SPAC3H1.12c | 1.00 | n |  |  |
| SPBPB2B2.16c | 1.00 | n |  |  |
| SPAC12G12.01c | 1.00 | y |  |  |
| SPAC26H5.08c(bgl2) | 1.00 | y |  |  |
| SPBC29A3.09c | 1.00 | y, n |  |  |
| SPAC11H11.02c | 1.00 |  |  |  |
| SPAC1250.04c | 1.00 |  |  |  |
| SPAC13F5.04c | 1.00 |  |  |  |
| SPAC1834.09 | 1.00 |  |  |  |
| SPAC1D4.05c | 1.00 |  |  |  |
| SPAC4D7.02c | 1.00 |  |  |  |
| SPAC664.13 | 1.00 |  |  |  |
| SPAC16E8.03 | 1.00 |  |  |  |
| SPCC306.07c | 1.00 |  |  |  |
| SPAC3H5.09c | 1.00 |  |  |  |
| SPAC589.03c | 1.00 |  |  |  |
| SPAC16C9.06c | 1.01 | n |  |  |
| SPAC19E9.01c | 1.01 | n |  |  |
| SPAC959.06c | 1.01 | n |  |  |
| SPBC354.09c | 1.01 | n |  |  |
| SPAC22H10.06c | 1.01 | n |  |  |
| SPAC24B11.09 | 1.01 | n |  |  |
| SPAC3C7.13c | 1.01 | n |  |  |
| SPAC9.02c | 1.01 | n |  |  |
| SPBC19C7.05 | 1.01 | n |  |  |
| SPBC2G5.06c | 1.01 | n |  |  |
| SPCC1235.15 | 1.01 | n |  |  |
| SPBC660.07 | 1.01 | n |  |  |
| SPCC162.04c | 1.01 | n |  |  |
| SPCC61.03 | 1.01 | n |  |  |
| SPAC56F8.14c | 1.01 | n |  |  |
| SPBC14F5.10c | 1.01 | n |  |  |
| SPAC1834.03c | 1.01 | n |  |  |
| SPBC29A10.07 | 1.01 | n |  |  |
| SPAC10F6.04 | 1.01 | n |  |  |
| SPBCPT2R1.01c | 1.01 | y |  |  |
| SPBC1348.09 | 1.01 |  |  |  |
| SPAC13D6.01 | 1.01 |  |  |  |
| SPAC1002.01 | 1.01 |  |  |  |
| SPAC19D5.07 | 1.01 |  |  |  |
| SPAC4F10.17 | 1.01 |  |  |  |
| SPAC6G10.03c | 1.01 |  |  |  |
| SPBPJ4664.03 | 1.02 | n |  |  |
| SPAC9E9.08 | 1.02 | n |  |  |
| SPAC144.06 | 1.02 | n |  |  |
| SPCC1620.03 | 1.02 | n |  |  |
| SPBC2A9.07c | 1.02 | n |  |  |
| SPCC285.04 | 1.02 | n |  |  |
| SPCC191.09c | 1.02 | n |  |  |
| SPCC63.03 | 1.02 | n |  |  |
| SPAC20H4.11c | 1.02 | n |  |  |
| SPBC16E9.14c | 1.02 | n |  |  |
| SPBC1347.08c | 1.02 | n |  |  |
| SPCC1442.05c | 1.02 | n |  |  |
| SPAC23A1.02c | 1.02 | n |  |  |
| SPCC18B5.06 | 1.02 |  |  |  |
| SPAC13A11.04c | 1.02 |  |  |  |
| SPAC18G6.01c | 1.02 |  |  |  |
| SPCC4B3.06c | 1.02 |  |  |  |
| SPCC126.06 | 1.03 | n |  |  |
| SPAC1565.03 | 1.03 | n |  |  |
| SPAC24C9.05c | 1.03 | n |  |  |
| SPCC757.10 | 1.03 | n |  |  |
| SPAC20G4.01(caf16) | 1.03 | n |  |  |
| SPBC25H2.09 | 1.03 | n |  |  |
| SPBC649.03 | 1.03 | n |  |  |
| SPCC1223.10c | 1.03 | n |  |  |
| SPBC1685.09 | 1.03 | n |  |  |
| SPCC1235.01 | 1.03 | n |  |  |
| SPCC13B11.04c | 1.03 | n | 3 | n |
| SPBC713.11c | 1.03 | n |  |  |
| SPAC8C9.11 | 1.03 | n |  |  |
| SPAC56E4.06c | 1.03 | n |  |  |
| SPCC1442.11c | 1.03 | y |  |  |
| SPAC3F10.15c | 1.03 | y, n |  |  |
| SPAC1142.02c | 1.03 |  |  |  |
| SPAC1071.03c | 1.03 |  |  |  |
| SPBC1348.04 | 1.03 |  |  |  |
| SPAC1A6.08c | 1.03 |  |  |  |
| SPAC750.06c | 1.03 |  |  |  |
| SPBC365.08c | 1.03 |  |  |  |
| SPBC16G5.09 | 1.03 |  |  |  |
| SPCC4G3.17 | 1.03 |  |  |  |
| SPCC1442.14c | 1.04 | n |  |  |
| SPBC1271.10c | 1.04 | n |  |  |
| SPAC328.10c | 1.04 | n |  |  |
| SPBC1703.14c | 1.04 | n |  |  |
| SPAC17H9.11 | 1.04 | n |  |  |
| SPAC1556.02c | 1.04 | n |  |  |
| SPAC2E1P3.03c | 1.04 | n |  |  |
| SPBC2D10.07c | 1.04 | n |  |  |
| SPAC23C11.01 | 1.04 | n |  |  |
| SPBC16H5.09c | 1.04 | n |  |  |
| SPBC947.04 | 1.04 | n |  |  |
| SPAC977.17 | 1.04 | n |  |  |
| SPBC359.01 | 1.04 | n |  |  |
| SPAC589.10c | 1.04 | n | 5 | n |
| SPAC16.01 | 1.04 | y, n |  |  |
| SPBC557.04 | 1.04 | y, n |  |  |
| SPAC644.09 | 1.04 |  |  |  |
| SPBC2A9.13 | 1.04 |  |  |  |
| SPCP20C8.01c | 1.04 |  |  |  |
| SPCC1620.13 | 1.04 |  |  |  |
| SPAC20G4.03c | 1.05 | n |  |  |
| SPAC22G7.07c | 1.05 | n |  |  |
| SPBC3E7.10 | 1.05 | n |  |  |
| SPAC2C4.10c | 1.05 | n |  |  |
| SPAC29E6.05c | 1.05 | n |  |  |
| SPCC1183.06 | 1.05 | n |  |  |
| SPBC557.05 | 1.05 | n |  |  |
| SPCC1840.05c | 1.05 | n |  |  |
| SPCC2H8.03 | 1.05 | n |  |  |
| SPCC613.03 | 1.05 | n |  |  |
| SPAC1420.04c | 1.05 | n |  |  |
| SPAC6G9.15c | 1.05 | y, n |  |  |
| SPBP35G2.09 | 1.05 |  |  |  |
| SPAC11G7.06c | 1.05 |  |  |  |
| SPAC1B3.11c | 1.05 |  |  |  |
| SPAC977.14c | 1.05 |  |  |  |
| SPCC132.05c | 1.05 |  |  |  |
| SPAC20H4.04 | 1.05 |  |  |  |
| SPAC27D7.06 | 1.06 | n |  |  |
| SPAC27F1.06c | 1.06 | n |  |  |
| SPAC16A10.08c | 1.06 | n |  |  |
| SPAC26A3.17c | 1.06 | n |  |  |
| SPAP8A3.13c | 1.06 | n |  |  |
| SPBC1347.13c | 1.06 | n |  |  |
| SPBC1709.19c | 1.06 | n |  |  |
| SPCC1884.01 | 1.06 | n |  |  |
| SPCC622.04 | 1.06 | n |  |  |
| SPAC1805.06c | 1.06 | n |  |  |
| SPAC23H3.15c | 1.06 | n |  |  |
| SPAC13F5.03c(gld1) | 1.06 | y |  |  |
| SPAC5H10.13c | 1.06 | y, n |  |  |
| SPAC11D3.15 | 1.06 |  |  |  |
| SPAC1F3.08c | 1.06 |  |  |  |
| SPAC637.13c | 1.06 |  |  |  |
| SPAC7D4.13c | 1.06 |  |  |  |
| SPAC8C9.06c | 1.06 |  |  |  |
| SPAC4G8.06c | 1.06 |  |  |  |
| SPBC16G5.07c | 1.06 |  |  |  |
| SPAC23D3.04c | 1.06 |  |  |  |
| SPBC1683.13c | 1.06 |  |  |  |
| SPAC14C4.07 | 1.06 |  |  |  |
| SPCC16A11.15c | 1.06 |  |  |  |
| SPAPB1A10.13 | 1.06 |  |  |  |
| SPAC17C9.09c | 1.07 | n |  |  |
| SPAC23G3.10c | 1.07 | n |  |  |
| SPBC577.06c | 1.07 | n |  |  |
| SPBC29B5.04c | 1.07 | n |  |  |
| SPBC4B4.12c | 1.07 | n |  |  |
| SPCC1919.15 | 1.07 | n |  |  |
| SPCC1281.08 | 1.07 | n |  |  |
| SPCC18.18c | 1.07 | n |  |  |
| SPCC188.09c | 1.07 | n |  |  |
| SPCC1827.03c | 1.07 | n |  |  |
| SPCC576.04 | 1.07 | n |  |  |
| SPCC777.06c | 1.07 | n |  |  |
| SPBC16G5.05c | 1.07 | y, n |  |  |
| SPAC11D3.08c | 1.07 |  |  |  |
| SPAC19B12.09 | 1.07 |  |  |  |
| SPAC5H10.09c | 1.07 |  |  |  |
| SPAC750.03c | 1.07 |  |  |  |
| SPCC965.10 | 1.08 | n |  |  |
| SPBC1734.13 | 1.08 | n |  |  |
| SPAC144.05 | 1.08 | n |  |  |
| SPBC56F2.03 | 1.08 | n |  |  |
| SPAC513.02 | 1.08 | n |  |  |
| SPBC106.03 | 1.08 | n |  |  |
| SPBC342.04 | 1.08 | n |  |  |
| SPBC1347.01c | 1.08 | n |  |  |
| SPCC1906.04 | 1.08 | n |  |  |
| SPCC757.11c | 1.08 | n |  |  |
| SPBC56F2.10c(alg5) | 1.08 | y |  |  |
| SPBPB2B2.12c | 1.08 | y, n |  |  |
| SPBC1348.03 | 1.08 |  |  |  |
| SPAC16E8.14c | 1.08 |  |  |  |
| SPAC17H9.03c | 1.08 |  |  |  |
| SPACUNK4.09 | 1.08 |  |  |  |
| SPCC132.02 | 1.09 | n |  |  |
| SPBC16A3.16 | 1.09 | n |  |  |
| SPBC21B10.04c | 1.09 | n |  |  |
| SPAC30D11.01c | 1.09 | n |  |  |
| SPAC25B8.15c | 1.09 | n |  |  |
| SPBC2G2.07c | 1.09 | n |  |  |
| SPBC20F10.02c | 1.09 | n |  |  |
| SPAC3H8.07c | 1.09 | n |  |  |
| SPAC328.09 | 1.09 | y, n |  |  |
| SPBC342.05 | 1.09 |  |  |  |
| SPAC1039.11c | 1.09 |  |  |  |
| SPAC56F8.05c | 1.09 |  |  |  |
| SPAC5D6.09c | 1.09 |  |  |  |
| SPAC5H10.07 | 1.09 |  |  |  |
| SPBC1539.07c | 1.09 |  |  |  |
| SPAC3G6.04 | 1.10 | n |  |  |
| SPCC1020.09 | 1.10 | n |  |  |
| SPBC30B4.08 | 1.10 | n |  |  |
| SPBC16C6.03c | 1.10 | n |  |  |
| SPBPB10D8.02c | 1.10 | n |  |  |
| SPCC622.06c | 1.10 | n |  |  |
| SPCC11E10.07c | 1.10 | n |  |  |
| SPBPB2B2.03c | 1.10 | n |  |  |
| SPAC2H10.01 | 1.10 | n |  |  |
| SPAC22E12.19 | 1.10 | n |  |  |
| SPBC11G11.01(fis1) | 1.10 | y |  |  |
| SPCC126.03 | 1.10 | y, n | 5-/+ | n |
| SPAC1952.06c | 1.10 |  |  |  |
| SPAC8F11.05c | 1.10 |  |  |  |
| SPAC8C9.09c | 1.10 |  |  |  |
| SPBC2D10.19c | 1.10 |  |  |  |
| SPCC1322.11 | 1.11 | n |  |  |
| SPAC4F8.10c | 1.11 | n |  |  |
| SPAC3F10.18c | 1.11 | n |  |  |
| SPBC2A9.10 | 1.11 | n |  |  |
| SPCC132.01c | 1.11 | n |  |  |
| SPBPB10D8.04c | 1.11 | n |  |  |
| SPCC550.08 | 1.11 | n |  |  |
| SPCC794.09c | 1.11 | n |  |  |
| SPCC830.02 | 1.11 | n |  |  |
| SPCC24B10.17 | 1.11 | n |  |  |
| SPCC1840.12 | 1.11 | n |  |  |
| SPAC3A11.10c | 1.11 | n |  |  |
| SPAC7D4.08 | 1.11 | n |  |  |
| SPAC12G12.10 | 1.11 |  |  |  |
| SPAC16.04 | 1.11 |  |  |  |
| SPAC1A6.06c | 1.11 |  |  |  |
| SPBC26H8.01 | 1.12 | n |  |  |
| SPCC285.16c | 1.12 | n |  |  |
| SPBC1773.17c | 1.12 | n |  |  |
| SPBC31F10.02 | 1.12 | n |  |  |
| SPBC1198.03c | 1.12 | n |  |  |
| SPCC794.02 | 1.12 | n |  |  |
| SPAC23C4.09c | 1.12 | y |  |  |
| SPAPJ695.01c | 1.12 | y |  |  |
| SPBC1652.02 | 1.12 |  |  |  |
| SPAC19G12.09 | 1.12 |  |  |  |
| SPAC8C9.16c | 1.12 |  |  |  |
| SPAC926.05c | 1.12 |  |  |  |
| SPAC2E1P3.02c | 1.12 |  |  |  |
| SPAC1687.15 | 1.13 | n |  |  |
| SPBC725.05c | 1.13 | n |  |  |
| SPAC23H4.10c | 1.13 | n |  |  |
| SPAC806.07 | 1.13 | n |  |  |
| SPCC1840.07c | 1.13 | n |  |  |
| SPAC25B8.18 | 1.13 | n |  |  |
| SPAC15E1.09 | 1.13 | n |  |  |
| SPAC750.02c | 1.13 | n |  |  |
| SPBC1348.01 | 1.13 | n |  |  |
| SPAC26F1.01 | 1.13 | n |  |  |
| SPBPB10D8.03 | 1.13 | n |  |  |
| SPAC29B12.10c(pgt1) | 1.13 | y |  |  |
| SPAC12D12.09 | 1.13 |  |  |  |
| SPBC337.07c | 1.13 |  |  |  |
| SPBC651.12c | 1.13 |  |  |  |
| SPAC1F8.03c | 1.13 |  |  |  |
| SPAC23E2.03c | 1.14 | n |  |  |
| SPAC1834.04 | 1.14 | n |  |  |
| SPAC25B8.17 | 1.14 | n |  |  |
| SPAC25H1.06 | 1.14 | n |  |  |
| SPAPJ691.03 | 1.14 | n |  |  |
| SPBC106.11c | 1.14 | n |  |  |
| SPBC405.06 | 1.14 | n |  |  |
| SPCC1795.12c | 1.14 | n |  |  |
| SPCC594.07c | 1.14 | n |  |  |
| SPCC736.13 | 1.14 | n |  |  |
| SPCC737.07c | 1.14 | n |  |  |
| SPAC212.09c | 1.14 | n |  |  |
| SPBC12C2.04 | 1.14 | n |  |  |
| SPCC1739.10(mug33) | 1.14 | y |  |  |
| SPAC1006.01 | 1.14 | y, n |  |  |
| SPBC776.11 | 1.14 | y, n | 5-/+ | n |
| SPAC1071.02 | 1.14 | y, n |  |  |
| SPBC2D10.05 | 1.14 | y, n |  |  |
| SPAC1039.02 | 1.14 |  |  |  |
| SPAC630.06c | 1.14 |  |  |  |
| SPAC607.08c | 1.14 |  |  |  |
| SPAP27G11.12 | 1.14 |  |  |  |
| SPAC1952.16 | 1.15 | n |  |  |
| SPAC140.04 | 1.15 | n |  |  |
| SPAC3G9.07c | 1.15 | n |  |  |
| SPAC3A11.06 | 1.15 | n |  |  |
| SPBC15D4.12c | 1.15 | n |  |  |
| SPBC2A9.05c | 1.15 | n |  |  |
| SPBP22H7.06 | 1.15 | n |  |  |
| SPCC23B6.02c | 1.15 | n |  |  |
| SPAC17A2.05 | 1.15 | n |  |  |
| SPAC23D3.12 | 1.15 |  |  |  |
| SPAC4H3.06 | 1.15 |  |  |  |
| SPAC3G6.05 | 1.15 |  |  |  |
| SPAP8A3.12c | 1.15 |  |  |  |
| SPBC27B12.14 | 1.15 |  |  |  |
| SPAC24H6.13 | 1.15 |  |  |  |
| SPAPB1A11.02 | 1.15 |  |  |  |
| SPCC63.06 | 1.15 |  |  |  |
| SPCC70.06 | 1.16 | n |  |  |
| SPAC1002.14 | 1.16 | n |  |  |
| SPAC24H6.11c | 1.16 | n |  |  |
| SPAC20G4.04c | 1.16 | n |  |  |
| SPBC17A3.02 | 1.16 | n |  |  |
| SPCC1672.09 | 1.16 | n |  |  |
| SPCC18.09c | 1.16 | n |  |  |
| SPCC417.04 | 1.16 | n |  |  |
| SPCC417.03 | 1.16 | n |  |  |
| SPBC1539.10 | 1.16 | n |  |  |
| SPAC4H3.12c | 1.16 | y |  |  |
| SPCC4B3.12 | 1.16 |  |  |  |
| SPAC18B11.02c | 1.16 |  |  |  |
| SPAC17G6.02c | 1.17 | n |  |  |
| SPAC9G1.06c | 1.17 | n |  |  |
| SPBC1685.14c | 1.17 | n |  |  |
| SPBC405.02c | 1.17 | n |  |  |
| SPBC215.13 | 1.17 | n |  |  |
| SPBP35G2.12 | 1.17 | n |  |  |
| SPBC11B10.08 | 1.17 | n |  |  |
| SPAC4G8.08 | 1.17 | n |  |  |
| SPCC1442.04c | 1.17 | y |  |  |
| SPAC1002.18 | 1.17 |  |  |  |
| SPAC1B3.02c | 1.17 |  |  |  |
| SPAC750.08c | 1.17 |  |  |  |
| SPAC664.14 | 1.17 |  |  |  |
| SPAC328.06 | 1.18 | n |  |  |
| SPCC18.03 | 1.18 | n |  |  |
| SPAC24C9.16c | 1.18 | n |  |  |
| SPBC609.04 | 1.18 | n |  |  |
| SPAC1952.11c | 1.18 | n |  |  |
| SPBC29A3.10c | 1.18 | n |  |  |
| SPBC30D10.05c | 1.18 | n |  |  |
| SPBP35G2.04c | 1.18 | n |  |  |
| SPBC19C2.04c | 1.18 | n |  |  |
| SPAC167.04(pam17) | 1.18 | y |  |  |
| SPAC105.03c | 1.18 | y, n |  |  |
| SPBC354.08c | 1.18 | y, n |  |  |
| SPBC32F12.12c | 1.18 | y, n |  |  |
| SPAC1834.12 | 1.18 |  |  |  |
| SPAC1039.09 | 1.18 |  |  |  |
| SPAC17G8.11c | 1.18 |  |  |  |
| SPAC56F8.02 | 1.18 |  |  |  |
| SPBC12D12.02c | 1.18 |  |  |  |
| SPAC30.04c | 1.19 | n |  |  |
| SPAC4A8.06c | 1.19 | n |  |  |
| SPAP27G11.15 | 1.19 | n |  |  |
| SPBC215.11c | 1.19 | n |  |  |
| SPBC902.05c | 1.19 | n |  |  |
| SPBP4G3.03 | 1.19 | n |  |  |
| SPBP26C9.02c | 1.19 | n |  |  |
| SPCC1020.13c | 1.19 | n |  |  |
| SPAC4F8.15 | 1.19 | n |  |  |
| SPBC8D2.11 | 1.19 | n |  |  |
| SPBC1348.11 | 1.19 |  |  |  |
| SPAC19D5.02c | 1.19 |  |  |  |
| SPAC732.02c | 1.19 |  |  |  |
| SPCC330.11 | 1.19 |  |  |  |
| SPCC63.14 | 1.20 | n |  |  |
| SPCC1840.12 | 1.20 | n |  |  |
| SPAC9E9.01 | 1.20 | n |  |  |
| SPBC354.13(rga6) | 1.20 | y |  |  |
| SPAC19B12.12c | 1.20 |  |  |  |
| SPCC5E4.05c | 1.20 |  |  |  |
| SPAC1B2.04 | 1.21 | n |  |  |
| SPAC2C4.06c | 1.21 | n |  |  |
| SPBC215.10 | 1.21 | n |  |  |
| SPCC1259.09c | 1.21 | n |  |  |
| SPBC119.14 | 1.21 | n |  |  |
| SPCC16C4.13c | 1.21 | n |  |  |
| SPBC36B7.08c | 1.21 | y |  |  |
| SPAC12G12.09 | 1.21 |  |  |  |
| SPCC1223.01 | 1.22 | n |  |  |
| SPAC1687.22c | 1.22 | n |  |  |
| SPAC139.01c | 1.22 | n |  |  |
| SPAC17G8.02 | 1.22 | n |  |  |
| SPAC22E12.03c | 1.22 | n |  |  |
| SPAC26H5.09c | 1.22 | n |  |  |
| SPCC584.13 | 1.22 | n |  |  |
| SPBC1604.07 | 1.22 | n |  |  |
| SPCC1620.02 | 1.22 | n |  |  |
| SPCC584.02 | 1.22 | n |  |  |
| SPBC409.17c | 1.22 | n |  |  |
| SPAC26F1.11 | 1.22 | n |  |  |
| SPBC17D11.02c | 1.22 | y, n |  |  |
| SPAC11D3.14c | 1.22 |  |  |  |
| SPAC1B3.06c | 1.22 |  |  |  |
| SPAC5D6.07c | 1.22 |  |  |  |
| SPAC6G9.01c | 1.22 |  |  |  |
| SPAC4H3.03c | 1.22 |  |  |  |
| SPAP8A3.02c | 1.22 |  |  |  |
| SPAC27D7.08c | 1.23 | n |  |  |
| SPBC776.16 | 1.23 | n |  |  |
| SPAPB24D3.02c | 1.23 | n |  |  |
| SPAC1783.01 | 1.23 |  |  |  |
| SPAC186.05c | 1.23 |  |  |  |
| SPAC18B11.08c | 1.23 |  |  |  |
| SPAC6G9.09c | 1.23 |  |  |  |
| SPAC6C3.03c | 1.23 |  |  |  |
| SPAC2G11.12 | 1.24 | n |  |  |
| SPAC13G6.08 | 1.24 | n |  |  |
| SPBC2D10.20 | 1.24 | n |  |  |
| SPBC16D10.01c | 1.24 | n |  |  |
| SPCC320.14 | 1.24 | n |  |  |
| SPAC694.04c | 1.24 | n |  |  |
| SPCC970.05(rpl3601) | 1.24 | y |  |  |
| SPAC212.08c | 1.24 |  |  |  |
| SPAC17A5.09c | 1.24 |  |  |  |
| SPAC8F11.02c | 1.24 |  |  |  |
| SPCC1450.07c | 1.25 | n |  |  |
| SPAC14C4.13 | 1.25 | n | 5 | n |
| SPBC19C2.13c | 1.25 | n |  |  |
| SPBC4C3.06 | 1.25 | n |  |  |
| SPAC227.03c | 1.25 | n |  |  |
| SPBC21C3.08c | 1.25 | n |  |  |
| SPBC216.05 | 1.25 |  |  |  |
| SPAC11D3.10 | 1.25 |  |  |  |
| SPBC839.06 | 1.26 | n |  |  |
| SPAC15A10.13 | 1.26 | n |  |  |
| SPCC1739.05 | 1.26 | n |  |  |
| SPBC3H7.08c | 1.26 | n |  |  |
| SPBC543.08 | 1.26 | n |  |  |
| SPAC1782.08c(rex3) | 1.26 | y |  |  |
| SPAC1A6.05c | 1.26 |  |  |  |
| SPAC644.13c | 1.26 |  |  |  |
| SPAC11D3.07c | 1.27 | n | 3 | n |
| SPAC869.03c | 1.27 | n |  |  |
| SPBC1347.09 | 1.27 | n |  |  |
| SPAC323.07c | 1.27 | n |  |  |
| SPBC3B9.05 | 1.27 | y |  |  |
| SPAC3G9.16c(bet5) | 1.27 | y |  |  |
| SPAC4F8.08 | 1.27 |  |  |  |
| SPAC9G1.05 | 1.28 | n |  |  |
| SPAC14C4.04 | 1.28 | n |  |  |
| SPBC31F10.03 | 1.28 | n |  |  |
| SPAC22H10.11c | 1.28 | y |  |  |
| SPAC30D11.12 | 1.29 | n |  |  |
| SPCC1840.09 | 1.29 | n |  |  |
| SPCC1620.08 | 1.29 | n |  |  |
| SPCC285.17(spp27) | 1.29 | y |  |  |
| SPAC19A8.02 | 1.29 |  |  |  |
| SPAC4G8.07c | 1.29 |  |  |  |
| SPAC9E9.13 | 1.30 | n |  |  |
| SPBC12C2.07c | 1.30 | n |  |  |
| SPBC19G7.01c | 1.30 | n |  |  |
| SPCC1682.11c | 1.30 | n |  |  |
| SPCC2H8.05c | 1.30 | n |  |  |
| SPAC7D4.12c | 1.30 | y |  |  |
| SPBC530.08 | 1.30 | y, n |  |  |
| SPBC1348.05 | 1.30 |  |  |  |
| SPBC21C3.03 | 1.31 | n |  |  |
| SPAC30C2.08 | 1.31 | n |  |  |
| SPAC323.05c | 1.31 | n |  |  |
| SPCC622.02 | 1.31 | n | 5-/+ | ± |
| SPBPB2B2.17c | 1.31 | n |  |  |
| SPAC3F10.13 | 1.31 | y, n |  |  |
| SPAC12B10.04 | 1.31 |  |  |  |
| SPAC11H11.03c | 1.31 |  |  |  |
| SPAC6B12.06c | 1.31 |  |  |  |
| SPAC13G6.07c | 1.32 | n |  |  |
| SPAC13G6.02c | 1.32 | n |  |  |
| SPBC1348.07 | 1.32 | n |  |  |
| SPBC1711.15c | 1.32 | n |  |  |
| SPAC11E3.03 | 1.32 |  |  |  |
| SPCC777.03c | 1.32 |  |  |  |
| SPCC126.11c | 1.33 | n |  |  |
| SPAC17C9.12 | 1.33 | n |  |  |
| SPBC1703.13c | 1.33 |  |  |  |
| SPAC977.16c | 1.33 |  |  |  |
| SPAC30D11.06c | 1.34 | n |  |  |
| SPBC36.11 | 1.34 | n |  |  |
| SPCC24B10.16c | 1.34 | n |  |  |
| SPAC13D1.01c | 1.34 | n |  |  |
| SPAC3G6.09c | 1.34 |  |  |  |
| SPBC359.03c | 1.34 |  |  |  |
| SPCPB16A4.06c | 1.35 | n |  |  |
| SPAC10F6.07c | 1.35 |  |  |  |
| SPCC16A11.03c | 1.35 |  |  |  |
| SPCC548.05c | 1.36 | n |  |  |
| SPBC3F6.01c | 1.36 | n |  |  |
| SPBC14C8.05c | 1.36 | n |  |  |
| SPCC70.03c | 1.36 | n |  |  |
| SPAC1D4.01 | 1.36 |  |  |  |
| SPAC19G12.03 | 1.36 |  |  |  |
| SPBC16G5.17 | 1.37 | n |  |  |
| SPAC19A8.10 | 1.37 | n |  |  |
| SPBC15C4.04c | 1.37 | n |  |  |
| SPCC965.13 | 1.37 | n |  |  |
| SPCC895.05 | 1.37 |  |  |  |
| SPAC1071.11 | 1.38 | n |  |  |
| SPAC5D6.13 | 1.38 |  |  |  |
| SPAC22F3.03c | 1.39 | n |  |  |
| SPCC126.09 | 1.39 | n |  |  |
| SPAC56F8.15 | 1.39 |  |  |  |
| SPAC1071.05 | 1.40 |  |  |  |
| SPBC21D10.08c | 1.42 | n |  |  |
| SPAC19D5.09c | 1.42 | n |  |  |
| SPCC550.09 | 1.42 |  |  |  |
| SPBC1683.08 | 1.43 | n |  |  |
| SPAC19G12.02c | 1.43 | n |  |  |
| SPCC74.06 | 1.43 | n |  |  |
| SPBP8B7.08c | 1.44 | y, n |  |  |
| SPAC13D6.04c | 1.45 | n |  |  |
| SPBC1685.07c | 1.45 | n |  |  |
| SPBC8E4.01c | 1.45 | n |  |  |
| SPBC1604.11 | 1.45 |  |  |  |
| SPBC409.08 | 1.45 |  |  |  |
| SPAC2F3.11 | 1.46 | n |  |  |
| SPAC977.11 | 1.46 | n |  |  |
| SPACUNK4.17 | 1.46 |  |  |  |
| SPBC1703.11 | 1.47 | n |  |  |
| SPAC6C3.09 | 1.47 |  |  |  |
| SPCC24B10.15 | 1.48 | n |  |  |
| SPBC1683.10c | 1.48 | n |  |  |
| SPAP32A8.02 | 1.48 | n |  |  |
| SPBC21D10.09c | 1.49 | n |  |  |
| SPAC14C4.09 | 1.51 | n |  |  |
| SPAC2C4.15c | 1.51 | n |  |  |
| SPBC17D1.02 | 1.51 | y, n |  |  |
| SPAC458.02c | 1.52 |  |  |  |
| SPAC17D4.04 | 1.52 |  |  |  |
| SPAC22A12.17c | 1.53 |  |  |  |
| SPCC1281.03c | 1.54 | n |  |  |
| SPAC22H12.03 | 1.54 | y |  |  |
| SPAC1952.12c | 1.54 |  |  |  |
| SPCC965.06 | 1.55 | n |  |  |
| SPBPJ4664.01 | 1.55 | n |  |  |
| SPBC17A3.08 | 1.55 | n |  |  |
| SPAC13G6.06c | 1.56 | n |  |  |
| SPAC3G6.13c | 1.56 |  |  |  |
| SPBC21D10.07 | 1.57 | n |  |  |
| SPBC21B10.10 | 1.58 | n |  |  |
| SPAC664.15(caf4) | 1.58 | n |  |  |
| SPAC27D7.09c | 1.59 | n |  |  |
| SPBC1348.13 | 1.59 |  |  |  |
| SPCC1020.10 | 1.60 | y, n |  |  |
| SPAC26F1.08c | 1.61 | n |  |  |
| SPAC513.04 | 1.62 |  |  |  |
| SPAC20H4.02 | 1.63 | n |  |  |
| SPBP8B7.06 | 1.67 | n |  |  |
| SPBC13G1.13 | 1.69 | n |  |  |
| SPAC13A11.06 | 1.70 |  |  |  |
| SPAC1834.08 | 1.71 |  |  |  |
| SPCC1450.06c(grx3) | 1.72 | y |  |  |
| SPAC25B8.11 | 1.74 | n |  |  |
| SPCC663.15c | 1.74 | n |  |  |
| SPBC1271.07c | 1.75 | n |  |  |
| SPCC74.05 | 1.76 | n |  |  |
| SPCC63.04 | 1.79 | n |  |  |
| SPBC16D10.05 | 1.89 | y, n |  |  |
| SPCC11E10.03 | 1.94 | n |  |  |
| SPAC29A4.05 | 2.11 | n |  |  |
| SPBC354.10 | 2.39 | n |  |  |
| SPAC1D4.02c | 2.49 | n |  |  |
| SPAC19D5.01 | 2.89 | y, n |  |  |
| SPBC13G1.08c(ash2) | 5.00 |  |  |  |
| SPCC18.13 | 0.08, 0.19 | n |  |  |
| SPAC11E3.11c | 0.08, 0.2 | n |  |  |
| SPBC15D4.10c(amo1) | 0.11, 0.11 | y, n, n | 5± | + |
| SPAC9.07c | 0.13, 0.08 | n |  |  |
| SPAC15E1.06(vps29) | 0.13, 0.24 | y, n, n |  |  |
| SPAC23C4.08(rho3) | 0.14, 0.10 | n |  |  |
| SPBC12C2.03c | 0.14, 0.25 | y, n, n |  |  |
| SPCC1259.11c(gyp2) | 0.17, 0.50 | n |  |  |
| SPCPB16A4.04c | 0.17, 0.83 | n |  |  |
| SPBC9B6.07 | 0.18, 0.80 | y, n |  |  |
| SPBC337.02c | 0.18, 1.24 | n |  |  |
| SPBC947.15c | 0.20, 0.32 | n, n |  |  |
| SPAC17G8.07(yaf9) | 0.20, 0.43 | n | 1 | ++ |
| SPAPYUK71.03c(syn1) | 0.20, 0.63 | n |  |  |
| SPAC11H11.01(sst6) | 0.21, 0.80 | n |  |  |
| SPAC664.02c(arp8) | 0.22, 0.31 | y, n |  |  |
| SPCC1827.04 | 0.22, 0.36 | n, n |  |  |
| SPCC550.03c | 0.23, 0.42 | n |  |  |
| SPCC18.10 | 0.25, 0.43 | n |  |  |
| SPCC965.11c | 0.26, 0.06 | n |  |  |
| SPAC4F10.14c(btf3) | 0.27, 0.34 | y, y |  |  |
| SPAC8F11.10c(pvg1) | 0.28, 0.38 | y, n, n |  |  |
| SPAP7G5.05(rpl1002) | 0.28, 0.64 | n |  |  |
| SPBC31F10.05 | 0.28, 0.92 | n |  |  |
| SPAC1782.09c(clp1) | 0.29, 0.19 | y, y | 5 | n |
| SPBC13E7.09(vrp1) | 0.29, 0.29 | n |  |  |
| SPAC521.04c | 0.31, 0.44 | y, n |  |  |
| SPAC27D7.04 | 0.32, 0.80 |  |  |  |
| SPAC664.01c(swi6) | 0.33, 0.14 | y, y |  |  |
| SPAC821.05 | 0.33, 0.25 | y, n, n | 2 | ± |
| SPAC17C9.10(stm1) | 0.33, 0.30 | n, n |  |  |
| SPAPB2B4.04c | 0.33, 0.80 |  |  |  |
| SPAC1071.06(arp9) | 0.34, 0.29 | n, n |  |  |
| SPBC25H2.10c | 0.34, 0.51 | n |  |  |
| SPAC29B12.11c | 0.36, 1.29 | n |  |  |
| SPCC1672.06c(asp1) | 0.38, 0.28 | y, n |  |  |
| SPAC4F10.16c | 0.38, 0.66 | n |  |  |
| SPBC8D2.02c | 0.39, 0.42 | n |  |  |
| SPCP31B10.06(mug190) | 0.40, 0.41 | n |  |  |
| SPBC1921.04c | 0.40, 0.66 | n |  |  |
| SPAC1B3.05(not3) | 0.41, 0.28 | y, y |  |  |
| SPBC17D11.08 | 0.41, 0.32 | n, n |  |  |
| SPBC530.01(gyp1) | 0.43, 0.52 | y, n |  |  |
| SPCC1494.07 | 0.43, 0.55 | n |  |  |
| SPAC17A5.16(ftp105) | 0.44, 0.52 |  |  |  |
| SPAC4D7.06c | 0.44, 0.58 |  |  |  |
| SPAC343.10 | 0.44, 0.60 | n |  |  |
| SPBC725.09c(hob3) | 0.45, 0.29 | n, n |  |  |
| SPAC1B1.04c | 0.45, 0.46 | n, y |  |  |
| SPAC17A2.11 | 0.46, 0.62 | n |  |  |
| SPAC1039.05c | 0.46, 0.72 | n |  |  |
| SPAC922.03 | 0.46, 0.90 | n |  |  |
| SPCC188.12 | 0.46, 0.95 | n |  |  |
| SPCC1322.06(kap113) | 0.47, 0.64 | y |  |  |
| SPBC365.07c | 0.47, 1.22 | n |  |  |
| SPAC144.02(iec1) | 0.48, 0.32 | y, n |  |  |
| SPAC15A10.15(sgo2) | 0.48, 0.55 |  |  |  |
| SPAC56F8.08 | 0.48, 0.71 |  |  |  |
| SPBC21C3.02c | 0.48, 0.78 | n |  |  |
| SPBC725.12(nbl1) | 0.49, 0.50 | n, n |  |  |
| SPBC947.08c | 0.49, 0.60 | n |  |  |
| SPCC1795.10c | 0.49, 0.61 | n |  |  |
| SPACUNK4.15 | 0.49, 0.65 |  |  |  |
| SPAC3G6.11 | 0.49, 0.79 | n |  |  |
| SPBC19C2.10 | 0.49, 0.80 | n |  |  |
| SPAC26F1.02 | 0.49, 1.04 | n |  |  |
| SPBC15D4.15 | 0.50, 0.85 | n |  |  |
| SPBC336.14c(ppk26) | 0.53, 0.50 | n, y |  |  |
| SPCC1223.06 | 0.53, 0.59 | n |  |  |
| SPCC970.07c(raf2) | 0.54, 0.40 | y, n |  |  |
| SPAC3G9.04(ssu72) | 0.54, 0.42 | n |  |  |
| SPAC631.01c | 0.60, 0.53 | n |  |  |
| SPBC18H10.06c | 0.61, 0.52 | n |  |  |
| SPCC1259.03(rpa12) | 0.61, 0.80 | y |  |  |
| SPCC794.15 | 0.63, 0.58 | n |  |  |
| SPBC839.03c | 0.64, 1.22 | n |  |  |
| SPAC19B12.04 | 0.65, 0.67 | y, n |  |  |
| SPCC794.01c | 0.65, 0.76 | y |  |  |
| SPBC3E7.08c(rad13) | 0.67, 0.26 | n |  |  |
| SPAC6C3.06c | 0.72, 0.89 | n |  |  |
| SPAC19G12.10c | 0.73, 0.73 | n |  |  |
| SPCC1919.05 | 0.73, 0.79 | n |  |  |
| SPBC887.02 | 0.73, 1.13 | y, n |  |  |
| SPAC823.03 | 0.75, 0.73 | n |  |  |
| SPCC18.04 | 0.78, 0.65 | n |  |  |
| SPAC343.07 | 0.78, 0.82 | y, n |  |  |
| SPBC18H10.16 | 0.79, 0.93 | y, n |  |  |
| SPAC1687.13c | 0.81, 1.17 |  |  |  |
| SPAC9.05 | 0.91, 0.56 | n |  |  |
| SPAC4G9.05 | 0.91, 1.01 | n |  |  |
| SPBP8B7.04 | 0.92, 1.01 | n |  |  |
| SPBC25H2.08c | 0.94, 0.63 | y, n |  |  |
| SPAC1527.01 | 0.94, 0.79 | n |  |  |
| SPAC17A5.04c | 0.94, 0.98 | n |  |  |
| SPAC11E3.01c(swr1) | 0.95, 1.17 | y |  |  |
| SPCC16C4.06c | 0.98, 0.69 | n |  |  |
| SPBC17A3.10 | 1.01, 0.77 | n |  |  |
| SPAC212.02 | 1.04, 1.26 | n |  |  |
| SPCC1682.15 | 1.04, 1.32 | y, n |  |  |
| SPAC343.15 | 1.05, 0.58 | n |  |  |
| SPAC17A2.12 | 1.05, 0.60 | n |  |  |
| SPCC24B10.22(pog1) | 1.10, 0.75 | y |  |  |
| SPBC13G1.10c | 1.11, 0.88 | n |  |  |
| SPBC1685.05 | 1.12, 0.69 | n |  |  |
| SPCC548.07c | 1.15, 0.68 | n |  |  |
| SPAC16A10.02 | 1.15, 1.39 | n |  |  |
| SPBPB2B2.05 | 1.16, 0.41 | n |  |  |
| SPAC16A10.07c | 1.16, 1.29 | n |  |  |
| SPBC337.03 | 1.22, 1.03 | y, n |  |  |
| SPCC1020.07 | 1.25, 0.95 | n |  |  |
| SPCC285.15c | 1.25, 1.56 | n |  |  |
| SPBC9B6.03 | 1.34, 1.47 | y |  |  |
| SPBC3H7.13 | 1.37, 1.22 | n |  |  |
| SPBC31A8.01c(rtn1) | 1.48, 1.16 | y, y |  |  |
| SPCC23B6.03c | 1.58, 0.89 | n |  |  |
| SPBC1921.03c(mex67) |  | y, n |  |  |
| SPAC19B12.10 |  | n |  |  |
| SPCC74.04 |  | n |  |  |
| SPCC794.03 |  | n |  |  |
| SPCC777.04 |  | n |  |  |
| SPCC757.04 |  | n |  |  |
| SPAC4D7.03 |  | n | 4 | n |
| SPACUNK4.13c |  | n |  |  |
| SPCC297.05 |  | n |  |  |
| SPAC13D6.02c |  | n |  |  |
| SPAC1635.01 |  | n |  |  |
| SPAC869.04 |  | n |  |  |
| SPBC31F10.13c |  | n |  |  |
| SPCC1322.01 |  | n |  |  |
| SPBC16A3.17c |  | n |  |  |
| SPBC16H5.08c |  | n | 1± | + |
| SPBC577.03c |  | n |  |  |
| SPBC713.03 |  | n |  |  |
| SPAC24H6.09 |  | n |  |  |
| SPBP16F5.08c |  | n |  |  |
| SPBPB2B2.06c |  | n |  |  |
| SPAC6F6.01 |  | n |  |  |
| SPBC543.05c |  | n |  |  |
| SPAC977.15 |  | n |  |  |
| SPAP32A8.03c |  | n |  |  |
| SPAP8A3.14c |  | n |  |  |
| SPAC11E3.08c |  | n |  |  |
| SPBC13E7.04 |  | n |  |  |
| SPBC1215.01 |  | n |  |  |
| SPAC13C5.03 |  | n |  |  |
| SPAC17A5.11 |  | n |  |  |
| SPBC32F12.02 |  | n |  |  |
| SPAC630.13c |  | n |  |  |
| SPAC1296.03c |  | n |  |  |
| SPAC22H10.09 |  | n |  |  |
| SPBC21C3.18 |  | n |  |  |
| SPCC417.06c |  | n |  |  |
| SPBC16D10.07c |  | n |  |  |
| SPBP23A10.02 |  | n |  |  |
| SPBC543.10 |  | n |  |  |
| SPCC1223.12c |  | n |  |  |
| SPBP19A11.02c |  | n |  |  |
| SPBP35G2.11c |  | n |  |  |
| SPBC337.04 |  | n |  |  |
| SPBC32H8.11 |  | n | 5-/+ | n |
| SPAC1D4.06c |  | n |  |  |
| SPBC6B1.02 |  | n |  |  |
| SPAC4G8.05 |  | n |  |  |
| SPAC22E12.14c |  | n |  |  |
| SPAC20G4.02c |  | n |  |  |
| SPAC110.01 |  | n |  |  |
| SPAC17G6.05c |  | n |  |  |
| SPAC19B12.07c |  | n |  |  |
| SPBC216.04c |  | n |  |  |
| SPAC2F7.08c |  | n |  |  |
| SPBP8B7.30c |  | n |  |  |
| SPBC215.06c |  | n |  |  |
| SPAC16.05c |  | n |  |  |
| SPBC1718.02 |  | n |  |  |
| SPAC139.03 |  | n | 3 | n |
| SPAC11H11.04 |  | n |  |  |
| SPAC2G11.07c |  | n |  |  |
| SPCC1919.10c |  | n |  |  |
| SPCC18B5.11c |  | n |  |  |
| SPBC428.03c |  | n |  |  |
| SPAC1783.06c |  | n |  |  |
| SPAC4G8.11c |  | n |  |  |
| SPBC23E6.08 |  | n |  |  |
| SPAC31G5.09c |  | n |  |  |
| SPAC10F6.06 |  | n |  |  |
| SPBC11B10.07c |  | n |  |  |
| SPAC56F8.04c |  | n |  |  |
| SPBC1709.09 |  | n |  |  |
| SPBC2F12.11c |  | n |  |  |
| SPAC19A8.04 |  | n |  |  |
| SPAC869.02c |  | n |  |  |
| SPBC21B10.03c |  | n |  |  |
| SPBC29A3.07c |  | n |  |  |
| SPAC56F8.09 |  | n |  |  |
| SPAC3H8.09c |  | n |  |  |
| SPAC823.10c |  | n |  |  |
| SPBC14F5.13c |  | n |  |  |
| SPAC1610.02c |  | n |  |  |
| SPAC1805.09c |  | n |  |  |
| SPBC18H10.13 |  | n |  |  |
| SPAC694.05c |  | n |  |  |
| SPCC1393.03 |  | n |  |  |
| SPCC777.17c |  | n |  |  |
| SPBC17G9.07 |  | n |  |  |
| SPAC3H8.10 |  | n |  |  |
| SPBC106.02c |  | n |  |  |
| SPBC947.06c |  | n |  |  |
| SPAC22E12.11c |  | n |  |  |
| SPBC1778.04 |  | n |  |  |
| SPBC337.09 |  | n |  |  |
| SPAC6G10.12c |  | n |  |  |
| SPAC18B11.10 |  | n | 6 | n |
| SPBC1861.07 |  | n |  |  |
| SPBC1773.03c |  | n |  |  |
| SPBC1105.11c |  | n |  |  |
| SPBC83.05 |  | n |  |  |
| SPAC105.02c |  | n |  |  |
| SPBC13G1.14c |  | n |  |  |
| SPBC29A10.02 |  | n |  |  |
| SPBC16E9.17c |  | n |  |  |
| SPCC1223.02 |  | n |  |  |
| SPAC9G1.03c |  | n |  |  |
| SPAC25B8.05 |  | n | 6 | n |
| SPAC23G3.05c |  | n |  |  |
| SPAC13G6.14 |  | n |  |  |
| SPAC222.08c |  | n |  |  |
| SPCC364.03 |  | n |  |  |
| SPBC11B10.02c |  | n |  |  |
| SPCC31H12.04c |  | n |  |  |
| SPAC5H10.11 |  | n |  |  |
| SPBC17G9.09 |  | n |  |  |
| SPCC338.14 |  | n |  |  |
| SPAC29A4.20 |  | n |  |  |
| SPBC543.07 |  | n |  |  |
| SPBC660.10 |  | n |  |  |
| SPBC800.08 |  | n |  |  |
| SPAC23H4.17c |  | n |  |  |
| SPBC16E9.06c |  | n |  |  |
| SPCC736.11 |  | n |  |  |
| SPBC1703.04 |  | n |  |  |
| SPAC30D11.07 |  | n |  |  |
| SPAC13F5.05 |  | n | 5-/+ | n |
| SPAC30.02c |  | n |  |  |
| SPBC342.06c |  | n | 5 | n |
| SPAC13D6.03c |  | n |  |  |
| SPAC2E1P3.04 |  | n |  |  |
| SPAC664.07c |  | n |  |  |
| SPAC57A7.12 |  | n |  |  |
| SPBC4B4.10c |  | n |  |  |
| SPBC1105.14 |  | n |  |  |
| SPAC9G1.10c |  | n |  |  |
| SPAC20H4.07 |  | n |  |  |
| SPCC1450.05c |  | n |  |  |
| SPBC17A3.03c |  | n | 5-/+ | n |
| SPAC19A8.03 |  | n |  |  |
| SPCC4B3.15 |  | n |  |  |
| SPAC25A8.01c |  | n |  |  |
| SPAC926.07c |  | n |  |  |
| SPAC3A11.02 |  | n |  |  |
| SPAC4G9.02 |  | n |  |  |
| SPBC1198.11c |  | n |  |  |
| SPAC11E3.05 |  | n |  |  |
| SPAPB1A10.09 |  | n |  |  |
| SPBC19F8.03c |  | n |  |  |
| SPAC22F8.04 |  | n |  |  |
| SPBC660.14 |  | n |  |  |
| SPCC794.11c |  | n |  |  |
| SPBC1347.03 |  | n |  |  |
| SPBC17D1.07c |  | n |  |  |
| SPAC24C9.15c |  | n |  |  |
| SPBC83.17 |  | n |  |  |
| SPAC19B12.08 |  | n |  |  |
| SPAC22F8.07c |  | n |  |  |
| SPAC15A10.16 |  | n |  |  |
| SPAC26H5.05 |  | n |  |  |
| SPAC3C7.12 |  | n |  |  |
| SPBC25D12.06 |  | n |  |  |
| SPBC354.03 |  | n | 5 | ± |
| SPBC15C4.05 |  | n |  |  |
| SPBC27B12.05 |  | n |  |  |
| SPAC144.14 |  | n |  |  |
| SPAC16E8.12c |  | n |  |  |
| SPAC1687.09 |  | n |  |  |
| SPAC14C4.01c |  | n | 3 | n |
| SPAC13G7.09c |  | n |  |  |
| SPAC144.17c |  | n |  |  |
| SPAC4G9.09c |  | n |  |  |
| SPBC6B1.06c |  | n |  |  |
| SPAC4G9.10 |  | n |  |  |
| SPAC227.18 |  | n |  |  |
| SPAC24C9.07c |  | n |  |  |
| SPBC2D10.13 |  | n | 5-/+ | ± |
| SPBC4B4.03 |  | n |  |  |
| SPAC25G10.05c |  | n |  |  |
| SPBC1711.12 |  | n |  |  |
| SPAC1783.05 |  | n |  |  |
| SPAC343.04c |  | n |  |  |
| SPCC1450.02 |  | n |  |  |
| SPAC1A6.01c |  | n |  |  |
| SPAC25A8.02 |  | n |  |  |
| SPAC29B12.08 |  | n |  |  |
| SPAC27D7.10c |  | n |  |  |
| SPAC26H5.04 |  | n |  |  |
| SPAC2F3.08 |  | n | 3 | ± |
| SPAC23C4.07 |  | n |  |  |
| SPAC30D11.11 |  | n |  |  |
| SPAC24B11.07c |  | n | 5-/+ | n |
| SPAC27E2.07 |  | n |  |  |
| SPAC2F7.04 |  | n | 5± | n |
| SPAC23H3.13c |  | n |  |  |
| SPAC3A11.04 |  | n |  |  |
| SPAC29E6.09 |  | n |  |  |
| SPAC20G8.10c |  | n |  |  |
| SPAC2E1P3.05c |  | n |  |  |
| SPAC25H1.04 |  | n |  |  |
| SPAC3F10.05c |  | n |  |  |
| SPCC1795.09 |  | n |  |  |
| SPBC23G7.04c |  | n |  |  |
| SPAC1D4.03c(aut12) |  | n |  |  |
| SPBC18H10.07 |  | n |  |  |
| SPCC1620.11 |  | n |  |  |
| SPCC188.08c |  | n |  |  |
| SPAC4F10.11 |  | n |  |  |
| SPAC29A4.11 |  | n |  |  |
| SPAC31G5.12c |  | n |  |  |
| SPAP27G11.11c |  | n | 2 | n |
| SPAC977.08 |  | n |  |  |
| SPBC146.10 |  | n |  |  |
| SPBC146.11c |  | n |  |  |
| SPBC1198.01 |  | n |  |  |
| SPBC13G1.12 |  | n |  |  |
| SPBC15D4.08c |  | n |  |  |
| SPBC18E5.07 |  | n |  |  |
| SPBC1604.12 |  | n |  |  |
| SPBC1921.06c |  | n |  |  |
| SPBC18E5.08 |  | n |  |  |
| SPBC16G5.13 |  | n |  |  |
| SPBC16A3.03c |  | n |  |  |
| SPBC16D10.11c |  | n | 5± | n |
| SPBC16H5.13 |  | n |  |  |
| SPBC16E9.12c |  | n | 5 | n |
| SPBC1711.04 |  | n |  |  |
| SPBC1773.09c |  | n |  |  |
| SPBC16C6.01c |  | n |  |  |
| SPBC18E5.09c |  | n |  |  |
| SPBC18E5.10 |  | n |  |  |
| SPBC1711.16 |  | n |  |  |
| SPBC16C6.02c |  | n |  |  |
| SPBC1778.02 |  | n |  |  |
| SPBC1711.09c |  | n |  |  |
| SPBC1718.05 |  | n |  |  |
| SPBC19G7.09 |  | n | 5-/+ | n |
| SPBC2D10.14c |  | n |  |  |
| SPBC2G2.08 |  | n |  |  |
| SPBC29A10.06c |  | n |  |  |
| SPBC31E1.01c |  | n |  |  |
| SPBC31F10.08 |  | n |  |  |
| SPBC2G2.14 |  | n | 5 | ++ |
| SPBC27.05 |  | n |  |  |
| SPBC27.06c |  | n |  |  |
| SPBC418.02 |  | n |  |  |
| SPBC36B7.02 |  | n |  |  |
| SPBC4F6.11c |  | n |  |  |
| SPBC405.03c |  | n |  |  |
| SPBC36B7.06c |  | n |  |  |
| SPBC3B8.08 |  | n |  |  |
| SPBC428.10 |  | n |  |  |
| SPBC29A10.03c |  | n |  |  |
| SPBC342.03 |  | n | 4 | n |
| SPBC336.05c |  | n |  |  |
| SPBC4C3.08 |  | n |  |  |
| SPBC3H7.10 |  | n |  |  |
| SPBC36.06c |  | n |  |  |
| SPBC3E7.16c |  | n |  |  |
| SPBC3B9.09 |  | n |  |  |
| SPBC365.14c |  | n |  |  |
| SPBC428.14 |  | n |  |  |
| SPBC418.01c |  | n |  |  |
| SPBC21C3.17c |  | n |  |  |
| SPBC1A4.04 |  | n |  |  |
| SPBC24C6.10c |  | n |  |  |
| SPBC23G7.14 |  | n |  |  |
| SPBC24C6.04 |  | n |  |  |
| SPBC24C6.08c |  | n |  |  |
| SPBC23G7.15c |  | n |  |  |
| SPBC20F10.03 |  | n | 5± | n |
| SPBC23G7.06c |  | n |  |  |
| SPBC21C3.12c |  | n |  |  |
| SPBC216.06c |  | n |  |  |
| SPBC577.05c |  | n |  |  |
| SPBC336.15 |  | n | 2 | -/+ |
| SPBC1685.15c |  | n |  |  |
| SPBC651.10 |  | n |  |  |
| SPBC106.20 |  | n |  |  |
| SPBC902.03 |  | n |  |  |
| SPBP8B7.02 |  | n |  |  |
| SPBC21B10.06c |  | n | 1 | n |
| SPBP8B7.28c |  | n |  |  |
| SPBC947.03c |  | n |  |  |
| SPBC9B6.02c |  | n |  |  |
| SPBP8B7.13 |  | n |  |  |
| SPCC1020.11c |  | n |  |  |
| SPBP23A10.14c |  | n |  |  |
| SPCC11E10.06c |  | n |  |  |
| SPBC1711.11 |  | n |  |  |
| SPAPJ696.01c |  | n |  |  |
| SPCC1672.04c |  | n |  |  |
| SPCC1223.13 |  | n |  |  |
| SPBP23A10.03c |  | n |  |  |
| SPBC1289.11 |  | n |  |  |
| SPBC18H10.11c |  | n |  |  |
| SPCC1393.05 |  | n | 2 | ± |
| SPCC1494.10 |  | n |  |  |
| SPCC132.03 |  | n |  |  |
| SPCC162.06c |  | n |  |  |
| SPBC776.04 |  | n | 1 | n |
| SPBC8D2.18c |  | n |  |  |
| SPBC646.08c |  | n |  |  |
| SPCC1393.11 |  | n |  |  |
| SPBC651.05c |  | n |  |  |
| SPCC1442.13c |  | n |  |  |
| SPCC188.07 |  | n |  |  |
| SPCC1919.13c |  | n |  |  |
| SPCC285.13c |  | n |  |  |
| SPBC651.07 |  | n |  |  |
| SPCC18B5.02c |  | n |  |  |
| SPBC887.13c |  | n |  |  |
| SPCC1919.07 |  | n |  |  |
| SPCC162.02c |  | n |  |  |
| SPCC31H12.02c |  | n |  |  |
| SPCC1742.01 |  | n |  |  |
| SPCC622.01c |  | n |  |  |
| SPCC1840.04 |  | n |  |  |
| SPCC553.01c |  | n | 5± | ± |
| SPCC188.13c |  | n |  |  |
| SPBP22H7.04 |  | n |  |  |
| SPCC5E4.10c |  | n |  |  |
| SPCC584.01c |  | n |  |  |
| SPCC126.15c |  | n |  |  |
| SPBC3B8.10c |  | n | 1 | n |
| SPBC4.06 |  | n |  |  |
| SPAC17A2.09c |  | n |  |  |
| SPCC777.13 |  | n |  |  |
| SPCC1827.08c |  | n |  |  |
| SPCC622.18 |  | n | 5 | ND |
| SPCC1840.02c |  | n |  |  |
| SPBC3H7.11 |  | n |  |  |
| SPBC3H7.05c |  | n |  |  |
| SPBC646.02 |  | n |  |  |
| SPCC790.02 |  | n |  |  |
| SPCC663.07c |  | n | 5± | n |
| SPCC663.12 |  | n |  |  |
| SPCC63.02c |  | n |  |  |
| SPCC645.11c |  | n |  |  |
| SPCC330.12c |  | n |  |  |
| SPBPB2B2.09c |  | n |  |  |
| SPAC26A3.14c |  | n |  |  |
| SPAC17C9.13c |  | n |  |  |
| SPAC22F3.02 |  | n |  |  |
| SPBC16D10.08c |  | n |  |  |
| SPAC1002.07c |  | n |  |  |
| SPBC106.10 |  | n |  |  |
| SPAC13G6.14 |  | n |  |  |
| SPBC31F10.15c |  | n | 5-/+ | ± |
| SPAC7D4.05 |  | n | 3 | n |
| SPAC4G8.15c |  | n |  |  |
| SPAPYUG7.03c |  | n | 5 | n |
| SPBC30D10.09c |  | n |  |  |
| SPBC27B12.11c |  | n |  |  |
| SPBC8D2.04 |  | n | 1± | n |
| SPCC162.12 |  | n |  |  |
| SPBC1921.05 |  | n |  |  |
| SPBC18E5.13 |  | n |  |  |
| SPCC1753.02c |  | n |  |  |
| SPAC821.09 |  | n |  |  |
| SPBC19C2.14 |  | n |  |  |
| SPBC1105.02c |  | n |  |  |
| SPAC23A1.19c |  | n |  |  |
| SPCC1259.08 |  | n |  |  |
| SPBC2G5.04c |  | n |  |  |
| SPCC1682.13 |  | n |  |  |
| SPBC32H8.07 |  | n |  |  |
| SPAC57A10.03 |  | n |  |  |
| SPAC1610.03c |  | n |  |  |
| SPCC550.10 |  | n |  |  |
| SPCPB1C11.03 |  | n |  |  |
| SPAC343.18 |  | n | 5-/+ | n |
| SPBPB10D8.06c |  | n |  |  |
| SPCC338.16 |  | n |  |  |
| SPBC32C12.03c |  | n |  |  |
| SPBC530.05 |  | n |  |  |
| SPBC1734.07c |  | n |  |  |
| SPBC19F8.04c |  | n |  |  |
| SPBC27B12.07 |  | n |  |  |
| SPBC30B4.06c |  | n |  |  |
| SPAC17G8.14c |  | n |  |  |
| SPBC16H5.04 |  | n |  |  |
| SPAC20H4.10 |  | n |  |  |
| SPAC27F1.08 |  | n |  |  |
| SPBC21B10.13c |  | n |  |  |
| SPBC16A3.12c |  | n |  |  |
| SPAC17D4.03c |  | n |  |  |
| SPBC29A3.13 |  | n |  |  |
| SPAC1F5.09c |  | n | 2 | n |
| SPAC24B11.12c |  | n |  |  |
| SPBC17G9.12c |  | n |  |  |
| SPBC215.07c |  | n | 3 | n |
| SPAC15E1.05c |  | n | 5-/+ | n |
| SPAC926.09c |  | n | 1± | -/+ |
| SPBC28F2.08c |  | n |  |  |
| SPAC1F5.08c |  | n |  |  |
| SPAC4A8.04 |  | n |  |  |
| SPAC8C9.12c |  | n |  |  |
| SPAC3A12.06c |  | n |  |  |
| SPAC222.05c |  | n | 5± | n |
| SPCC1393.10 |  | n |  |  |
| SPAC6G10.11c |  | n |  |  |
| SPAC3H1.06c |  | n |  |  |
| SPBC839.13c |  | n |  |  |
| SPBC887.04c |  | n |  |  |
| SPAC328.05 |  | n |  |  |
| SPAC8E11.03c |  | n |  |  |
| SPBC21B10.12 |  | n |  |  |
| SPAC22G7.04 |  | n |  |  |
| SPAC16A10.05c |  | n |  |  |
| SPAC6B12.12 |  | n | 4 | n |
| SPBC215.05 |  | n |  |  |
| SPCC594.04c |  | n |  |  |
| SPAC31A2.13c |  | n |  |  |
| SPAC1782.01 |  | n |  |  |
| SPBC23G7.11 |  | n |  |  |
| SPAC521.02 |  | n |  |  |
| SPBC11B10.10c |  | n |  |  |
| SPAC630.09c |  | n | 5 | n |
| SPBC1105.08 |  | n |  |  |
| SPCC1753.03c |  | n |  |  |
| SPAP14E8.04 |  | n | 3 | n |
| SPAC6G9.13c |  | n |  |  |
| SPAC25B8.19c |  | n |  |  |
| SPBC30D10.04 |  | n |  |  |
| SPAC750.01 |  | n |  |  |
| SPAC922.05c |  | n |  |  |
| SPBC1604.19c |  | n |  |  |
| SPCC1393.09c |  | n | 5-/+ | n |
| SPCC16A11.07 |  | n |  |  |
| SPAC1805.14 |  | n |  |  |
| SPBC1E8.03c |  | n |  |  |
| SPAC8E11.07c |  | n | 1 | -/+ |
| SPAC6B12.16 |  | n |  |  |
| SPBC12D12.07c |  | n |  |  |
| SPBP35G2.02 |  | n |  |  |
| SPBC23G7.12c |  | n |  |  |
| SPAC23H4.07c |  | n |  |  |
| SPAC23C11.02c |  | n |  |  |
| SPCC188.02 |  | n |  |  |
| SPBC21H7.04 |  | n |  |  |
| SPAC22F3.04 |  | n |  |  |
| SPBC27B12.10c |  | n |  |  |
| SPCC18B5.07c |  | n | 5 | n |
| SPBC12C2.08 |  | n |  |  |
| SPBC146.13c |  | n |  |  |
| SPAC1834.07 |  | n | 5-/+ | n |
| SPAC24H6.03(cul3) |  | y |  |  |
| SPBC336.03(efc25) |  | y |  |  |
| SPBC365.06(pmt3) |  | y |  |  |
| SPCC126.08c |  | y |  |  |
| SPAC227.01c |  | y |  |  |
| SPBC428.08c(clr4) |  | y | 5 | + |
| SPAC22H10.07(scd2) |  | y |  |  |
| SPAC6B12.07c |  | y |  |  |
| SPBC25B2.1(pof2) |  | y |  |  |
| SPBC1773.12 |  | y |  |  |
| SPBC29A3.05(vps71) |  | y |  |  |
| SPAC110.02(pds5) |  | y | 5 | + |
| SPBC56F2.08c |  | y |  |  |
| SPBP35G2.14 |  | y |  |  |
| SPBC4F6.12(pxl1) |  | y |  |  |
| SPAC821.07c(moc3) |  | y |  |  |
| SPAC3H1.05 |  | y |  |  |
| SPBC16C6.11(rpl3201) |  | y |  |  |
| SPAC26A3.04(rpl2002) |  | y |  |  |
| SPAC17H9.19c(cdt2) |  | y |  |  |
| SPAC4F8.01(did4) |  | y |  |  |
| SPAC23H3.08c(bub3) |  | y |  |  |
| SPBC28F2.10c(ngg1) |  | y |  |  |
| SPAC824.04 |  | y |  |  |
| SPCC1682.12c(ubp16) |  | y |  |  |
| SPAC30D11.04c(nup214) |  | y |  |  |
| SPCC736.04c(gma12) |  | y |  |  |
| SPBC19C7.10(bqt4) |  | y |  |  |
| SPBC342.01c(alg6) |  | y |  |  |
| SPBC211.06(gfh1) |  | y |  |  |
| SPBC215.03c(csn1) |  | y |  |  |
| SPBC557.02c |  | y |  |  |
| SPAC17G8.05(med20) |  | y |  |  |
| SPCC830.06 |  | y |  |  |
| SPCC338.08(ctp1) |  | y |  |  |
| SPCC576.14(dph5) |  | y |  |  |
| SPAC19B12.13(cox1102) |  | y |  |  |
| SPBC1921.07c(sgf29) |  | y |  |  |
| SPAC631.02 |  | y | 2 | ++ |
| SPBC1604.08c(imp1) |  | y |  |  |
| SPBC1778.01c(zuo1) |  | y |  |  |
| SPBC887.10(mcs4) |  | y |  |  |
| SPCC550.11 |  | y |  |  |
| SPAC13G7.07(arb2) |  | y | 2 | + |
| SPBP4H10.09(rsv1) |  | y |  |  |
| SPAC13G7.13c(msa1) |  | y |  |  |
| SPBC16C6.04 |  | y |  |  |
| SPAC23A1.11(rpl1602) |  | y |  |  |
| SPAC1142.07c(vps32) |  | y |  |  |
| SPBC1711.05 |  | y |  |  |
| SPBC2A9.08c(sec22) |  | y |  |  |
| SPAC9E9.09c |  | y |  |  |
| SPBC16E9.07(mug100) |  | y | 4 | n |
| SPBC1604.20c(tea2) |  | y | 1 | n |
| SPBC29A3.02c(his7) |  | y |  |  |
| SPAC17A5.02c(dbr1) |  | y |  |  |
| SPBC2G2.03c(abh1) |  | y |  |  |
| SPCC1450.03 |  | y |  |  |
| SPBC21C3.19 |  | y |  |  |
| SPBC1718.04 |  | y |  |  |
| SPBC1773.11c( mug89) |  | y |  |  |
| SPBC1198.08 |  | y |  |  |
| SPBC1711.14(rec15) |  | y |  |  |
| SPBPJ4664.05 |  | y |  |  |
| SPCP20C8.02c |  | y |  |  |
| SPAC27D7.03c(mei2) |  | y |  |  |
| SPBC1706.01 |  | y, n |  |  |
| SPBC1734.12c |  | y, n |  |  |
| SPBC31E1.02c |  | y, n |  |  |
| SPBC1105.10 |  | y, n | 1 | n |
| SPCC1919.01 |  | y, n |  |  |
| SPAC3G9.05 |  | y, n |  |  |
| SPCP1E11.02 |  | y, n |  |  |
| SPBP23A10.10 |  | y, n |  |  |
| SPBC1778.10c |  | y, n |  |  |
| SPAC12B10.14c |  | y, n |  |  |
| SPCC162.10 |  | y, n |  |  |
| SPBC4F6.06 |  | y, n |  |  |
| SPAC22G7.08 |  | y, n |  |  |
| SPBC17D11.04c |  | y, n |  |  |
| SPBC2D10.17 |  | y, n |  |  |
| SPBC30B4.04c |  | y, n |  |  |
| SPAC16C9.05 |  | y, n |  |  |
| SPAC2F7.10 |  | y, n |  |  |
| SPAC23A1.07 |  | y, n |  |  |
| SPAC22F3.12c |  | y, n |  |  |
| SPBC3B8.02 |  | y, n |  |  |
| SPBC2D10.06 |  | y, n |  |  |
| SPAC6G9.10c |  | y, n |  |  |
| SPAC18G6.15 |  | y, n | 1 | n |
| SPAC18G6.02c |  | y, n |  |  |
| SPAC3A11.13 |  | y, n |  |  |
| SPCC1223.05c |  | y, n |  |  |
| SPCC663.04 |  | y, n |  |  |
| SPAC2F3.13c |  | y, n |  |  |
| SPBP35G2.08c |  | y, n |  |  |
| SPBC20F10.10 |  | y, n |  |  |
| SPAC17A2.13c |  | y, n |  |  |
| SPBC56F2.05c |  | y, n |  |  |
| SPAC227.07c |  | y, n |  |  |
| SPCC61.05 |  | y, n |  |  |
| SPBC21D10.10 |  | y, n |  |  |
| SPCC1322.08 |  | y, n |  |  |
| SPAC3H5.10 |  | y, n |  |  |
| SPAC959.08 |  | y, n |  |  |
| SPBC1685.01 |  | y, n |  |  |
| SPAC1834.05 |  | y, n |  |  |
| SPBC119.12 |  | y, n |  |  |
| SPBC2F12.12c |  | y, n | 5 | ++ |
| SPBC215.02 |  | y, n |  |  |
| SPBC20F10.06 |  | y, n |  |  |
| SPAC22F3.08c |  | y, n |  |  |
| SPAC16E8.17c |  | y, n |  |  |
| SPAC3A11.03 |  | y, n |  |  |
| SPAC31G5.17c |  | y, n |  |  |
| SPCC1235.13 |  | y, n |  |  |
| SPAC767.01c |  | y, n |  |  |
| SPBC13E7.06 |  | y, n | 5 | + |
| SPBC1718.03 |  | y, n |  |  |
| SPBC354.07c |  | y, n |  |  |
| SPBC106.01 |  | y, n |  |  |
| SPCC126.04c |  | y, n | 5 | n |
| SPCC1393.13 |  | y, n |  |  |
| SPCC162.05 |  | y, n |  |  |
| SPCC1322.03 |  | y, n |  |  |
| SPCC18.17c |  | y, n |  |  |
| SPCC417.02 |  | y, n |  |  |
| SPBC215.14c |  | y, n |  |  |
| SPCC825.03c |  | y, n |  |  |
| SPAC11G7.02 |  | y, n |  |  |
| SPAC824.02 |  | y, n |  |  |
| SPAC1A6.04c |  | y, n |  |  |
| SPAC15A10.06 |  | y, n |  |  |
| SPAC31A2.11c |  | y, n |  |  |
| SPAC8E11.02c |  | y, n |  |  |
| SPCC794.07 |  | y, n |  |  |
| SPAC1071.04c |  | y, n |  |  |
| SPAC1006.03c |  | y, n |  |  |
| SPCC4G3.14 |  | y, n |  |  |
| SPCC576.01c |  | y, n |  |  |
| SPBC119.06 |  | y, n |  |  |
| SPCC4G3.14 |  | y, n |  |  |
| SPCC736.08 |  | y, n |  |  |
| SPBP8B7.22 |  | y, n |  |  |
| SPBC651.09c |  | y, n |  |  |
| SPAC977.09c |  | y, n |  |  |
| SPCC895.07 |  | y, n |  |  |
| SPBC1198.12 |  | y, n |  |  |
| SPBC1718.07c |  | y, n |  |  |
| SPAC29B12.06c(rcd1) |  | y, y |  |  |
| SPCC31H12.08c(ccr4) |  | y, y |  |  |
| SPCC18.06c(caf1) |  | y, y |  |  |
| SPCC1906.02c |  |  | 6 | n |
| SPCC11E10.08 |  |  | 5-/+ | ++ |
| SPBC800.05c |  |  | 5 | n |
| SPAC227.06 |  |  | 5 | n |
| SPAC18G6.04c |  |  | 3 | n |
| SPAC4F10.19c |  |  | 3 | n |
| SPAC3G9.01 |  |  | 5 | -/+ |
| SPAC977.02 |  |  | 5 | n |
| SPAC4D7.07c |  |  | 5-/+ | -/+ |
| SPAC4G9.15 |  |  | 3 | n |
| SPAC3H1.07 |  |  | 5± | n |
| SPAC521.03 |  |  | 5± | n |
| SPAC9E9.11 |  |  | 5 | n |
| SPAC823.05c |  |  | 4 | ND |
| SPBC25H2.03 |  |  | 5-/+ | n |
| SPBC21.02 |  |  | 1 | n |
| SPBC1604.03c |  |  | 3 | n |
| SPBC409.10 |  |  | 1± | n |
| SPCC553.03 |  |  | 5-/+ | n |
| SPCC594.05c |  |  | 5-/+ | n |
| SPCC1739.06c |  |  | 1± | n |
| SPCC1672.12c |  |  | 5-/+ | -/+ |
| SPAC926.03 |  |  | 5-/+ | n |
| SPBC19G7.18c |  |  | 5 | n |
| SPBC27.02c |  |  | 1 | ++ |
| SPCC16C4.20c |  |  | 5-/+ | n |
| SPBC24C6.05 |  |  | 5-/+ | n |
| SPAC4A8.09c |  |  | 5-/+ | ND |
| SPAC11G7.04 |  |  | 5 | n |
